# CD44v6-competent tumor exosomes promote motility, invasion and cancer-initiating cell marker expression in pancreatic and colorectal cancer cells

**DOI:** 10.18632/oncotarget.10580

**Published:** 2016-07-13

**Authors:** Zhe Wang, Anja von Au, Martina Schnölzer, Thilo Hackert, Margot Zöller

**Affiliations:** ^1^ Tumor Cell Biology, University Hospital of Surgery, Heidelberg, Germany; ^2^ Proteome Analysis Department, German Cancer Research Center, Heidelberg, Germany; ^3^ Section Pancreas Research, University Hospital of Surgery, Heidelberg, Germany

**Keywords:** CD44v6, exosomes, adhesion molecules, metastasis, cancer stem cells

## Abstract

Cancer-initiating cells (CIC) account for metastatic spread, which may rely mostly on CIC exosomes (TEX) that affect host cells and can transfer CIC features into Non-CIC. The CIC marker CD44 variant isoform v6 (CD44v6) being known for metastasis-promotion, we elaborated in cells its contribution to migration and invasion and in TEX the tranfer of migratory and invasive capacity to Non-CIC, using a CD44v6 knockdown (CD44v6^kd^) as Non-CIC model.

A CD44v6^kd^ in human pancreatic and colorectal cancer (PaCa, CoCa) lines led to loss of CIC characteristics including downregulation of additional CIC markers, particularly Tspan8. This aggravated the loss of CD44v6-promoted motility and invasion. Loss of motility relies on the distorted cooperation of CD44v6 and Tspan8 with associated integrins and loss of invasiveness on reduced protease expression. These deficits, transferred into TEX, severely altered the CD44v6^kd^-TEX composition. As a consequence, unlike the CIC-TEX, CD44v6^kd^ TEX were not taken up by CD44v6^kd^ cells and CIC. The uptake of CIC-TEX was accompanied by partial correction of CIC marker and protease expression in CD44v6^kd^ cells, which regained migratory, invasive and metastatic competence. CIC-TEX also fostered angiogenesis and expansion of myeloid cells, likely due to a direct impact of CIC-TEX on the host, which could be supported by reprogrammed CD44v6^kd^ cells.

Taken together, the striking loss of tumor progression by a CD44v6^kd^ relies on the capacity of CD44v6 to cooperate with associating integrins and proteases and its promotion of additional CIC marker expression. The defects by a CD44v6^kd^ are efficiently corrected upon CIC-TEX uptake.

## INTRODUCTION

Cancer related mortality is mostly du to metastatic spread [1, 2]. One of the first steps in the metastatic cascade is the emigration of individual tumor cells out of the primary tumor mass [3]. There is evidence that the subpopulation of cancer initiating cells (CIC) accounts for tumorigenicity, tumor recurrence and metastasis [4, 5]. Beside other methods, CIC can be enriched by their capacity to grow as spheres or holoclones, which are supposed to reflect self renewal capacity (5a, 5b). While holoclones contain small, tightly packed cells, meroclones contain larger, loosely attached transit cells and paraclones, large, flattened differentiating cells [6, 7]. CIC are also defined by their markers [8], CD44v6 being a CIC marker in pancreatic and colorectal adenocarcinoma (PaCa, CoCa) [9–13].

CD44v6 is a splice variant of CD44, an abundantly expressed adhesion molecule and the prime receptor for hyaluronan (HA) [14]. The globular N-terminal region contains additional binding sites for collagen (coll), laminin (LN), fibronectin (FN) and selectins [15, 16]. The CD44v6 exon product binds several growth factors [17], whereby CD44v6 takes over a coordinating role in receptor tyrosine kinase (RTK) activation [18]. Thus, CD44v6 promotes MET phosphorylation, which requires the cytoplasmic tail of CD44 and the interaction with ERM (ezrin, radixin, moesin) proteins for activation of the Ras-MAPK pathway [19]. CD44v6 binding to the extracellular matrix (ECM) activates the PI3K-Akt pathway and Wnt/β-catenin signaling [20, 21] and regulates MET transcription [22]. Similar observations account for insulin-like growth factor-1 and platelet-derived growth factor receptor (PDGFR) activation through HA-stimulated CD44 in transformed cells [23]. CD44v6 also binds osteopontin (OPN), important for cell recruitment and motility [13, 24]. The cytoplasmic tail of CD44 contains binding sites for the cytoskeleton linkers ankyrin and ERM proteins. The N-terminus of activated ERM proteins binds to CD44 and the C-terminus binds to F-actin, linking CD44 to the actin cytoskeleton [25]. The binding of CD44 to cytoskeletal linker proteins influences signaling pathways downstream of CD44, which expands the range of CD44-mediated functions. Finally, CD44 O-glycosylation, the transmembrane region and the cytoplasmic tail affect the membrane subdomain localization. Recruitment into glycolipid-enriched membrane microdomains (GEM) [26] has great bearing on the interaction of CD44 with extracellular ligands and the association with other transmembrane and cytoplasmic molecules and is crucial for the activity of CD44 in signal transduction and migration [27, 28].

CD44 / CD44v6 also are engaged in matrix assembly, HA-CD44 association modifying the matrix to support colonization [29]. A CD44v6^kd^ tumor line secretes a matrix that does not support adhesion of CD44v^wt^ or CD44v6^kd^ cells [30], the CD44v6^kd^ cells displaying reduced HA synthase 3 (HAS3) expression, but abundantly secrete hyaluronidase (Hyal) [31]. High HAS3 expression correlates with tumor aggressiveness [32], whereas low molecular weight HA affects adhesion and catcher activity of the matrix. Finally, the production of uPAR, MMP2 and MMP9 is stimulated by the interaction between HA and CD44, the CD44 intracellular domain (ICD) binding to a MMP9 promoter response element [33]. CD44 aggregation via HA facilitates MMP binding and proMMP2 and proMMP9 activation through CD44-associated MMP14 [33, 34]. As cell-bound MMPs are protected from their inhibitors, focal degradation of the ECM forms space for invading tumor cells [35]. In addition, transforming growth factor β (TGFβ) activation through CD44-associated MMP9 promotes angiogenesis and invasion [36].

Exosomes (Exo), small 30-100nm vesicles, derive from multivesicular bodies, which can fuse with the plasma membrane and release their intraluminal vesicles as exosomes [37]. Exo are composed of a lipid bilayer, they contain transmembrane and cytosolic proteins, mRNA, non-coding RNA and DNA [38]. Some proteins are constitutive Exo components, like tetraspanins, protein complexes engaged in vesicle formation and vesicle transport and signal transduction molecules that attach to the lipid bilayer. Exo components are function competent [39, 40]. They act via binding- and uptake-induced target cell activation and reprogramming [41] and are discussed as powerful intercellular communicators [39, 42, 43]. Tumor exosomes (TEX) were demonstrated to contribute to angiogenesis, tumor progression, premetastatic niche formation and immunosuppression [44–47].

We here describe that TEX CD44v6 contributes to tumor progression by cooperating with integrins and proteases and by regulating additional CIC marker, particularly Tspan8, expression in non-CIC.

## RESULTS

CD44v6 is a metastogen and a CIC marker in several malignancies. Whether deficits of CD44v6^kd^ cells can be corrected by CIC-derived TEX is unknown and was elaborated for human PaCa and CoCa progression.

### Controlling for the contribution of CD44v6 to cancer initiating cell characteristics

The mode of activity of CD44v6 as a CIC biomarker is still disputed and a team play between different activities appears most likely. Aiming in the long run to unravel these networks, including the contribution of TEX, we started evaluating the overall impact of a CD44v6^kd^ on CIC features *in vitro* and *in vivo*.

**Figure 1:**
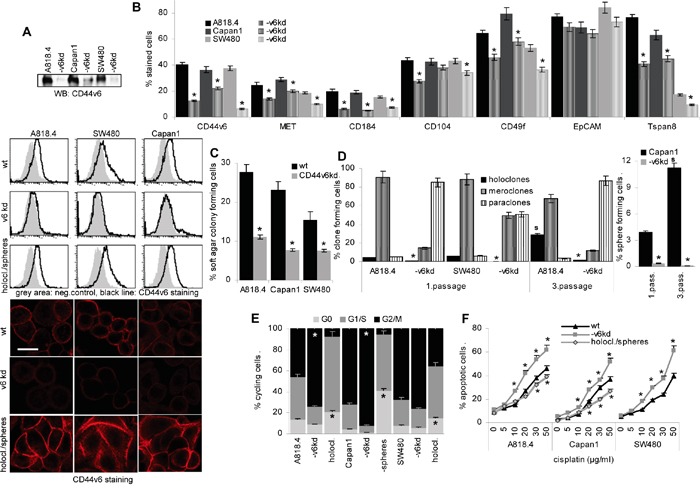
CD44v6 is a pancreatic and colorectal cancer initiating cell marker **A.** A CD44v6^kd^ in A818.4, Capan1 and SW480 cells was controlled by WB, flow-cytometry and confocal microscopy (scale bar: 10μm), where CD44v6 expression in spheres and holoclones (1. passage) was included. **B.** Flow-cytometry analysis of the CIC markers CD184, CD104, CD49f, EpCAM and Tspan8. **C.** Anchorage-independent growth was evaluated by colony formation in soft agar. **D.** The capacity of A818.4 and SW480 wt and CD44v6^kd^ cells to grow as holoclones and of Capan1 cells to grow as spheres was evaluated in 1^st^ and 3^rd^ passage cells, where holoclones/spheres were dispersed and seeded again under the respective culture conditions. **E.** Cell cycle progression was evaluated in wt and CD44v6^kd^ cells and spheres/holoclones by PI staining. **F.** Apoptosis was evaluated by AnnV/PI staining after 48h culture in the presence of titrated amounts of cisplatin. (B-F) Mean±SD of three independent assays are shown, significant differences to wt cells: *. CD44v6 was efficiently knocked down in the three lines. The CD44v6^kd^ affected expression of additional CIC markers. The CD44v6^kd^ lines show a reduced capacity for anchorage-independent growth and sphere or holoclone formation. Cell cycle progression is slow in spheres/holoclones and accelerated in CD44v6^kd^ cells. Apoptosis-resistance is strengthened in holoclones/spheres, but reduced in CD44v6^kd^ cells. The findings confirm the engagement of CD44v6 in stem cell growth characteristics.

CD44v6 was knocked down in the PaCa lines A818.4 and Capan1 and the CoCa line SW480, the efficacy of one representative CD44v6^kd^ clone is demonstrated by Western blot (WB), flow-cytometry and confocal microscopy. CD44v6 is efficiently reduced in all three lines, though more efficient in A818.4 and SW480 than Capan1 cells. Notably, flow-cytometry and confocal microscopy revealed upregulated CD44v6 expression in spheres (Capan1) and holoclones (A818.4, SW480) (Figure 1A). The CD44v6^kd^ cells show a reduced capacity to sprite, the more round cells adhering less efficiently to plastic (Supplementary Figure 1A). The CD44v6^kd^ is accompanied by a distinct reduction in MET, Tspan8, CXCR4 (CD184) and a moderate to weak reduction in β4 (CD104), α6 (CD49f), prominin-1 (CD133) and claudin (cld)7 expression. EpCAM expression is not affected. With the exception of CD133 that expression is low in SW480 cells, this accounts for A818.4, Capan1 and SW480 cells (Figure 1B, Table 1A). The capacity of colony formation in soft agar is significantly impaired (Figure 1C, Supplementary Figure 1B), and the capacity to form spheres or holoclones, additional CIC features, suggested to be linked to self-renewal, is nearly abolished. When reculturing spheres or holoclones from wt cells, the percentage of spheres and holoclones increased, while the few spheres or holoclones from CD44v6^kd^ cells did not survive a second round of sphere or holoclone forming culture condition (Figure 1D, Supplementary Figure 1C). Furthermore, a higher percentage of CD44v6^kd^ than wt cells is in G2/M, whereas a higher percentage of Capan1 spheres and A818.4 and SW480 holoclones are in G0, indicating the more rapid progression of CD44v6^kd^ cells through the cell cycle and the more resting state of CIC-enriched populations (Figure 1E, Supplementary Figure 1D). However, as evaluated by CFSE dilution, the proliferation rate did not strongly differ between wt cells and spheres/holoclones (Supplementary Figure 1E). Finally, apoptosis resistance was controlled by AnnV/PI staining after culture in the presence of increasing doses of cisplatin. Apoptosis resistance of CD44v6^kd^ cells is significantly reduced and slightly increased in holoclones and spheres (Figure 1F, Supplementary Figure 1F).

**Table 1:** Proteome analysis: CD44v6 related differences

A: Recovery of CIC markers					
name	significant protein matches				full name^1^
	A818.4	A818.4-v6^kd^	Capan1	Capan1-v6^kd^	
CD44	187	18	186	79	**CD44 antigen**
MET	12	0	8	0	**Hepatocyte growth factor receptor**
CXCR4	43	0	38	1	**CXC chemokine receptor 4, CD184**
ITGA6	367	192	265	178	Integrin α6, CD49f
ITGB4	297	118	214	100	**Integrin β4, CD104**
TSAN8	73	8	84	22	**Tetraspanin 8**
EPCAM	166	157	152	81	Epithelial cell adhesion molecule
CLDN7	112	52	91	38	**Claudin7**
PROM1	242	11	195	36	**Prominin 1, CD133**

B: Distinct recovery of adhesion molecules					
name	significant protein matches				full name^1^
	A818.4	A818.4-v6^kd^	Capan1	Capan1-v6^kd^	
**Tetraspanins**					
CD9	118	194	101	100	Tetraspanin CD9
CD63	141	96	136	75	Tetraspanin CD63
**CD81**	328	119	280	118	**Tetraspanin CD81**
CD82	144	63	132	82	Tetraspanin CD82
CD151	44	18	38	24	Tetraspanin CD151
**TSN1**	221	55	169	82	**Tetraspanin 1**
TSN3	29	6	25	38	**Tetraspanin 3**
TSN5	17	14	15	1	**Tetraspanin 5**
**TSN6**	92	3	98	18	**Tetraspanin 6**
**TSN8**	73	8	84	22	**Tetraspanin 8**
**TSN9**	45	0	30	3	**Tetraspanin 9**
**TSN14**	123	28	100	42	**Tetraspanin 14**
**TSN15**	89	10	74	33	**Tetraspanin 15**
**TSN33**	15	0	12	3	**Tetraspanin 33**
**Integrins**					
ITB1	196	100	165	121	Integrin β1
ITB3	6	6	6	10	Integrin β3
**ITB4**	297	118	214	100	**Integrin β4**
**ITB5**	75	36	82	15	**Integrin β5**
**ITB6**	5	0	12	0	**Integrin β6**
**others (downregulated)**					
CD47	26	8	18	40	**Leukocyte surface antigen CD47**
**CCRL2**	11	1	10	1	**CC chemokine receptor like 2**
**CXCR4**	43	0	38	1	**CXC chemokine receptor 4**
DAF	22	8	15	16	**Compl. decay accelerating factor**
**LEG4**	40	0	57	4	**Galectin 4**
LIN7C	17	16	14	7	**Protein LIN-7 homolog C**
MCP	17	0	18	21	**Membrane cofactor protein, CD46**
**MFGM**	210	45	229	114	**Lactadherin, MFGE8**
**MUC18**	72	35	95	11	**Cell surface glycoprotein MUC18**
**others (upregulated)**					
**BGH3**	14	179	9	42	**TGFβ-induced protein Ig-h3**
CD99	5	13	2	3	**CD99 antigen**
CTNA1	2	24			**Catenin α1**
CTNB1	0	45			**Catenin β1**
CXAR	18	55	18	12	**Coxsackie- & adenovirus receptor**
**ICAM1**	26	56	12	29	**Intercellular adhesion molecule 1**
**LEG3**	6	43	2	33	**Galectin 3**
LEG7	0	13			**Galectin-7**
LEG9B			0	10	**Galectin-9B**
SDC4	6	4	7	40	**Syndecan-4**

C: Distinct recovery of proteases						
name	significant protein matches				full name^1^	activity
	A818.4	A818.4-v6kd	Capan1	Capan1-v6kd		
downregulated in both CD44v6^kd^ lines						
ADA10	436	113	369	157	**Disintegrin & MMP domain-cont.10**	metalloprotease
CAN5	29	0	26	5	**Calpain-5**	cysteine protease
CAN7	32	0	15	4	**Calpain-7**	cysteine protease
DPEP1	23	0	20	4	**Dipeptidase 1**	metalloprotease
DPP4	178	16	171	81	**Dipeptidyl peptidase 4, CD26**	cysteine protease
FA5	28	6	21	12	Coagulation factor V	metallo-, serine protease
GGT1	31	1	25	10	**Gamma-glutamyltranspeptidase 1**	protease
GRAN	11	0	10	5	**Grancalcin**	cysteine protease
MFGM	210	45	229	134	Lactadherin	metallo-, serine protease
MMP15	11	0	10	1	**Metalloproteinase 15**	metalloprotease
PDCD6	107	54	109	49	**Programmed cell death protein 6**	cysteine protease
SORCN	42	30	34	15	Sorcin	cysteine protease
downregulated in one CD44v6^kd^ line						
ADAM9	11	2	2	4	**Disintegrin & MMP domain-cont.9**	metalloprotease
DAF	22	8	15	16	**Complement decay-accel. factor, CD55**	metallo-, serine protease
MCP	17	0	18	21	**Membrane cofactor protein, CD46**	metallo-, serine protease
MMP7	10	0	18	58	**Metalloproteinase 7**	metalloprotease
TRFM	24	33	13	4	**Melanotransferrin**	serine protease
upregulated in both CD44v6^kd^ lines						
CATD	1	12	0	5	**Cathepsin D**	aspartic protease
CPNS1	12	30	13	26	**Calpain small subunit 1**	cysteine protease
LCAP	6	38	5	10	**Leucyl-cystinyl aminopeptidase**	metalloprotease
PAI1	0	77	0	23	**plasminogen activator inhibitor**	serine protease inhibitor
PSA	6	27	4	11	**Puromycin-sensitive aminopeptidase**	metalloprotease
TINAL	8	33	21	98	**Tubulointerstitial nephritis antigen-like**	cysteine protease
upregulated in one CD44v6^kd^ line						
CATC	6	4	6	16	**Dipeptidyl peptidase 1**	cysteine protease
GGH	0	24	0	1	**Gamma-glutamyl hydrolase**	cysteine protease
KLK10	0	0	0	18	**Kallikrein-10**	serine prot., protease inhib.
LKHA4	0	10	0	0	**Leukotriene A-4 hydrolase**	metalloprotease
LG3BP	373	482	401	789	Galectin-3-binding protein	serine protease
LOXL2	0	1	0	10	**Lysyl oxidase homolog 2**	serine protease
PA2G4	0	12	0	1	**Proliferation-associated protein 2G4**	metalloprotease
PARK7	4	32	3	0	**Protein DJ-1**	cysteine protease
PCSK9	0	5	1	10	**Proprotein convertase subtilisin type 9**	serine protease
PRPK	0	10	3	6	**TP53-regulating kinase**	metalloprotease
TIMP1	0	1	2	10	**Metalloproteinase inhibitor 1**	metalloprotease inhibitor
no significant regulation						
ST14	99	82	111	69	Suppressor of tumorigenicity 14 protein	serine protease inhib.
UPAR	45	38	42	39	Urokinase receptor	serine protease receptor

D: Distinct recovery of angiogenesis-related molecules					
name	significant protein matches				full name
	A818.4	A818.4-v6kd	Capan1	Capan1-v6kd	
downregulated in CD44v6^kd^					
EPHB3	201	9	130	19	**Ephrin type-B receptor 3**
EFNB1	112	13	106	34	**Ephrin-B1**
EPHB2	60	0	46	18	**Ephrin type-B receptor 2**
SRC	46	0	37	8	**Proto-oncogene tyrosine-protein kinase Src**
RASN	36	18	28	14	**GTPase NRas**
CRK	6	1	3	1	Adapter molecule crk, binds Ephrin receptor
CRKL	2	0	2	0	Crk-like protein
EFNB2	50	0	26	27	**Ephrin-B2**
JAG1	4	2	0	0	Protein jagged-1
upregulated in CD44v6^kd^					
CTNB1	0	45	0	0	**Catenin beta-1**
HSPB1	4	27	7	16	**Heat shock protein beta-1**
WNT5A	0	2	0	0	Protein Wnt-5a
no significant regulation in CD44v6^kd^					
RHOA	42	36	51	30	Transforming protein RhoA
RHOC	36	34	45	29	Rho-related GTP-binding protein RhoC
RASH	33	22	25	14	GTPase HRas
RASK	29	41	21	19	GTPase KRas
RHOB	22	17	21	17	Rho-related GTP-binding protein RhoB

E: Distinct recovery of apoptosis-related molecules					
name	significant protein matches				full name
	A818.4	A818.4-v6^kd^	Capan1	Capan1-v6^kd^	
downregulated					
ADAM10	436	113	369	157	**ADAM metallopeptidase domain 10**
ADAM9	11	2	2	4	**Disintegrin and MMP domain-cont. protein 9**
CXCR4	43	0	38	1	**C-X-C chemokine receptor type 4**
FYN	67	6	53	10	**Tyrosine-protein kinase Fyn**
LYN	34	0	22	10	**Tyrosine-protein kinase Lyn**
SCARB1	13	3	15	9	Scavenger receptor class B member 1
SRC	46	0	37	8	**Proto-oncogene tyrosine-protein kinase Src**
TMBIM1	18	1	16	4	**Transmembrane BAX inhibitor motif cont.1**
UBE2D3	73	10	59	5	**Ubiquitin-conjugating enzyme E2 variant 3**
upregulated					
HNRNPK	4	29	6	18	**Heterogeneous nuclear ribonucleoprotein K**
CD99	5	13	2	3	**CD99 antigen**
LGALS3BP	373	482	391	789	Galectin-3-binding protein

1 Down- or upregulated in A818.4-CD44v6^kd^ and Capan1-CD44v6^kd^: green, in A818.4-CD44v6^kd^: red, in Capan1-CD44v6^kd^: blue, opposingly regulated in A818.4-CD44v6^kd^ and Capan1-CD44v6^kd^: violet, not regulated: black, >2-fold differences: bold.

**Figure 2:**
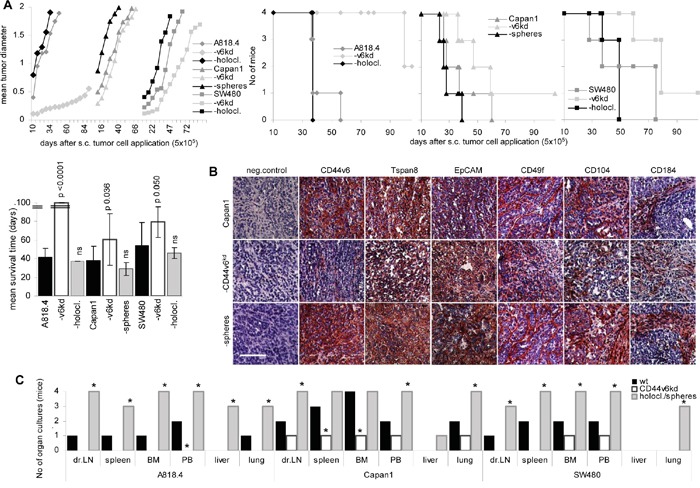
The impact of CD44v6 on tumor growth and progression Wt, CD44v6^kd^ and sphere/holoclone cells were s.c. injected. **A.** Tumor growth (mean values from 4 mice/group), survival time, survival rate and mean survival time are shown; significant differences to wt cells: *. **B.** Sections of shock frozen Capan1 tumors were stained with CD44v6, Tspan8, EpCAM, CD49f, CD104 and CD184 (scale bar: 100μm). **C.** At autopsy the indicated organs were collected and dispersed and maintained in culture for up to 4wk to control for the presence of tumor cells. Growth of CD44v6^kd^ cells is delayed. Spheres/holoclones are characterized by an accelerated start of tumor growth and recovery of tumor cells in hematopoietic organs, lung and liver.

To control for the *in vivo* relevance of CD44v6 on tumor progression, growth of wt, CD44v6^kd^ and spheres/holoclones was evaluated in SCID mice. Spheres/holoclones showed a growth advantage during the first 3wk after s.c. injection. Thereafter the growth rate resembled that of wt cells. Accordingly, though slightly reduced, the mean survival time did not significantly differ from that of wt tumor-bearing mice. Instead, growth of CD44v6^kd^ cells, particularly of A818.4-CD44v6^kd^ cells started with delay and the mean survival time of A818.4-CD44v6^kd^ bearing mice was significantly and that of Capan1- and SW480-CD44v6^kd^ bearing mice was borderline significantly prolonged (Figure 2A). Immunohistology confirmed maintenance of CIC markers in sphere or holoclone tumors, but no rescue in CD44v6^kd^ tumors (Figure 2B, Supplementary Figure 2). Though macroscopically metastases were not observed, tumor cells grew in peripheral blood and digested organ cultures established at autopsy. Tumor cells were recovered in the draining lymph node, the peripheral blood, bone marrow, spleen and lung of all or at least 3 mice that received spheres or holoclones, but only in 1 or 2 cultures from mice receiving wt cells. In the liver, tumor cells were only recovered from mice receiving spheres or holoclones. None or only 1 out of 4 mice receiving CD44v6^kd^ cells showed tumor cells in the dispersed organs (Figure 2C). These findings were confirmed for A818.4 and Capan1 wt, CD44v6^kd^ and holoclone / sphere cells in independent experiments.

Briefly, a CD44v6^kd^ affects anchorage-independent growth, sphere and holoclone formation, cell cycle progression, apoptosis resistance and expression of addition CIC marker, most pronounced of Tspan8. Though the underlying mechanism remains to be explored, this activity has to be kept in mind interpreting CD44v6^kd^-mediated effects. Tumor progression of CD44v6^kd^ cells is also impaired. The latter being the hallmark of CIC, we focused on the impact of CD44v6 on tumor cell migration and invasion, which are essential steps in the metastatic cascade. The impact of TEX, supposed to replace tumor cells, was controlled concomitantly.

### CD44v6 and tumor cell adhesion and migration

One of the genuine activities of CD44, though independent of variant isoform expression is adhesion to HA [14]. CD44 as well as Tspan8, binds additional matrix proteins via associated integrins, which initiates integrin activation and promotes motility.

A818.4, Capan1 and SW480 adhere to HA. While adhesion of spheres / holoclones is unchanged, that of CD44v6^kd^ clones is reduced (Supplementary Figure 3A). Reduced CD44v6^kd^ cell adhesion does not correlate with HAS3 expression, which was mitigated only in A818.4- and Capan1-CD44v6^kd^ cells. Hyal2 and Hyal3 were upregulated in A818.4- and Capan1-CD44v6^kd^ cells. Hyal1 expression was alike in all 3 CD44v6^kd^ lines (Supplementary Figure 3B, 3D) and HAS3 and Hyal2 did not colocalize with CD44v6 (Supplementary Figure 3C). Notably, HAS3, Hyal2 and Hyal3 expression was low in wt, CD44v6^kd^ and sphere/holoclone TEX. Instead, Hyal1 expression was high in wt and CD44v6^kd^ TEX, but was decreased in holoclone/sphere TEX (Supplementary Figure 3B). The higher Hyal2 and Hyal3 expression correlated in A818.4- and Capan1-, but not SW480-CD44v6^kd^ cells with a reduction in high molecular weight HA in the supernatant of these lines that might contribute to reduced adhesion (Supplementary Figure 3E). The finding argues against CD44v6 recruiting HAS and Hyal to TEX. Nonetheless, reduced HA adhesion could affect motility of CD44v6^kd^ cells.

Panther pathway analysis of a proteome analysis of wt and -CD44v6^kd^ A818.4 and Capan1 cells revealed 60 and 56, respectively, adhesion molecules in A818.4 and Capan1 cells, from which 22 and 18, respectively, were downregulated and 8 and 6, respectively, were upregulated in A818.4- and Capan1-v6^kd^ cells. Of special interest was the finding that from 14 tetraspanins and 5 integrins in A818.4 and Capan1 cells 11 and 9, respectively, tetraspanins and 3 of 5 integrins were downregulated (Supplementary Figure 4A, 4B, Table 1B). As Tspan8 is a CIC marker in PaCa and CoCa and is associated with integrins as well as engaged in TEX biogenesis [9, 48–50], we were particularly interested, whether CD44v6 regulates Tspan8 expression at the transcriptional or the post-transcriptional level. qRT-PCR analysis indicated that CD44v6 likely is engaged in Tspan8 transcription (Supplementary Figure 4C). Flow cytometry confirmed downregulation of CD49f and CD104 in cells and TEX of all 3 lines. Tspan8 expression also was strongly reduced in CD44v6^kd^ cells and TEX, whereas CD9 expression was upregulated in CD44v6^kd^ cells, but not TEX. Expression of tetraspanins and integrins in holoclones / spheres is similar to that in wt cells (Supplementary Figure 4D, 4E).

**Figure 3:**
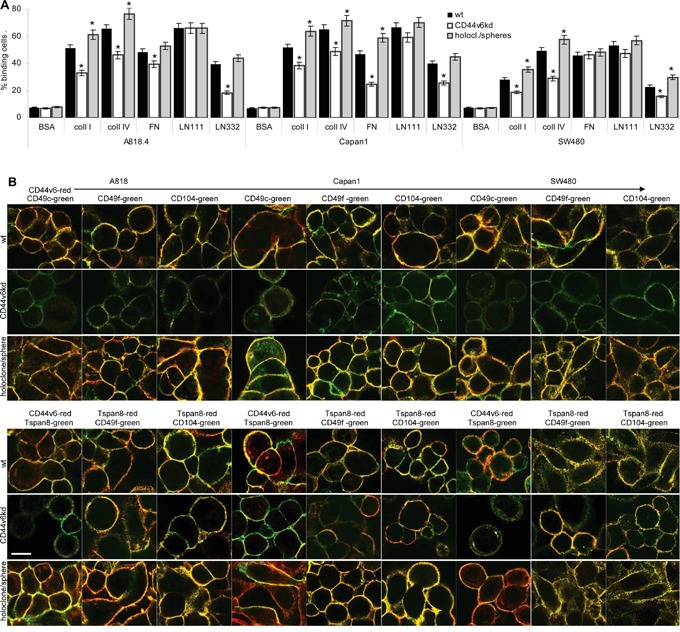
CD44v6 and matrix protein adhesion **A.** A818.4, Capan1 and SW480 wt, CD44v6^kd^ and holoclone/sphere cell were seeded on matrix protein-coated plates. After 2h at 37°C, 5%CO_2_, non-adherent cells were removed by vigorous washing. Adherent cells were stained with crystal violet and lysed measuring OD595nm (mean±SD of triplicates). The percent of adherent cells is shown; significant differences between wt and CD44v6^kd^ or sphere/holoclone cells: *. **B.** Colocalization of CD44v6 with Tspan8 and colocalization of CD44v6 and Tspan8 with integrins was evaluated by confocal microscopy in wt, CD44v6^kd^ and holoclone/sphere cells; overlays of green fluorescence (Tspan8) and red fluorescence (CD44v6) or green fluorescence (integrins) and red fluorescence (CD44v6 / Tspan8) are shown (scale bar: 10μm). Adhesion to coll I, coll IV and LN332 is strongly reduced in A818.4-, Capan1- and SW480-CD44v6^kd^ cells. Adhesion to LN111 is not affected and adhesion to FN mostly in Capan1-CD44v6^kd^ cells. CD44v6 and Tspan8 colocalize with CD49c, CD49f and CD104. Reduced adhesion of CD44v6^kd^ cells corresponds to reduced colocalization and pronounced colocalization of spheres/holoclones corresponds to stronger adhesion.

Adhesion to matrix proteins corresponded to CD44v6, Tspan8 and integrin expression. Binding to coll I, coll IV and FN, preferential integrin ligands, is stronger in A818.4 and Capan1 cells that express integrins at a higher level than SW480 cells. LN332 binding mostly promoted by tetraspanin-associated α6β4 is low in SW480 cells that express this integrin and Tspan8 at a low level. A CD44v6^kd^ was consistently accompanied by reduced adhesion to coll I, coll IV and LN332; binding to FN was slightly reduced only in A818.4- and Capan1-CD44v6^kd^ cells. Distinct to the largely unaltered expression (Supplementary Figure 4D), holoclones/spheres showed pronounced adhesion to coll I and IV and in SW480 cells to LN332 (Figure 3A).

Because of the variable impact of a CD44v6^kd^ on adhesion to matrix proteins, we speculated that colocalization of integrins with both CD44v6 and Tspan8 contributes to adhesion. Confocal microscopy showed colocalization of Tspan8 with CD44v6, which was maintained in CD44v6^kd^ cells, though barely visible due to the reduced CD44v6 and Tspan8 expression in CD44v6^kd^ cells. Furthermore the laminin binding integrins CD49c, CD49f and CD104 colocalized with CD44v6. Colocalization was pronounced in holoclones/spheres. But, distinct from wt cells, very low level CD44v6 in the kd lines does not colocalize with CD49c and poorly with CD49f and CD104. On the other hand, colocalization of CD49f and CD104 with Tspan8 is maintained (Figure 3B). We interpret the findings that adhesion is not exclusively promoted by CD44v6 and that downregulation of Tspan8 in CD44v6^kd^ cells contributes to loss of adhesiveness.

Taken together, the PaCa lines adhere more strongly to coll I, coll IV, FN and LN than to HA. The strong decrease in adhesion of CD44v6^kd^ cells and pronounced adhesion of spheres/holoclones relies in part on CD44v6 associating with integrins, particularly, Tspan8-associated integrins. A CD44v6^kd^ also affecting Tspan8 expression aggravates the impact of a CD44v6^kd^ on adhesion.

Integrin binding is accompanied by integrin activation and a shift towards motility promoting complexes [51]. As CD44v6- and Tspan8-associated integrins jointly promote adhesion, we proceeded evaluating the impact of CD44v6- and Tspan8-associated integrins on migration.

**Figure 4:**
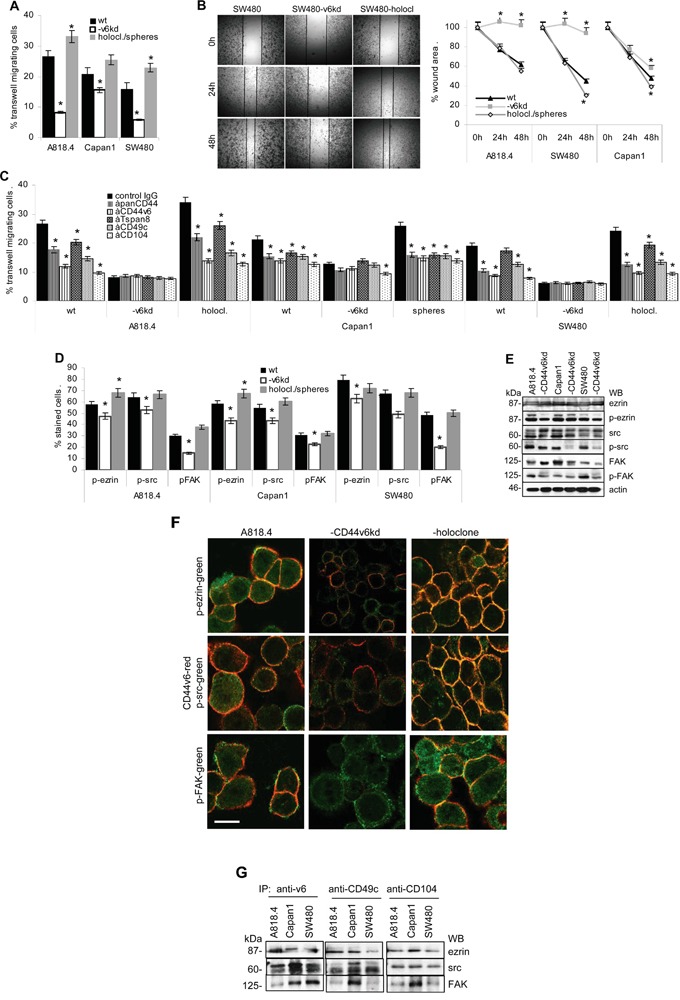
CD44v6 and migration **A.** A818.4, Capan1 and SW480 wt, CD44v6^kd^ and sphere/holoclone cells were suspended in serum-free medium and seeded in the upper chamber of transwell plates. The lower chamber contained medium with 20% FCS. Cells at the lower membrane site were stained with crystal violet after 16h. Cells were lysed and absorbance at OD595 was determined and is presented as percent of input cells (mean±SD of triplicates). **B.** Subconfluent monolayers of cells as in (A) were scratched with a pipette tip. Wound healing was recorded for 48h. A representative example of SW480 wt, CD44v6^kd^ and holoclone cells and mean±SD of the wound area in 3 independent assays are shown. **C.** Cells as above were incubated with the indicated antibodies in advance of being seeded in the upper part of a Boyden chamber. Transwell migration was evaluated as above. **D, E.** Cells as above were seeded overnight on LN332-coated plates; the percent of cells expressing p-ezrin, p-src and p-FAK were evaluated by flow cytometry (mean±SD of 3 independent assays) or WB. (A-D) Significant differences to wt cells: *. **F.** Colocalization of CD44v6 with p-ezrin, p-src and p-FAK in wt, CD44v6^kd^ and holoclone A818.4 cells was evaluated by confocal microscopy. Overlays of CD44v6 staining (red) and p-ezrin, -p-src and p-FAK (green) are shown (scale bar: 10μm); **G.** Immunoprecipitates of anti-CD44v6, -CD49c and -CD104 were separated by SDS-PAGE and blotted with anti-ezrin, -src and -FAK. Spheres/holoclones display significantly increased and CD44v6^kd^ cells significantly decreased migratory activity. Migration supporting activity relies on cooperativity of CD44v6 with integrins and joint CD44v6 - integrin signaling pathways.

Migration of wt and CD44v6^kd^ cells was evaluated by transwell migration and *in vitro* wound healing (scratch assay). Transwell migration of CD44v6^kd^ cells was significantly decreased, most strongly in A818.4- and SW480-CD44v6^kd^ cells and was strengthened in A818.4 and SW480 holoclones (Figure 4A). Corresponding findings accounted for wound closure (Figure 4B, Supplementary Figure 5A). To evaluate the contribution of CD44v6 versus integrins to motility, the experiment was repeated in the presence of blocking antibodies. In advance of transwell migration, cells were incubated with anti-panCD44, anti-CD44v6, anti-CD49c, anti-CD104 or anti-Tspan8. Wt and more pronounced holoclone / sphere, but not CD44v6^kd^ cell migration was inhibited by these antibodies, most strongly by anti-CD44v6 and anti-CD104 (Figure 4C), indicating a contribution of CD44v6 to integrin activation. Activated CD44 associates with the cytoskeleton via ERM proteins and activated integrins promote FAK phosphorylation, full FAK activation being initiated by recruited src [52]. WB and flow cytometry of PMA-stimulated cells revealed a significant reduction in ezrin, src and FAK phosphorylation in CD44v6^kd^ cells (Figure 4D, 4E). Confocal microscopy showed colocalization of p-ezrin, p-src and p-FAK with CD44v6 as well as CD49f and CD104 predominantly in holoclones and spheres. Colocalization of p-FAK with CD49f and CD104 was not sustainably affected in CD44v6^kd^ cells (Figure 4F, Supplementary Figure 5B). Immunoprecipitates confirmed the association of ezrin, src and FAK with CD44v6 as well as CD49c and CD104 (Figure 4G).

The findings confirm the contribution of CD44v6 to the shift of CD44v6- and Tspan8-associated integrins towards the motility promoting configuration.

### Cellular and TEX CD44v6 supports invasion

During the metastatic process, CIC are invading distant organs. CD44 is engaged in invasion by uPAR regulation and by promoting expression and activity of several MMPs [27]. Furthermore, premetastatic niche preparation suggests a contribution of TEX [31].

**Figure 5:**
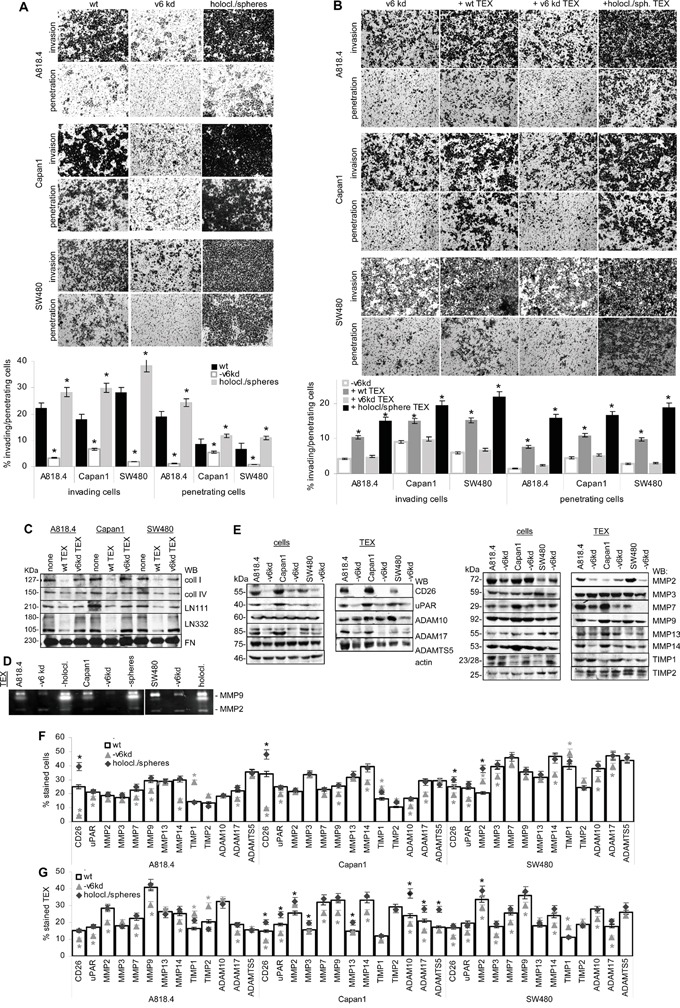
CD44v6, invasion and protease expression **A.** A818.4, Capan1 and SW480 wt, CD44v6^kd^ and sphere/holoclone cells were seeded on matrigel-coated inserts of transwell plates; **B.** A818.4-, Capan1- and SW480-CD44v6^kd^ cells were seeded on matrigel-coated inserts of transwell plates; where indicated, the matrigel contained wt, CD44v6^kd^ or sphere/holoclone TEX. (A, B) The number of matrigel invading and penetrating cells was evaluated by Giemsa staining, respectively, counting after 48h of culture. The mean percent±SD of invading and penetrating cells and representative examples are shown; (A) significant differences between wt, CD44v6^kd^ and sphere/holoclone cells: *; (B) significant differences between no TEX versus TEX: *. **C.** Matrix proteins were incubated for 24h with wt and CD44v6^kd^ TEX. TEX were removed by ultracentrifugation and recovery of matrix proteins was evaluated by WB. **D.** Zymography of TEX from wt, CD44v6^kd^ and sphere/holoclone TEX. **E.** Protease expression in A818.4, Capan1 and SW480 wt and CD44v6^kd^ cell and TEX lysates was evaluated by WB. **F, G.** Flow-cytometry analysis of proteases and protease inhibitors in wt, CD44v6kd and holoclone/sphere cells / TEX. The mean percent±SD (3 assays) of stained cells / TEX is shown; significant differences between wt, CD44v6^kd^ and sphere/holoclone cells / TEX: *. Invasiveness of CD44v6^kd^ cells is decreased and CIC-enriched cells display increased invasion. Loss of invasiveness of CD44v6^kd^ cells / TEX is accompanied by reduced protease expression, CD26, MMP14 and ADAM17 being consistently downregulated and TIMP1 being upregulated in CD44v6^kd^ cells. Only membrane-bound and CD44v6-associated protease expression correlates between cells and TEX. Wt and holoclone/sphere (not shown) TEX degrade matrix proteins, which allows CD44v6^kd^ cells to invade.

CD44v6 has a strong impact on matrigel invasion and penetration. Spheres/holoclones are more invasive and penetrating than wt cells (Figure 5A). Poorly invasive CD44v6^kd^ cells seeded on matrigel that contained wt or sphere/holoclone TEX regain invasive capacity, nearly reaching the efficacy of wt cells (Figure 5B), which suggested TEX degrading matrix proteins. Coculture of TEX with matrix proteins confirmed that A818.4, Capan1 and SW480 TEX degraded coll I, coll IV, LN111 and LN332. CD44v6^kd^ TEX from the three lines did not or poorly degrade matrix proteins (Figure 5C). Zymography showed strongly reduced MMP9 activity in CD44v6^kd^ TEX, but enhanced MMP2 and MMP9 activity in holoclone/sphere TEX (Figure 5D).

**Figure 6:**
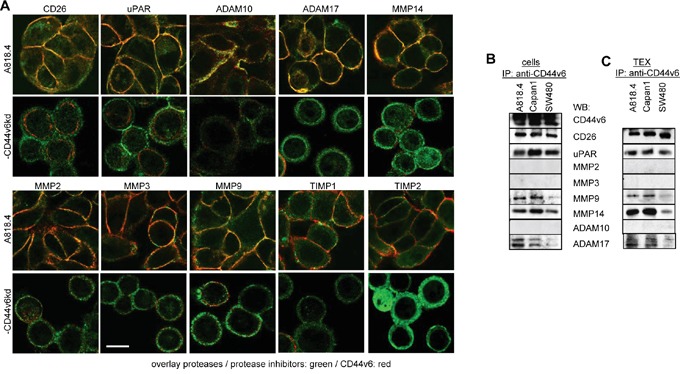
Protease cooperation with CD44v6 **A.** Colocalization of proteases / protease inhibitors (green) with CD44v6 (red) was evaluated in A818.4 wt and -CD44v6^kd^ cells by confocal microscopy; overlays of red (CD44v6) and green (proteases / protease inhibitors) fluorescence are shown (scale bar: 10μm). **B, C.** Coimmunoprecipitation of proteases with CD44v6 in A818.4, Capan1 and SW480 cells and TEX. CD44v6 colocalizes and coimmunoprecipitates with CD26, uPAR, MMP9, MMP14 and weakly with ADAM17. It weakly colocalizes, but does not coimmunoprecipitate with ADAM10, MMP2 and MMP3.

To consolidate a suggested linkage between CD44v6 and matrix degradation, protease expression was evaluated in wt, CD44v6^kd^ and holoclone/sphere cells and TEX. A proteome analysis revealed significantly reduced CD26, ADAM10, calpain5, calpain7, dipeptidase1 and MMP15 and upregulated TIMP1 and PAI1 expression in A818.4 and Capan1 CD44v6^kd^ cells (Table 1C). WB and flow-cytometry showed CD26, MMP14, ADAM17 and uPAR downregulation and TIMP1 upregulation in CD44v6^kd^ cells and in flow cytometry CD26 upregulation in spheres/holoclones. MMP13, ADAM10, ADAMTS5 and TIMP2 expression was not affected in CD44v6^kd^ cells (WB and flow-cytometry) or spheres/holoclones (flow-cytometry). The impact of a CD44v6^kd^ on MMP2, MMP3, MMP7 and MMP9 varied between the 3 lines (Figure 5E, 5F). Of special interest in concerning the activity of TEX was the comparison of protease recovery in TEX versus cells. CD26, MMP9, MMP14, uPAR and ADAM17 expression was similar in TEX and cells. Expression of MMP2, MMP7, MMP13 and TIMP1 differed between TEX and cells (WB and flow-cytometry). Upregulated protease expression was frequently seen in Capan1 sphere TEX, but rarely in A818.4 and SW480 holoclone TEX (flow-cytometry) (Figure 5E, 5G). The finding suggested that preferentially CD44v6 (or Tspan8)-associated proteases are transferred in TEX. Confocal microscopy showed pronounced colocalization of CD44v6 with CD26, uPAR, ADAM17, MMP14 and MMP2 (Figure 6A, Supplementary Figure 6). Coimmunoprecipitation confirmed an association of CD26, uPAR, ADAM17 and MMP14 with CD44v6; weak coimmunoprecipitation was seen with MMP9, particularly in A818.4 and Capan1 cells and TEX; MMP2 and MMP3 did not coimmunoprecipitate (Figure 6C).

The findings are summarized in Table 2B, which presents mean values of flow-cytometry and WB. This overview suggests that (i) CD44v6 can affect protease expression either at the transcriptional level or by stabilization via association; (ii) there is a link between CD44v6 downregulation and upregulation of protease inhibitors, which is independent of colocalization. It could proceed via CD44v6 transcriptional repression or via CD44v6-regulated miRNA; (iii) downregulated membrane-anchored proteases, which coimmunoprecipitate with CD44v6, are also downregulated in TEX. Without excluding an independent contribution of CD44v6, this finding points towards joint internalization of GEM-located molecules that after intracellular vesicle traffic are released in TEX. Regulation of protease expression via CD44v6 provides an explanation for reduced invasion of CD44v6^kd^ cells; CD44v6-dependent recovery of selected proteases in TEX can account for restoring invasion by holoclone/sphere TEX, which was confirmed *in vivo*.

**Table 2:** Overview on CD44v6 linked expression profiles

A: Expression levels of CD44v6, CIC markers and integrins													
Marker	Cell line	flow cytometry						WB				proteomic	
		cells			TEX			cells		TEX		cells	
		wt	v6kd	CIC	wt	v6kd	CIC	wt	v6kd	wt	v6kd	wt	v6kd
CD44v6	A818.4	+++	↓↓↓	↑	+++	↓↓↓	↑	+++	↓↓↓	+++	↓↓↓	+++*	↓↓↓*
	Capan1	+++	↓↓	↑	+++	↓↓	↑	+++	↓↓	+++	↓↓↓	+++*	↓*
	SW4 80	+++	↓↓↓	↑	+++	↓↓↓	↑	+++	↓↓↓	+++	↓↓↓		
MET	A818.4	++	↓	=	+	↓	=	++	=			+	↓↓↓
	Capan1	++	↓	=	++	(↓)	=	++	=			±	↓↓↓
	SW480	+	↓	=	+	↓	=	+	=				
EpCAM	A818.4	+++	=	=	+++	=	=	+++	=	++	=	+++	=
	Capan1	+++	=	=	+++	=	=	+++	=	++	=	+++	=
	SW480	+++	=	=	+++	=	=	+++	=	+++	=		
CD184	A818.4	+	↓↓	↑	+	↓	(↑)					+	↓↓↓
	Capan1	+	↓↓	↑	+	↓	(↑)					+	↓↓↓
	SW480	+	↓↓	=	+	↓	=						
Tspan8	A818.4	+++	↓↓	=	+++	(↓)	=	+++	↓↓	+++	=	++	↓↓↓
	Capan1	+++	↓	=	++	=	=	+++	↓	+++	=	++	↓↓
	SW480	+	(↓)	↑↑	+++	(↓)	=	+	↓	++	=		
CD104	A818.4	++	↓↓	=	++	↓	=	++	↓			+++	↓
	Capan1	+++	↓	=	++	(↓)	=	++	↓			+++	=
	SW480	+++	↓	(↑)	++	(↓)	=	+++	↓↓				
CD49f	A818.4	+++	↓	=	+++	↓	=					+++	=
	Capan1	+++	↓	=	+++	↓	=					+++	=
	SW480	+++	↓	=	+++	↓	=						
CD29	A818.4	+++	↓↓	=	+	↓	=					+++	=
	Capan1	++	=	=	+	(↓)	=					+++	=
	SW480	+++	(↓)	=	++	(↓)	=						
CD49c	A818.4	++	↓↓	=	++	↓	=					++	↓
	Capan1	++	=	=	+	=	=					++	=
	SW480	++	↓↓	=	++	=	=						

B: CD44v6-related protease expression										
Marker	Cell line	CD44v6 kd expression level				CD44v6 association				
		cells		TEX		confocal			co-IP	
		FI	WB	FI	WB	wt	v6kd	CIC	cells	TEX
CD26	A818.4	↓↓	↓↓	↓↓	↓↓↓	++	↓↓	↑	++	++
	Capan1	↓↓	↓	↓↓	↓↓↓	++	↓↓	↑	++	++
	SW480	↓	↓↓	↓↓	↓↓↓	++	↓↓	↑	++	++
uPAR	A818.4	↓	↓	↓↓	↓↓	++	↓↓	↑	+++	++
	Capan1	↓	↓	↓↓	↓	++	↓↓	↑	+++	++
	SW480	↓	↓	↓	↓	++	↓↓	↑	+++	++
ADAM10	A818.4	=	=	=	=	±	↓	=	--	--
	Capan1	↓	=	↓	(↓)	±	=	=	--	--
	SW480	↓	=	↓	↓	±	=	=	--	--
ADAM17	A818.4	↓	↓↓	↓	↓	++	↓↓	=	++	+
	Capan1	↓	↓↓	↓↓	↓↓	+	↓	=	+	+
	SW480	↓	↓↓	↓	↓	+	↓↓	=	±	±
ADAMTS5	A818.4	=	=	=	(↓)	nt^1^	nt	nt	nt	nt
	Capan1	=	=	=	(↓)	nt	nt	nt	nt	nt
	SW480	=	=	=	=	nt	nt	nt	nt	nt
MMP14	A818.4	↓↓	↓	↓↓	↓↓	++	↓↓	=	++	++
	Capan1	↓	↓	↓	↓	++	↓↓	=	+++	+++
	SW480	↓	↓	↓	(↓)	++	↓↓	=	+	+
MMP2	A818.4	=	↓	↓↓	↓↓	+	↓	↑	--	--
	Capan1	=	=	↑	↑	+	=	↑	--	--
	SW480	↑	↑	↓	↓↓	++	↓↓	=	--	--
MMP3	A818.4	=	=	=	=	+	↓↓	=	--	--
	Capan1	=	=	=	=	+	↓↓	↑	--	--
	SW480	↓	↓	↓	↓	+	↓↓	=	--	--
MMP7	A818.4	↓	↓	↓	↓	+	↓	=	nt	nt
	Capan1	=	↓	↓↓	↓↓	±	↓	=	nt	nt
	SW480	=	=	↓↓	↓↓↓	±	↓	=	nt	nt
MMP9	A818.4	↓	↓	↓	(↓)	++	↓↓	=	+	+
	Capan1	↓	(↓)	↓	↓	++	↓↓	=	+	+
	SW480	=	=	↓	↓	+	↓	=	±	±
MMP13	A818.4	=	=	↓	↓	nt	nt	nt	--	--
	Capan1	=	=	↓	↓	nt	nt	nt	--	--
	SW480	=	=	=	=	nt	nt	nt	--	--
TIMP1	A818.4	↑↑	↑↑↑	↑	↑	+	↓↓	=	nt	nt
	Capan1	↑↑	↑↑	=	↓	+	↓	=	nt	nt
	SW480	↑↑	↑↑	↑	↑	+	↓↓	=	nt	nt
TIMP2	A818.4	=	=	↑	↑	±	↓↓	=	nt	nt
	Capan1	=	=	=	=	±	(↓)	=	nt	nt
	SW480	=	=	=	=	+	=	=	nt	nt

1 nt: not tested

### CD44v6 contributes to TEX binding

**Figure 7:**
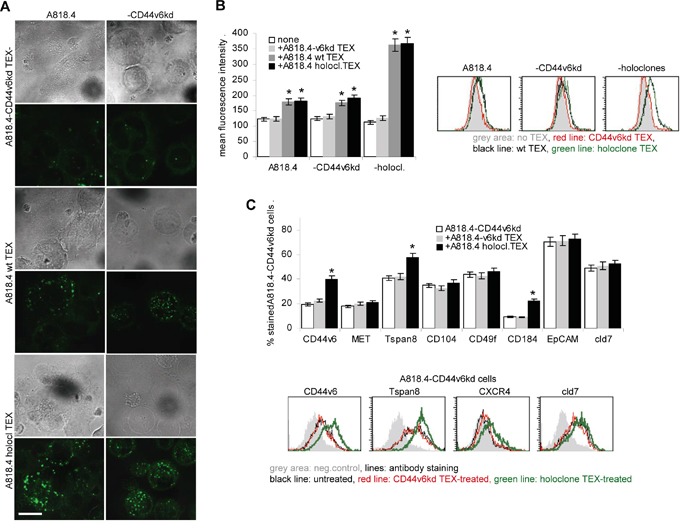
The impact of CD44v6 on TEX uptake **A-C.** A818.4^wt^, CD44v6^kd^ and holoclone TEX were Dio_18_(3)- or DHPE-labeled and incubated with A818.4-CD44v^kd^ or wt or holoclone cells: (A) Dio_18_(3)-labeled TEX were incubated overnight with A818.4 wt and -CD44v6^kd^ cells. Brightfield and overlays of green fluorescence with brightfield (confocal microscopy are shown (scale bar: 10μm). (B) DHPE-labeled TEX were incubated overnight with A818.4 wt, -CD44v6^kd^ and holoclone cells. TEX uptake was evaluated by flow cytometry. Mean fluorescence intensity±SD (3 assays) and representative examples are shown. A significant increase in the fluorescence intensity is indicated by *. (C) Flow cytometry analysis of CIC marker expression in A181.4-CD44v6^kd^ cells after 48h coculture with TEX. Representative examples and the mean percent±SD (3 assays) of stained cells are shown; significant differences between A818.4-CD44v6^kd^ cells cultured in the absence or presence of holoclone TEX: *. A818.4^wt^ and holoclone, but not A818.4-CD44v6^kd^ TEX are readily taken up by wt, CD44v6^kd^ and holoclone cells. Holoclone TEX-treated A818.4-CD44v6^kd^ cells upregulate CD44v6, Tspan8 and CXCR4 expression.

TEX binding and uptake being a prerequisite for target cell modulation, we first evaluated the uptake of dye-labeled TEX by wt, CD44v6^kd^ and holoclone A818.4 cells. Confocal microscopy and flow-cytometry revealed that CD44v6 competent, but not CD44v6^kd^ TEX are readily taken up by wt, CD44v6^kd^ and holoclone A818.4 cells (Figure 7A, 7B). After prolonged coculture for 48h, holoclone TEX uptake by CD44v6^kd^ cells was accompanied by upregulated CIC marker CD44v6, Tspan8 and CXCR4 expression. Expression of other CIC markers was not significantly affected (Figure 7C).

The striking loss of CD44v6^kd^ TEX uptake was unexpected. It might be due to the severe distortion of GEM complexes. Pronounced holoclone TEX uptake-induced upregulation of selected markers provides first evidence towards non-CIC modulation by TEX.

### CD44v6 TEX transfer migration and invasion supporting features in CD44v6^kd^ cells

Wt and particularly sphere / holoclone TEX sufficed to re-establish A818.4- and Capan1-CD44v6^kd^ cell motility in transwell migration (Supplementary Figure 7A). During wound healing on LN332-coated plates, the migration promoting activity of A818.4-, Capan1- and SW480-sphere/holoclone TEX became significant after 40h of coculture and was most pronounced after 52h (Supplementary Figure 7B, 7C). As demonstrated for A818.4-CD44v6^kd^ cells, there was no evidence for upregulated integrin expression (Figure 7C and data not shown). But, low level E-cadherin expression became largely abolished, whereas N-cadherin and vimentin expression increased, although to a minor degree. The same accounted for MMP2 and, less pronounced, MMP9 expression (Supplementary Figure 7D).

**Figure 8:**
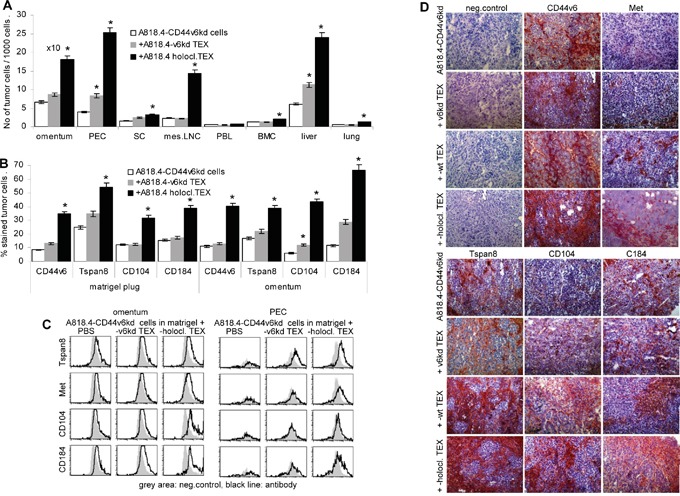
Holoclone TEX support regain of A818.4-CD44v6kd cell motility *in vivo* A818.4-CD44v6^kd^ cells (1×10^6^) were embedded in matrigel and were i.p. injected. Mice received 100μg A818.4-CD66v6^kd^ or holoclone TEX (i.p.) 2x/wk and were sacrificed after 3wk. Organs were excised and shock frozen or dispersed. **A.** Recovery of tumor cells in omentum, liver, lung and hematopoietic organs was evaluated by flow cytometry gating the tumor cell population according to size and granularity. The number of tumor cells / 1000 cells (mean±SD of 5 mice) is shown. **B, C.** Flow cytometry analysis of gated tumor cells (matrigel plug, omentum, PEC) stained with the indicated markers. The % stained tumor cells (mean±SD of organs of 5 mice) and/or representative examples and tare shown. **D.** Immunohistochemistry of sections of shock frozen matrigel plugs stained with the indicated CIC markers. Holoclone TEX facilitate emigration of poorly migrating CD44v6^kd^ cells from the matrigel plug, which is accompanied by a strong upregulation of CIC markers including CD104, which was not seen after *in vitro* coculture.

To control for the contribution of CD44v6 to the *in vivo* transfer of migratory and invasive potential via CIC TEX, matrigel-embedded poorly migrating and non-invasive A818.4-CD44v6^kd^ cells were i.p. injected. Where indicated, mice received concomitantly 100μg holoclone TEX, i.p., repeating the injection twice per week. Mice were sacrificed after 3 weeks and the matrigel plug, the omentum, the peritoneal cavity cells, liver, lung, peritoneal lymph nodes, spleen, bone marrow and peripheral blood were collected and solid organ cells were dispersed. The number of tumor cells was evaluated by flow cytometry gating the population of large and more granulated tumor cells. A818.4-CD44v6^kd^ cells were recovered in the matrigel plug and the adjacent omentum. Few tumor cells were recovered in the peritoneal cavity and the liver. Tumor cells were not or very rarely recovered in spleen, mesenteric lymph nodes, bone marrow, peripheral blood and lung. Whereas, when mice received A818.4 holoclone TEX, i.p., a significantly higher number of A818.4-CD44v6^kd^ cells were recovered in omentum, peritoneal cavity, mesenteric lymph nodes and liver. Few tumor cells were also recovered in spleen, peripheral blood, bone marrow and lung (Figure 8A). Notably, flow cytometry of the gated tumor cell population and immunohistochemistry of the matrigel plug showed partial regain of CD44v6 expression and upregulated Tspan8, α6β4 and CXR4 expression in A818.4-CD44v6^kd^ cells of holoclone TEX-treated mice (Figure 8B-8D).

**Figure 9:**
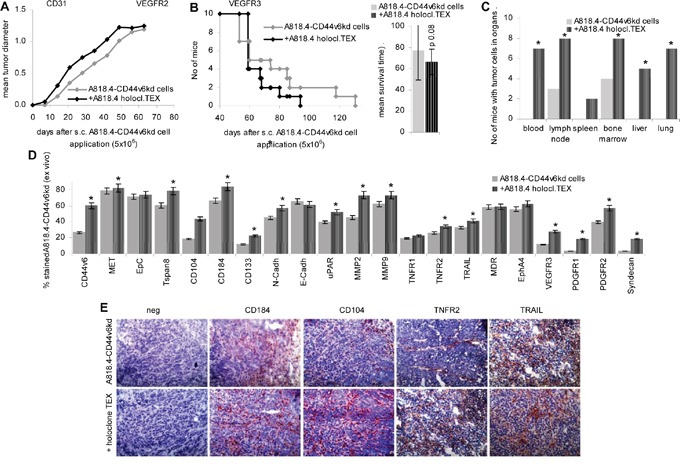
Holoclone TEX rescue metastatic features in A818.4-CD44v6^kd^ cells A818.4-CD44v6^kd^ cells (5×10^6^) were s.c. injected. Mice received 100μl NaCl or 100μg A818.4 holoclone TEX (i.v.) 2x / week. **A.** Tumor growth and **B.** survival time are recorded. **C.** At autopsy, potential metastatic organs were collected, dispersed and cultured to survey tumor cell outgrowth. The number of organs, where tumor growth was observed is shown. **D.** Flow-cytometry of dispersed tumor cells stained for CIC and EMT markers, several proteases, apoptosis receptors, RTKs and syndecan. **E.** Immunohistology of tumor tissue evaluating expression of the CIC markers CD184 and CD104 and of TNFR2 and TRAIL. CIC TEX promote metastatic features in A818.4-CD44v6^kd^ cells with upregulation of CIC, EMT markers, proteases and mostly (lymph)angiogenesis related receptors.

Finally, we controlled whether a systemic (i.v.) application of holoclone TEX suffices to affect local (s.c.) tumor growth. I.v. application of A818.4 holoclone TEX sufficed for an accelerated start of s.c. A818.4-CD44v6^kd^ cell growth (Figure 9A). Though the mean survival time was not significantly prolonged (Figure 9B), dispersed organ cultures of sacrificed mice showed recovery of A818.4-CD44v6^kd^ cells in the PB, in LN, BM, liver and lung of holoclone TEX-treated mice (Figure 9C). Flow-cytometry of the dispersed freshly harvested tumors and/or immunohistochemistry of shock frozen tumors confirmed upregulated expression of the CIC- and EMT markers CD44v6, Tspan8, CD104, CD184, CD133 and N-cadherin. We did not observe downregulation of E-cadherin (Figure 9D, 9E). In addition, and similar to the vitro cocultures uPAR, MMP2 and MMP9 expression became upregulated by CIC TEX treatment. There was no evidence for major changes in apoptosis receptor and MDR (multidrug resistance gene)1 expression. Though not excluding an impact of CIC TEX on apoptosis resistance, the finding is in line with the comparative proteome analysis of A818.4 and Capan1 wt versus -CD44v6^kd^ cells, which did not provide evidence for a major impact of CD44v6 on apoptosis-related molecules (Supplementary Figure 8A). Holoclone TEX also induced upregulated VEGFR3, PDGFR1 and PDGFR2 expression and de novo expression of syndecan1 (Figure 9D, 9E). Proteome analysis indicated no significant changes in the percent of angiogenesis pathway engaged molecules in A818.4-CD44v6^kd^ cells, in Capan1-CD44v6^kd^ cells the percent of angiogenesis pathway engaged molecules was reduced. However, in both CD44v6^kd^ lines there was a surprising dysregulation of ephrins, ephrin receptors and ephrin receptor linked molecules (Supplementary Figure 8B, Table 1D). Flow-cytometry after coculture of A818.4-CD44v6^kd^ cells with holoclone TEX revealed upregulation of low level VEGFR3 and Lyve expression, which corresponded to the *ex vivo* findings. Yet, high EphA4 expression in A818.4-CD44v6^kd^ cells was not significantly altered (Supplementary Figure 8C).

Taken together, holoclone TEX suffice to induce motility and invasiveness in A818.4-CD44v6^kd^ cells *in vitro* and *in vivo*, which is accompanied by a partial regain of CIC and EMT marker expression, upregulation of protease expression and receptor tyrosine kinases that in concert may well account for the regain of metastatic features.

### The tumor progression-promoting impact of holoclone TEX on host cells

There is ample evidence that TEX affect the host. This was most intensely studied in the context of the establishment of a premetastatic niche and for tumor-induced immunosuppression [43, 53]. We reported on both features in a syngeneic rat pancreatic cancer model that took the particular role of CD44v6 into account [20, 54]. Though we here focused on CIC-enriched TEX and their impact on non-CIC / non-metastasizing tumor cells, the strong impact of CIC TEX on host cells requires to be briefly mentioned.

**Figure 10:**
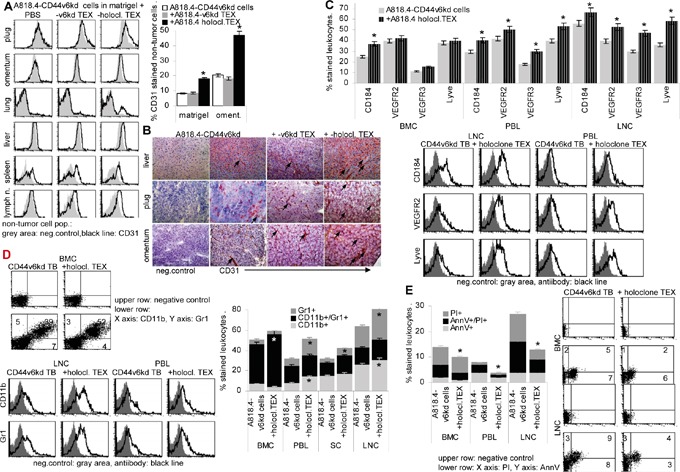
The impact of holoclone TEX on host cells A818.4-CD44v6^kd^ cells (5×10^6^) were embedded in matrigel and injected i.p. or were s.c. injected. Mice received 100μl NaCl or 100μg A818.4 holoclone TEX (i.p. or i.v.) 2x / week. **A.** Flow cytometry analysis of CD31^+^ cells in the matrigel plug, omentum, lung, liver, spleen and mesenteric lymph nodes, gating the non-tumor cells. The mean % stained non-tumor cells±SD (organs of 5 mice) and representative examples are shown. **B.** Immunohistochemistry of liver, plug and omentum stained with anti-CD31, staining of selected vessels is indicated by an arrow. **C-E.** Flow-cytometry analysis of (C) angiogenic factor receptor expression in BMC, PBL and LNC, (D) myeloid cells including myeloid-derived suppressor cells (CD11b^+^Gr1^+^) and (E) apoptotic cells in hematopoietic organs. (A, C-D) Mean values±SD of cells from 3-5 mice and representative examples are shown. Significant differences depending on holoclone TEX treatment are indicated: *. A818.4 holoclone TEX modulate host cells. This is demonstrated for (lymph)angiogenesis, myeloid and MDSC expansion and leukocyte apoptosis resistance.

A first hint towards holoclone TEX supporting angiogenesis was derived from the spread of matrigel-embedded tumor cells in distant organs. Indeed, angiogenesis was strongly increased in mice receiving A818.4 holoclone TEX. This is shown by flow-cytometry after staining with CD31 (endothelial cell adhesion molecule 1) in the matrigel plug, omentum, lung, liver, spleen and lymph nodes gating the non-tumor cells and in liver, plug and omentum by immunohistochemistry. To note, upregulated angiogenesis was also seen in lung, liver, spleen and mesenteric lymph nodes, where tumor cells were not or rarely recovered (Figure 10A, 10B). Strong angiogenesis was also seen after i.v. injection of holoclone TEX (data not shown). There is evidence for an engagement of hematopoietic cells and/or progenitor cells residing in hematopoietic / lymphatic organs, holoclone TEX promoting upregulation of CXCR4 in BM, PB and LN and higher levels of VEGFR2, VEGFR3 and Lyve in PB and LN (Figure 10C). Furthermore, myeloid cells (CD11b^+^) were upregulated in LN, spleen, PB and BM. A strong increase in myeloid-derived suppressor cells (MDSC; CD11b^+^/Gr1^+^) was only observed in BMC (Figure 10D). Finally, flow-cytometry of freshly harvested leukocytes from tumor-bearing mice indicated that holoclone TEX supported leukocyte survival, though to a minor degree (Figure 10E).

Thus, TEX promote (lymph)angiogenesis even in the xenogeneic host, drive myelopoiesis towards MDSC and strengthen leukocyte apoptosis resistance. As these features are observed in the xenogeneic host, it is likely that holoclone TEX communicate mostly with early progenitor cells.

Taken together, the metastogen and CIC marker CD44v6 contributes to migration and invasion. The high activation of CD44v6-associated integrins and proteases in spheres/holoclones is severely impaired in CD44v6^kd^ cells. CD44v6-promoted changes in integrin, protease and signaling molecule expression are maintained in TEX, the transfer of CIC-TEX into CD44v6^kd^ cells and host cells supporting metastasis.

## DISCUSSION

CD44v6 is a metastogen and a CIC marker [27]. It associates and cooperates with RTK, particularly MET that supports metastasis [17]. CD44v6 is also engaged in apoptosis resistance that can proceed via several pathways [55]. The cytoplasmic CD44v6 tail contributes to epithelial-mesenchymal transition (EMT) gene transcription [56]. Protease regulation by CD44 or CD44v6 can promote invasiveness [57] and CD44v6 expression correlates with poor prognosis in gastrointestinal cancer [58]. Based on these findings, we explored the contribution of cellular and TEX CD44v6 on migration and invasion and discuss the transfer of migrating and invasive capacity of CD44v6^+^ CIC TEX into non-CIC.

### Characterization of PaCa and CoCa CIC and TEX

CD44v6 was stably kd or was enriched by holoclone (A818.4, SW480) or spheroid (Capan1) growth in two PaCa and one CoCa lines. The CD44v6^kd^ was accompanied by reduced anchorage-independent growth, loss in the capacity to form holoclones or spheres, a significant decrease in drug resistance, tumor growth retardation and a strong reduction in migrating / metastasizing tumor cells. On the other hand, CD44v6 expression was increased in holoclones/spheres, cell cycle progression was retarded, drug resistance was increased and migrating / metastasizing tumor cells were recovered in draining lymph nodes, spleen, bone marrow, peripheral blood and lung. Only holoclones/spheres settled in the liver. These features correspond to our and several other groups' work [4, 9, 30, 31].

Unexpectedly, reduced CD44v6 expression was accompanied by reduced expression of several CIC markers. While reduced MET expression in CD44v6^kd^ cells is in line with CD44v6 regulating MET transcription [17], we were not aware of a linkage between downregulation of CD184, α6β4, CD133, cld7 and, most pronouncedly Tspan8 expression [4, 5, 9, 59, 60] in CD44v6^kd^ cells. One possible explanation could rely on the powerful cotranscription factor activity of CD44-ICD [61], being engaged in EMT by regulating Wnt genes, Snail-1 transcription and Nanog processing [62–64] and also affecting miRNA transcription or silencing [55], whereby transcription or translation could become regulated. We provide first evidence of at least some contribution of CD44v6 in Tspan8 transcription. We will further explore this question, as Tspan8 downregulation has far reaching consequences for TEX.

Tetraspanins are organized in GEM, where they associate with a multitude of molecules, including CD44v6 and preferentially laminin-binding integrins [49, 65, 66]. Taking into account that prominin-1 and (palmitoylated) cld7 are also GEM-located [67, 68], transcriptional or posttranscriptional regulation of Tspan8 via CD44 may account for the additional changes in CIC marker expression. Furthermore, tetrapanins play a central role in GEM invagination, early endosome transport and exosome release [49, 50, 69], which can explain the subtle changes in TEX composition in CD44v6^kd^ cells. It is noteworthy that according to our current state of knowledge the few CD44v6-dependent differences between cells versus TEX were restricted to molecules not associating with either CD44v6 or Tspan8. Whether the regulation of Tspan8 expression by CD44v6 is, indeed, the basic cause for the defective uptake of CD44v6^kd^ TEX by wt, CD44v6^kd^ and holoclone cells awaits further exploration. However, without question the downregulation of Tspan8 expression in CD44v6^kd^ cells has far reaching consequences on TEX, which miss or display reduced levels of the whole panel of GEM-located and/or Tspan8-associated molecules including CIC markers.

### Cellular and TEX CD44v6 and PaCa / CoCa adhesion, migration and invasion

CD44 is the major HA receptor [14]. Though CD44v6 does not contribute to HA binding, binding is reduced in CD44v6^kd^ cells. This likely is a sequel of the overall reduction in CD44 expression, as there is no evidence for a strong reduction in HAS3 expression in the CD44v6^kd^ cells. Also, Hyal2 and Hyal3 expression, though increased in CD44v6^kd^ cells, was not sufficient to induce pronounced HA degradation, which could hamper cell adhesion [70]. Notably, except for reduced recovery of Hyal1 in holoclones/spheres, HAS3, Hyal2 and Hyal3 recovery in TEX was not affected, arguing against HA binding contributing to the CIC feature of CD44v6.

Coll, FN and LN binding was distinctly affected in CD44v6^kd^ and sphere/holoclone cells. Anti-CD44v6, anti-α6 and anti-β4 strongly inhibiting adhesion pointed towards joint signaling pathways. Furthermore, migration of CD44v6^kd^ cells was reduced and migration of spheres and holoclones was accelerated. When cells were cultured on LN332, ezrin, FAK and src phosphorylation became upregulated in wt and sphere/holoclone, but not CD44v6^kd^ cells. This is in line with CD44v6 associating with integrins either directly or via Tspan8 [49, 71]. Furthermore, CD44 and integrins mostly use common signaling pathways, like activated src phosphorylating GEFs, which regulate Rac1 or ROK via RhoA [72]. Through the association with integrins, CD44 also gets access to FAK and the CD44-integrin-motility complex moves towards the leading edge of the cell [73]. Coimmunoprecipitation confirmed the association of integrins with CD44v6 and Tspan8 and the mutual access to downstream signaling.

Taken together, the strong impact of CD44v6 on migration relies on the association with integrins, directly or via associated Tspan8. Due to proximity upon integrin ligand binding, CD44 and integrin signaling pathways become concomitantly activated, explaining the high motility of CIC-enriched spheres/holoclones.

Migrating tumor cells need to invade, settle and grow in distant organs. CD44v6 can contribute to invasion by collaborating with TNF that promotes MMP9 transcription [74], but also by affecting uPAR transcription [20] and MMP14 expression [75]. Via associating with MMP14, CD44v6 recruits MMP2 and MMP9 to the plasma membrane, where they become activated and are protected from degradation [34].

Proteome analysis revealed pronounced reduction of several proteases and upregulation of PAI1 and TIMP1. WB and flow-cytometry uncovered, in addition, reduced uPAR and ADAM17 expression in CD44v6^kd^ cells and TEX. We interpret these findings that regulation of proteases via CD44v6 is a multilevel process, where association of proteases with CD44, CD44-promoted transcription or CD44-initiated activation of protease transcription factors may cooperate. Cleavage of CD44 by proteases, which supports CD44-ICD generation [76, 77] adds an additional factor to the CD44-protease interplay [33]. Proteases associated with CD44v6 were recovered in TEX and, as described before [78, 79], TEX proteases are function competent. Expression particularly of MMP9 is significantly reduced in CD44v6^kd^ TEX such that CD44v6^kd^ TEX did not or poorly degrade matrix proteins. Expectedly, lost invasive capacity of CD44v6^kd^ cells was restored *in vitro* and *in vivo*, when the matrix was exposed to sphere/holoclone TEX. Proteases not being consistently upregulated in spheres and holoclones suggests that invasion is not a central CIC feature. Nonetheless, CD44v6 supporting protease transcription and/or recruitment of soluble MMPs via MMP14 and protease recovery in CD44v6-competent TEX fosters the metastatic process, which was most convincingly demonstrated by the dissemination of matrigel-embedded CD44v6^kd^ cells, when assisted by holoclone TEX.

Briefly, CD44v6 collaborates with proteases, most of which also associate with Tspan8 [75, 78, 80]. Low TIMP1 and PAI1 expression in CD44v6^kd^ cells deserves further elaboration. Low PAI1 [81] and TIMP1 [82] expression can contribute to loss of invasion. However, TIMP1 may also promote metastases by preserving MET expression and PI3K/Akt activation and promoting HIF1α and miR-210 upregulation, which both require cooperation with the tetraspanin CD63 [83]. Thus, the CD44v6-protease liaison contributes to the metastatic process and TEX efficiently fulfill this task.

### Outlook and conclusion

Several questions arising during this study have not yet been fully answered. (i) Importantly, CIC TEX can transfer motility to non-CIC. The delayed impact of sphere/holoclone TEX on wound healing after 40h of coculture argues for TEX uptake and reprogramming of non-CIC. The strongest effect was seen on CXCR4 upregulation, but CD104, N-cadherin and vimentin expression also became upregulated, pronounced N-cadherin and vimentin expression being associated with the EMT phenotype (84). Upregulated expression of MMP2 and MMP9 may additionally support invasiveness of the motile tumor cells. (ii) CD44v6 is engaged in apoptosis resistance, frequently via activation of antiapoptotic pathways or by stabilizing drug resistance [27]. There was no evidence for upregulation of drug resistance genes and TNFR2 was only slightly upregulated. CD44 is also engaged in HIPPO signaling, where Yap overexpression inhibits apoptosis by upregulation of cyclinE and cIAP1/2 [85]. However, Yap expression was not altered after coculture of CD44v6^kd^ cells with holoclone TEX (data not shown). On the other hand, holoclone TEX promoted leukocyte apoptosis resistance. Thus, an impact of holoclone TEX on A818.4-CD44v6^kd^ drug resistance is likely, but the pathway remains to be unravelled. We speculate on a possible engagement of Eph / Eph receptors [86] that expression is distorted in A818.4-CD44v6^kd^ cells. Not being aware of a described linkage between CD44v6 and ephrin / -receptors, the topic remains to be explored. A possible engagement of PDGFR2, which promotes growth, survival and migration [87] and shows strongly upregulated expression in holoclone-treated CD44v6^kd^ tumor bearing mice also remains to be explored. Finally, upregulated syndecan1 expression could contribute to apoptosis resistance via its cooperation with integrins [88]. (iii) TEX of a xenogeneic tumor will not cover the whole array of possible interactions with host cells. Nonetheless, a linkage of CD44v6 to angiogenesis was suggested by pronounced (lymph)angiogenesis of A818.4-CD44v6^kd^ tumors in mice receiving holoclone TEX. Furthermore, VEGFR3 and Lyve expression, both associated with lymphangiogenesis [89–91] was stimulated in A818.4-CD44v6^kd^ cells cocultured with holoclone TEX as well as in vessel endothelium in holoclone TEX-treated mice, which indicated holoclone TEX also affecting host cells. Notably, a slight upregulation of VEGFR3 and Lyve expression was also seen in A818.4-CD44v6^kd^ cells cocultured with holoclone TEX. The increase in MDSC in the BM is another example of holoclone TEX reprogramming besides non-CIC also host cells towards facilitating the metastatic cascade [92]. Whether holoclone TEX fulfill this large array of activities, motility, apoptosis resistance, angiogenesis and creating an immunosuppressive milieu by target cell activation including initiation of gene transcription and/or by silencing via miRNA, has not yet been answered. Both mechanisms were repeatedly described [93–96]. Based on the clusters of RTK and signaling molecules in TEX [39, 40, 97, 98], we favor a major, though not exclusive role of autonomous target modulation initiated by TEX binding. There remains the question on the upregulated CD44v6 expression in holoclone TEX-treated CD44v6^kd^ cells. Transfer of TEX CD44v6 could be one possibility. Alternatively, transcription of CD44/CD44v6 may become strongly promoted outsmarting the shRNA or antiviral responses may become stimulated.

The downregulation of additional CIC markers in CD44v6^kd^ cells was the most notable and unexpected finding. Our data point towards Tspan8 being the prime target, which may regulate additional Tspan8-associated and/or GEM-located CIC markers. Tspan8 is a constitutive exosome marker, is engaged in exosome biogenesis as well as in exosome target binding [50, 69, 98]. There is evidence for an engagement of CD44v6 in Tspan8 transcription. Several CIC features being transferred via TEX into non-CIC, clarifying the CD44v6 engagement in Tspan8 expression becomes most urgent.

We confirmed by a CD44v6^kd^ and CD44v6-enriched CIC the contribution of CD44v6 to motility, invasion, anchorage-independent growth and apoptosis resistance. The selective engagement of CD44v6 was supported by the finding that from >400 proteins distinctly regulated in A181.4- or Capan1-CD44v6^kd^ cells only 14 were differently regulated in the two PaCa lines (Supplementary Table 1). Importantly, CD44v6 affects additional CIC marker expression and several CD44v6 activities are transferred via CIC TEX into non-CIC. This central role of CD44v6 holds great promise for a CIC collapse by interfering with CD44v6 expression.

## MATERIALS AND METHODS

### Tumor lines

The PaCa lines A818.4 [99], Capan1 [100] and the CoCa line SW480 [101] were transfected with CD44v6 shRNA (Qiagen, Hilden, Germany) (Supplementary Table 2) using the pSuper-retro.neo vector. Stable CD44v6^kd^ lines were established by cloning. Cells were maintained in RPMI1640/10%FCS w/wo 0.5μg/ml G418.

### Antibodies and chemicals

Supplementary Table 3.

### Tissue preparation and cell isolation

Mice were sacrificed by cervical dislocation. Single cell suspensions of draining lymph nodes, spleen, liver and lung were prepared by pressing through fine gauze. Bone marrow cells (BMC) from femora and tibiae and PEC were collected by flushing with PBS. Peripheral blood leukocytes (PBL) were collected by heart puncture and FicollHypaque centrifugation.

### Sphere and holoclone selection

Capan1 cells (10^3^/ml) were seeded in serum-free RPMI1640 on 0.5% agar precoated 6-well plates. After 1wk half of the medium was exchanged every third day. Spheres were counted after 3wk. Single spheres were picked, dispersed and further passaged. A818.4 and SW480 cells do not or very poorly grow as spheres. Therefore CIC were enriched by holoclone formation. For holoclone selection, 50 cells / cm^2^ were seeded in 3ml RPMI1640/5% FCS in 6 well plates. After 2wk, holoclones were picked and recultured or were counted after crystal violet staining. After 3 rounds of spheroid growth and holoclone formation, spheres / holoclones were harvested and were used for 2 rounds of TEX collection (2×48h with a recovery period of 24h between). Thereafter spheroid-derived cells and holoclones were discarded.

### TEX preparation

Cells were cultured (48h) in serum-free medium. Cleared supernatants (2×10min, 500g, 1×20min, 2000g, 1×30min, 10000g) were centrifuged (90min, 100000g), washed (PBS, 90min, 100000g), resuspended and purified by sucrose gradient centrifugation. Where indicated, TEX were labeled with SP-Dio_18_(3) or DHPE [102].

### Immunoprecipitation (IP) and western blot (WB)

Lysates (IP: cell lysate 500μg, TEX lysate: 100μg; WB: cell lysate 30μg, TEX lysate: 10μg) (30min, 4°C, HEPES buffer, 1% Lubrol or 1% TritonX-100, 1mM PMSF, 1mM NaVO_4_, 10mM NaF, protease inhibitor mix) were centrifuged (13000g, 10min, 4°C), mixed with antibody (1h, 4°C) and incubated with ProteinG-Sepharose (1h). Washed complexes/lysates, dissolved in Laemmli buffer, were resolved on 10%-12% SDS-PAGE. After protein transfer, blocking, blotting with antibodies, blots were developed with ECL.

### Zymography

TEX (20μg) were incubated with Laemmli buffer (15min, 37°C) and separated in a 10% acrylamide gel containing 1mg/ml gelatin. After washing (2.5% Triton), gels were incubated in developing buffer (37°C, 48h) and stained with Coomassie-blue.

### Flow-cytometry

Flow-cytometry followed routine procedures. TEX (10μg) were bound to 1μl latex beads [102]. For intracellular staining, cells/TEX were fixed and permeabilized. Samples were processed in a FACS-Calibur.

### Confocal microscopy

Cells on glass-slides were fixed, permeabilized, blocked, incubated with primary antibody, fluorochrome-conjugated secondary antibody, blocked, incubated with second, dye-labeled primary antibody and washed. Slides were mounted in Elvanol. Digitized images were generated using a Leica DMRBE microscope.

### Quantitative RT-PCR

RNA was isolated from A818.4 wt and CD44v6 kd exosomes using the Quiazol™ reagent (Qiagen, Hilden, Germany) and quantified using the NANODROP. A total of 1 μg of RNA from each sample was used to generate single-strand cDNA using SuperScript III Reverse Transcriptase (Invitrogen™). Quantitative RT-PCR was done using SYBR Green mastermix: 95°C 10 min followed by 40 cycles of 95°C 15 sec, 60°C 1 min, 95°C 15 sec. GAPDH was used as internal control. Relative Gene expression (R_Q_) was calculated using the ΔC_t_ method.

### Adhesion

F-bottom 96-well plates were coated overnight with HA (100μg/ml in PBS) or BSA (10μg/ml), coll I (10μg/ml), coll IV (10μg/ml), FN (2μg/ml), LN111 (1μg/ml) or vesicle-depleted 804G culture supernatants (no FCS), which are highly enriched in LN332 [103] (10μg/ml) in bicarbonate buffer, pH9.6. After washing and blocking for 1h with BSA (10μg/ml), cells were seeded on the matrix protein-coated plates. After incubation for 2h at 37°C, 5% CO_2_, plates were vigorously washed and adherent cells were stained with crystal-violet and lysed, evaluating OD595 photometrically. Adhesion is presented as the percentage of input cells.

### Migration

Cells, in the upper part of a Boyden chamber (RPMI/0.1%BSA), were separated from the lower part (RPMI/20%FCS) by 8μm pore size polycarbonate-membranes. After 24h the lower membrane side was stained (crystal-violet), measuring OD595 after lysis. Migration is presented as % input cells. In an *in vitro* wound healing assay, a subconfluent monolayer was scratched with a pipette tip. Wound closure was controlled by light microscopy.

### Apoptosis

Cells (1×10^5^) were grown for 48h in RPMI/10%FCS containing cisplatin. Survival was monitored by AnnexinV-APC/PI staining, MTT assay and ^3^H-thymidine uptake.

### Soft agar assay

Tumor cells in 0.3% agar were seeded on a preformed 1% agar layer counting colonies after 3wk.

### Protein identification

After SDS-PAGE, gels were stained with Coomassie. Protein digestion, sample preparation, mass spectrometry (nanoLC-ESI-MS/MS on an LTQ orbitrap) and database searches were performed as described [104].

### *In vivo* assays

SCID mice received 1×10^6^ tumor cells subcutaneously (s.c.) or matrigel embedded intraperitoneally (i.p.). Where indicated, mice received every 3^rd^ day 100μg TEX, i.v. or i.p. Mice were controlled weekly for local tumor growth, short breathing or weight loss. Animals were sacrificed, when the local tumor reached 1.5cm diameter, mice lost >10% weight or latest after 210d. Animal experiments were Government-approved (Baden-Wuerttemberg, Germany).

### Statistics

Experiments were repeated 3-times. *In vitro* studies were performed in triplicates. P values <0.05 (two-tailed Student's t-test, Kruskal-Wallis test) were considered significant.

## SUPPLEMENTARY DATA, FIGURES AND TABLES





## Abbreviations

BMC: bone marrow cells
CD44v6: CD44 variant isoform v6
CIC: cancer initiating cells
cld: claudin
CoCa: colon carcinoma
coll: collagen
ECM: extracellular matrix
ERM: ezrin-radixin-moesin
Exo: exosomes
FN: fibronectin
GEM: glycolipid-enriched membrane microdomains
HA: hyaluronic acid
HAS: HA synthase
HGF: hepatocyte growth factor
Hyal: hyaluronidase
ICD: intracellular domain
i.p.: intraperitoneally
IP: immunoprecipitation
kd: knockdown
MDR: multidrug resistance gene
MDSC: myeloid-derived suppressor cells
MMP: metalloproteinase
LN: laminin
OPN: osteopontin
PaCa: pancreatic ductal adenocarcinoma
PBL: peripheral blood leukocytes
PDGFR: platelet-derived growth factor receptor
PEC: peritoneal exudate cells
RTK: receptor tyrosine kinase
s.c.: subcutaneously
TEX: tumor exosomes
VEGF: vascular endothelial growth factor
WB: Western blot
wt: wild type.

## References

[1] Das S, Batra SK (2015). Pancreatic cancer metastasis: are we being pre-EMTed?. Curr Pharm Des.

[2] Fakih MG (2015). Metastatic colorectal cancer: current state and future directions. J Clin Oncol.

[3] Kim J, Tanner K (2015). Recapitulating the Tumor Ecosystem Along the Metastatic Cascade Using 3D Culture Models. Front Oncol.

[4] Fitzgerald TL, McCubrey JA (2014). Pancreatic cancer stem cells: association with cell surface markers prognosis resistance metastasis and treatment. Adv Biol Regul.

[5] Zeuner A, Todaro M, Stassi G, De Maria R (2014). Colorectal cancer stem cells: from the crypt to the clinic. Cell Stem Cell.

[6] Weiswald LB, Bellet D, Dangles-Marie V (2015). Spherical cancer models in tumor biology. Neoplasia.

[7] Harper LJ, Piper K, Common J, Fortune F, Mackenzie IC (2007). Stem cell patterns in cell lines derived from head and neck squamous cell carcinoma. J Oral Pathol Med.

[8] Allegra A, Alonci A, Penna G, Innao V, Gerace D, Rotondo F, Musolino C (2014). The cancer stem cell hypothesis: a guide to potential molecular targets. Cancer Invest.

[9] Wang H, Rana S, Giese N, Büchler MW, Zöller M (2013). Tspan8 CD44v6 and alpha6beta4 are biomarkers of migrating pancreatic cancer-initiating cells. Int J Cancer.

[10] Madhavan B, Yue S, Galli U, Rana S, Gross W, Müller M, Giese NA, Kalthoff H, Becker T, Büchler MW, Zöller M (2015). Combined evaluation of a panel of protein and miRNA serum-exosome biomarkers for pancreatic cancer diagnosis increases sensitivity and specificity. Int J Cancer.

[11] Li C, Wu JJ, Hynes M, Dosch J, Sarkar B, Welling TH, Pasca di Magliano M, Simeone DM (2011). c-Met is a marker of pancreatic cancer stem cells and therapeutic target. Gastroenterology.

[12] Saito S, Okabe H, Watanabe M, Ishimoto T, Iwatsuki M, Baba Y, Tanaka Y, Kurashige J, Miyamoto Y, Baba H (2013). CD44v6 expression is related to mesenchymal phenotype and poor prognosis in patients with colorectal cancer. Oncol Rep.

[13] Todaro M, Gaggianesi M, Catalano V, Benfante A, Iovino F, Biffoni M, Apuzzo T, Sperduti I, Volpe S, Cocorullo G, Gulotta G, Dieli F, De Maria R, Stassi G (2014). CD44v6 is a marker of constitutive and reprogrammed cancer stem cells driving colon cancer metastasis. Cell Stem Cell.

[14] Aruffo A, Stamenkovic I, Melnick M, Underhill CB, Seed B (1990). CD44 is the principal cell surface receptor for hyaluronate. Cell.

[15] Ishii S, Ford R, Thomas P, Nachman A, Steele G, Jessup JM (1993). CD44 participates in the adhesion of human colorectal carcinoma cells to laminin and type IV collagen. Surg Oncol.

[16] Jalkanen S, Jalkanen M (1992). Lymphocyte CD44 binds the COOH-terminal heparin-binding domain of fibronectin. J Cell Biol.

[17] Orian-Rousseau V, Sleeman J (2014). CD44 is a multidomain signaling platform that integrates extracellular matrix cues with growth factor and cytokine signals. Adv Cancer Res.

[18] Williams K, Motiani K, Giridhar PV, Kasper S (2013). CD44 integrates signaling in normal stem cell cancer stem cell and (pre)metastatic niches. Exp Biol Med.

[19] Orian-Rousseau V, Morrison H, Matzke A, Kastilan T, Pace G, Herrlich P, Ponta H (2007). Hepatocyte growth factor-induced Ras activation requires ERM proteins linked to both CD44v6 and F-actin. Mol Biol Cell.

[20] Jung T, Gross W, Zöller M (2011). CD44v6 coordinates tumor matrix-triggered motility and apoptosis resistance. J Biol Chem.

[21] Ni J, Cozzi PJ, Hao JL, Beretov J, Chang L, Duan W, Shigdar S, Delprado WJ, Graham PH, Bucci J, Kearsley JH, Li Y (2014). CD44 variant 6 is associated with prostate cancer metastasis and chemo-/radioresistance. Prostate.

[22] Hasenauer S, Malinger D, Koschut D, Pace G, Matzke A, von Au A, Orian-Rousseau V (2013). Internalization of Met requires the co-receptor CD44v6 and its link to ERM proteins. PLoS One.

[23] Porsch H, Mehić M, Olofsson B, Heldin P, Heldin CH (2014). Platelet-derived growth factor β-receptor transforming growth factor β type I receptor and CD44 protein modulate each other's signaling and stability. J Biol Chem.

[24] Weber GF, Bronson RT, Ilagan J, Cantor H, Schmits R, Mak TW (2002). Absence of the CD44 gene prevents sarcoma metastasis. Cancer Res.

[25] Mori T, Kitano K, Terawaki S, Maesaki R, Fukami Y, Hakoshima T (2008). Structural basis for CD44 recognition by ERM proteins. J Biol Chem.

[26] Oliferenko S, Paiha K, Harder T, Gerke V, Schwärzler C, Schwarz H, Beug H, Günthert U, Huber LA (1999). Analysis of CD44-containing lipid rafts: Recruitment of annexin II and stabilization by the actin cytoskeleton. J Cell Biol.

[27] Zöller M (2011). CD44: can a cancer-initiating cell profit from an abundantly expressed molecule?. Nat Rev Cancer.

[28] Róg T, Vattulainen I (2014). Cholesterol sphingolipids and glycolipids: What do we know about their role in raft-like membranes?. Chem Phys Lipids.

[29] Kuhn NZ, Tuan RS (2010). Regulation of stemness and stem cell niche of mesenchymal stem cells: implications in tumorigenesis and metastasis. J Cell Physiol.

[30] Klingbeil P, Marhaba R, Jung T, Kirmse R, Ludwig T, Zöller M (2009). CD44 variant isoforms promote metastasis formation by a tumor cell-matrix cross-talk that supports adhesion and apoptosis resistance. Mol Cancer Res.

[31] Jung T, Castellana D, Klingbeil P, Cuesta Hernández I, Vitacolonna M, Orlicky DJ, Roffler SR, Brodt P, Zöller M (2009). CD44v6 dependence of premetastatic niche preparation by exosomes. Neoplasia.

[32] Adamia S, Maxwell CA, Pilarski LM (2005). Hyaluronan and hyaluronan synthases: potential therapeutic targets in cancer. Curr Drug Targets Cardiovasc Haematol Disord.

[33] Miletti-González KE, Murphy K, Kumaran MN, Ravindranath AK, Wernyj RP, Kaur S, Young LJ, Muller WJ, Miles GD, Lim E, Chan R, Chekmareva M, Heller DS, Foran D, Chen W (2012). Identification of function for CD44 intracytoplasmic domain (CD44-ICD): modulation of matrix metalloproteinase 9 (MMP-9) transcription via novel promoter response element. J Biol Chem.

[34] Desai B, Ma T, Zhu J, Chellaiah MA (2009). Characterization of the expression of variant and standard CD44 in prostate cancer cells: identification of the possible molecular mechanism of CD44/MMP9 complex formation on the cell surface. J Cell Biochem.

[35] Bourguignon LY, Gunja-Smith Z, Iida N, Zhu HB, Young LJ, Muller WJ, Cardiff RD (1998). CD44v(3,8-10) is involved in cytoskeleton-mediated tumor cell migration and matrix metalloproteinase (MMP-9) association in metastatic breast cancer cells. J Cell Physiol.

[36] Zarzynska JM (2014). Two FACes of TGF-beta1 in breast cancer. Mediators Inflamm.

[37] Lässer C (2015). Exosomes in diagnostic and therapeutic applications: biomarker vaccine and RNA interference delivery vehicle. Expert Opin Biol Ther.

[38] Kreimer S, Belov AM, Ghiran I, Murthy SK, Frank DA, Ivanov AR (2015). Mass-spectrometry-based molecular characterization of extracellular vesicles: lipidomics and proteomics. J Proteome Res.

[39] Lo Cicero A, Stahl PD, Raposo G (2015). Extracellular vesicles shuffling intercellular messages: for good or for bad. Curr Opin Cell Biol.

[40] Kim DK, Lee J, Simpson RJ, Lötvall J, Gho YS (2015). EVpedia: A community web resource for prokaryotic and eukaryotic extracellular vesicles research. Semin Cell Dev Biol.

[41] Zhang J, Li S, Li L, Li M, Guo C, Yao J, Mi S (2015). Exosome and exosomal microRNA: trafficking sorting and function. Genomics Proteomics Bioinformatics.

[42] Quesenberry PJ, Aliotta J, Deregibus MC, Camussi G (2015). Role of extracellular RNA-carrying vesicles in cell differentiation and reprogramming. Stem Cell Res Ther.

[43] Thuma F, Zöller M (2014). Outsmart tumor exosomes to steal the cancer initiating cell its niche. Semin Cancer Biol.

[44] Costa-Silva B, Aiello NM, Ocean AJ, Singh S, Zhang H, Thakur BK, Becker A, Hoshino A, Mark MT, Molina H, Xiang J, Zhang T, Theilen TM (2015). Pancreatic cancer exosomes initiate pre-metastatic niche formation in the liver. Nat Cell Biol.

[45] Vella LJ (2014). The emerging role of exosomes in epithelial-mesenchymal-transition in cancer. Front Oncol.

[46] Nazarenko I, Rana S, Baumann A, McAlear J, Hellwig A, Trendelenburg M, Lochnit G, Preissner KT, Zöller M (2010). Molecular pathways of exosome-induced angiogenesis and the contribution of the tetraspanin Tspan8. Cancer Res.

[47] Liu Y, Gu Y, Cao X (2015). The exosomes in tumor immunity. Oncoimmunology.

[48] Kuhn S, Koch M, Nübel T, Ladwein M, Antolovic D, Klingbeil P, Hildebrand D, Moldenhauer G, Langbein L, Franke WW, Weitz J, Zöller M (2007). A complex of EpCAM claudin-7 CD44 variant isoforms and tetraspanins promotes colorectal cancer progression. Mol Cancer Res.

[49] Zöller M (2009). Tetraspanins: push and pull in suppressing and promoting metastasis. Nat Rev Cancer.

[50] Rana S, Zöller M (2011). Exosome target cell selection and the importance of exosomal tetraspanins: a hypothesis. Biochem Soc Trans.

[51] Ginsberg MH (2014). Integrin activation. BMB Rep.

[52] Mitra SK, Schlaepfer DD (2006). Integrin-regulated FAK-Src signaling in normal and cancer cells. Curr Opin Cell Biol.

[53] Liu Y, Gu Y, Cao X (2015). The exosomes in tumor immunity. Oncoimmunology.

[54] Zech D, Rana S, Büchler MW, Zöller M (2012). Tumor-exosomes and leukocyte activation: an ambivalent crosstalk. Cell Commun Signal.

[55] Bourguignon LY, Shiina M, Li JJ (2014). Hyaluronan-CD44 interaction promotes oncogenic signaling microRNA functions chemoresistance and radiation resistance in cancer stem cells leading to tumor progression. Adv Cancer Res.

[56] Chanmee T, Ontong P, Kimata K, Itano N (2015). Key Roles of Hyaluronan and Its CD44 Receptor in the Stemness and Survival of Cancer Stem Cells. Front Oncol.

[57] van Hinsbergh VW, Engelse MA, Quax PH (2006). Pericellular proteases in angiogenesis and vasculogenesis. Arterioscler Thromb Vasc Biol.

[58] Yan Y, Zuo X, Wei D (2015). Concise Review: Emerging Role of CD44 in Cancer Stem Cells: A Promising Biomarker and Therapeutic Target. Stem Cells Transl Med.

[59] Karim BO, Rhee KJ, Liu G, Yun K, Brant SR (2014). Prom1 function in development intestinal inflammation and intestinal tumorigenesis. Front Oncol.

[60] Thuma F, Zöller M (2013). EpCAM-associated claudin-7 supports lymphatic spread and drug resistance in rat pancreatic cancer. Int J Cancer.

[61] Horta S, Agostinho AL, Mateus R, Pereira L, Pereira C, Capinha L, Doktorovova S, Brito A, Videira M (2015). Looking out for cancer stem cells' properties: the value-driving role of CD44 for personalized medicines. Curr Cancer Drug Targets.

[62] Masui T, Ota I, Yook JI, Mikami S, Yane K, Yamanaka T, Hosoi H (2014). Snail-induced epithelial-mesenchymal transition promotes cancer stem cell-like phenotype in head and neck cancer cells. Int J Oncol.

[63] Su J, Wu S, Wu H, Li L, Guo T (2016). CD44 is functionally crucial for driving lung cancer stem cells metastasis through Wnt/β-catenin-FoxM1-Twist signaling. Mol Carcinog.

[64] Shigeishi H, Biddle A, Gammon L, Emich H, Rodini CO, Gemenetzidis E, Fazil B, Sugiyama M, Kamata N, Mackenzie IC (2013). Maintenance of stem cell self-renewal in head and neck cancers requires actions of GSK3β influenced by CD44 and RHAMM. Stem Cells.

[65] Hemler ME (2005). Tetraspanin functions and associated microdomains. Nat Rev Mol Cell Biol.

[66] Stipp CS (2010). Laminin-binding integrins and their tetraspanin partners as potential antimetastatic targets. Expert Rev Mol Med.

[67] Su YJ, Lin WH, Chang YW, Wei KC, Liang CL, Chen SC, Lee JL (2015). Polarized cell migration induces cancer type-specific CD133/integrin/Src/Akt/GSK3β/β-catenin signaling required for maintenance of cancer stem cell properties. Oncotarget.

[68] Heiler S, Mu W, Zöller M, Thuma F (2015). The importance of claudin-7 palmitoylation on membrane subdomain localization and metastasis-promoting activities. Cell Commun Signal.

[69] Andreu Z, Yáñez-Mó M (2014). Tetraspanins in extracellular vesicle formation and function. Front Immunol.

[70] Negi LM, Talegaonkar S, Jaggi M, Ahmad FJ, Iqbal Z, Khar RK (2012). Role of CD44 in tumour progression and strategies for targeting. J Drug Target.

[71] McFarlane S, McFarlane C, Montgomery N, Hill A, Waugh DJ (2015). CD44-mediated activation of α5β1-integrin cortactin and paxillin signaling underpins adhesion of basal-like breast cancer cells to endothelium and fibronectin-enriched matrices. Oncotarget.

[72] Shen B, Delaney MK, Du X (2012). Inside-out outside-in and inside-outside-in: G protein signaling in integrin-mediated cell adhesion spreading and retraction. Curr Opin Cell Biol.

[73] Zhao X, Guan JL (2011). Focal adhesion kinase and its signaling pathways in cell migration and angiogenesis. Adv Drug Deliv Rev.

[74] Takahashi E, Nagano O, Ishimoto T, Yae T, Suzuki Y, Shinoda T, Nakamura S, Niwa S, Ikeda S, Koga H, Tanihara H, Saya H (2010). Tumor necrosis factor-alpha regulates transforming growth factor-beta-dependent epithelial-mesenchymal transition by promoting hyaluronan-CD44-moesin interaction. J Biol Chem.

[75] Jiang W, Zhang Y, Kane KT, Collins MA, Simeone DM, di Magliano MP, Nguyen KT (2015). CD44 regulates pancreatic cancer invasion through MT1-MMP. Mol Cancer Res.

[76] Kung CI, Chen CY, Yang CC, Lin CY, Chen TH, Wang HS (2012). Enhanced membrane-type 1 matrix metalloproteinase expression by hyaluronan oligosaccharides in breast cancer cells facilitates CD44 cleavage and tumor cell migration. Oncol Rep.

[77] Kamarajan P, Shin JM, Qian X, Matte B, Zhu JY, Kapila YL (2013). ADAM17-mediated CD44 cleavage promotes orasphere formation or stemness and tumorigenesis in HNSCC. Cancer Med.

[78] Yáñez-Mó M, Gutiérrez-López MD, Cabañas C (2011). Functional interplay between tetraspanins and proteases. Cell Mol Life Sci.

[79] Mu W, Rana S, Zöller M (2013). Host matrix modulation by tumor exosomes promotes motility and invasiveness. Neoplasia.

[80] Yue S, Mu W, Erb U, Zöller M (2015). The tetraspanins CD151 and Tspan8 are essential exosome components for the crosstalk between cancer initiating cells and their surrounding. Oncotarget.

[81] Małgorzewicz S, Skrzypczak-Jankun E, Jankun J (2013). Plasminogen activator inhibitor-1 in kidney pathology. Int J Mol Med.

[82] Weidle UH, Birzele F, Krüger A (2015). Molecular targets and pathways involved in liver metastasis of colorectal cancer. Clin Exp Metastasis.

[83] Cui H, Seubert B, Stahl E, Dietz H, Reuning U, Moreno-Leon L, Ilie M, Hofman P, Nagase H, Mari B, Krüger A (2015). Tissue inhibitor of metalloproteinases-1 induces a pro-tumourigenic increase of miR-210 in lung adenocarcinoma cells and their exosomes. Oncogene.

[84] Tania M, Khan MA, Fu J (2014). Epithelial to mesenchymal transition inducing transcription factors and metastatic cancer. Tumour Biol.

[85] Stamenkovic I, Yu Q (2010). Merlin a “magic” linker between extracellular cues and intracellular signaling pathways that regulate cell motility proliferation and survival. Curr Protein Pept Sci.

[86] Gucciardo E, Sugiyama N, Lehti K (2014). Eph- and ephrin-dependent mechanisms in tumor and stem cell dynamics. Cell Mol Life Sci.

[87] Heldin CH, Lennartsson J (2013). Structural and functional properties of platelet-derived growth factor and stem cell factor receptors. Cold Spring Harb Perspect Biol.

[88] Choi S, Kang DH, Oh ES (2013). Targeting syndecans: a promising strategy for the treatment of cancer. Expert Opin Ther Targets.

[89] Secker GA, Harvey NL (2015). VEGFR signaling during lymphatic vascular development: From progenitor cells to functional vessels. Dev Dyn.

[90] Jackson DG (2004). Biology of the lymphatic marker LYVE-1 and applications in research into lymphatic trafficking and lymphangiogenesis. APMIS.

[91] Riabov V, Gudima A, Wang N, Mickley A, Orekhov A, Kzhyshkowska J (2014). Role of tumor associated macrophages in tumor angiogenesis and lymphangiogenesis. Front Physiol.

[92] Bhatia A, Kumar Y (2014). Cellular and molecular mechanisms in cancer immune escape: a comprehensive review. Expert Rev Clin Immunol.

[93] Penfornis P, Vallabhaneni KC, Whitt J, Pochampally R (2016). Extracellular vesicles as carriers of microRNA proteins and lipids in tumor microenvironment. Int J Cancer.

[94] Hannafon BN, Ding WQ (2013). Intercellular communication by exosome-derived microRNAs in cancer. Int J Mol Sci.

[95] Kharaziha P, Ceder S, Li Q, Panaretakis T (2012). Tumor cell-derived exosomes: a message in a bottle. Biochim Biophys Acta.

[96] Rana S, Malinowska K, Zöller M (2013). Exosomal tumor microRNA modulates premetastatic organ cells. Neoplasia.

[97] Mollinedo F, Gajate C (2015). Lipid rafts as major platforms for signaling regulation in cancer. Adv Biol Regul.

[98] Zöller M (2016). Exosomes in Cancer Disease. Methods Mol Biol.

[99] Lehnert L, Lerch MM, Hirai Y, Kruse ML, Schmiegel W, Kalthoff H (2001). Autocrine stimulation of human pancreatic duct-like development by soluble isoforms of epimorphin in vitro. J Cell Biol.

[100] Fogh J, Wright WC, Loveless JD (1977). Absence of HeLa cell contamination in 169 cell lines derived from human tumors. J Natl Cancer Inst.

[101] Trainer DL, Kline T, McCabe FL, Faucette LF, Feild J, Chaikin M, Anzano M, Rieman D, Hoffstein S, Li DJ (1988). Biological characterization and oncogene expression in human colorectal carcinoma cell lines. Int J Cancer.

[102] Rana S, Yue S, Stadel D, Zöller M (2012). Toward tailored exosomes: the exosomal tetraspanin web contributes to target cell selection. Int J Biochem Cell Biol.

[103] Homma Y, Ozono S, Numata I, Seidenfeld J, Oyasu R (1985). α-Difluoromethylornithine inhibits cell growth stimulated by a tumor-promoting rat urinary fraction. Carcinogenesis.

[104] Junwei L, Bonifati S, Hristov G, Martilla T, Valmary-Degano S, Stanzel S, Schnölzer M, Mougin C, Aprahamian M, Grekova S, Raykov Z, Rommelaere J, Marchini A (2013). Synergistic combination of valproic acid and oncolytic parvovirus H-1PV as a potential therapy against cervical and pancreatic carcinomas. EMBO Mol Med.

