# Supplementary material for: CD44v6-competent tumor exosomes promote motility, invasion and cancer-initiating cell marker expression in pancreatic and colorectal cancer cells
Source: Oncotarget. 2016 Jul 13;7(34):55409–36. doi: 10.18632/oncotarget.10580 (PMC5342426; doi:10.18632/oncotarget.10580)
Supplement: Supplementary file 1 [file oncotarget-07-55409-s001.pdf]

CD44v6-competent tumor exosomes promote motility, invasion and cancer-initiating cell marker expression in pancreatic and colorectal cancer cells

SUPPLEMENTARY DATA

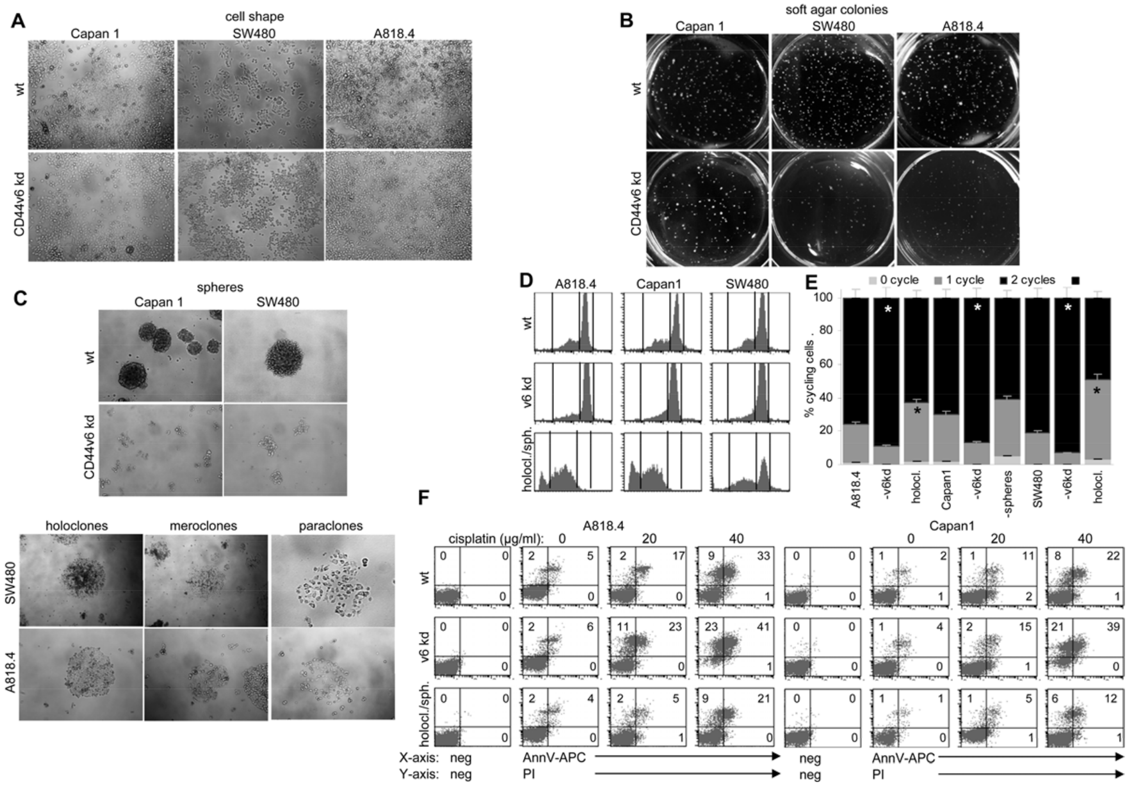

Supplementary Figure S1: Impact of CD44v6 expression on growth features of cancer cell lines. Capan1, SW480 and A818.4 wt and CD44v6^{kd} cells were characterized for **A.** morphological appearance during growth on plastic, **B.** the capacity for anchorage independent growth, **C.** sphere or holoclone formation, **D.** cell cycle progression, **E.** cell division as judged by CFSE dilution and **F.** AnnV/PI staining after 48h culture in the absence or presence of cisplatin. (A, B, C, D, F) representative examples; (E) mean±SD of triplicates, significant differences to wt cells: *.

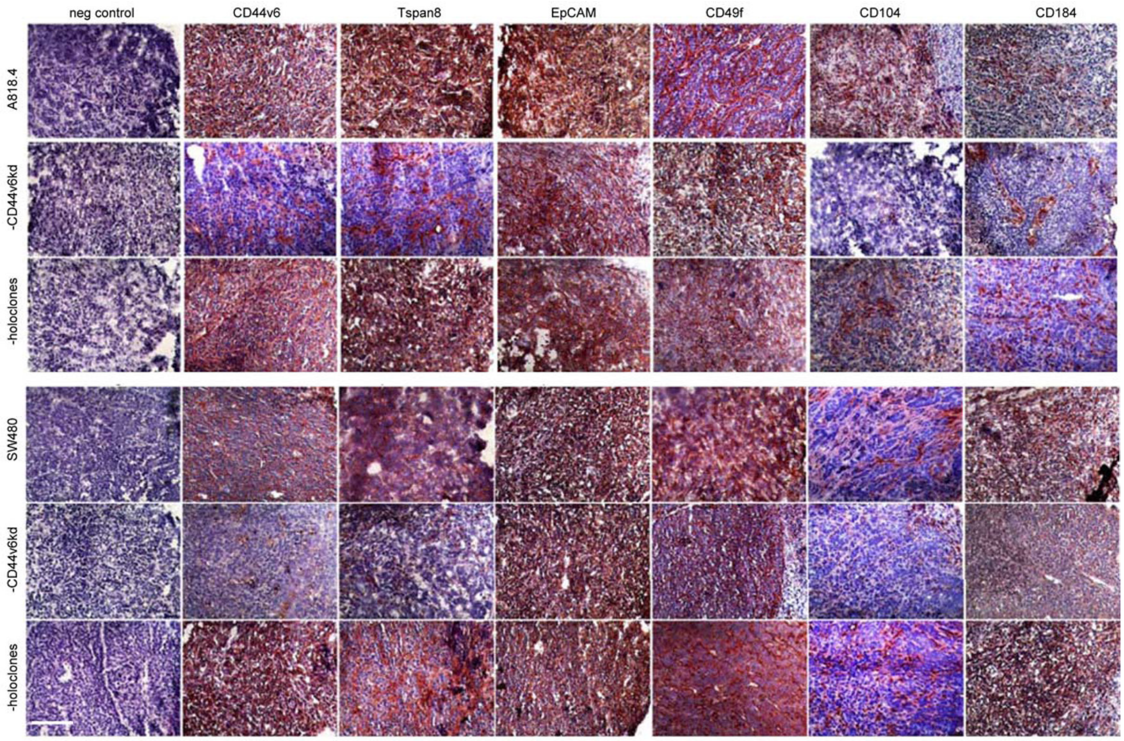

Supplementary Figure S2: The impact of CD44v6 on CIC marker expression in vivo. Wt, CD44v6^{kd} and holoclone A818.4 and SW480 cells were s.c. injected. Sections of shock frozen tumors were stained with CD44v6, Tspan8, EpCAM, CD49f, CD104 and CD184 (scale bar: 100µm). With the exception of EpCAM, expression of CIC markers is higher in holoclone than wt tumors, expression in CD44v6^{kd} tumors is reduced.

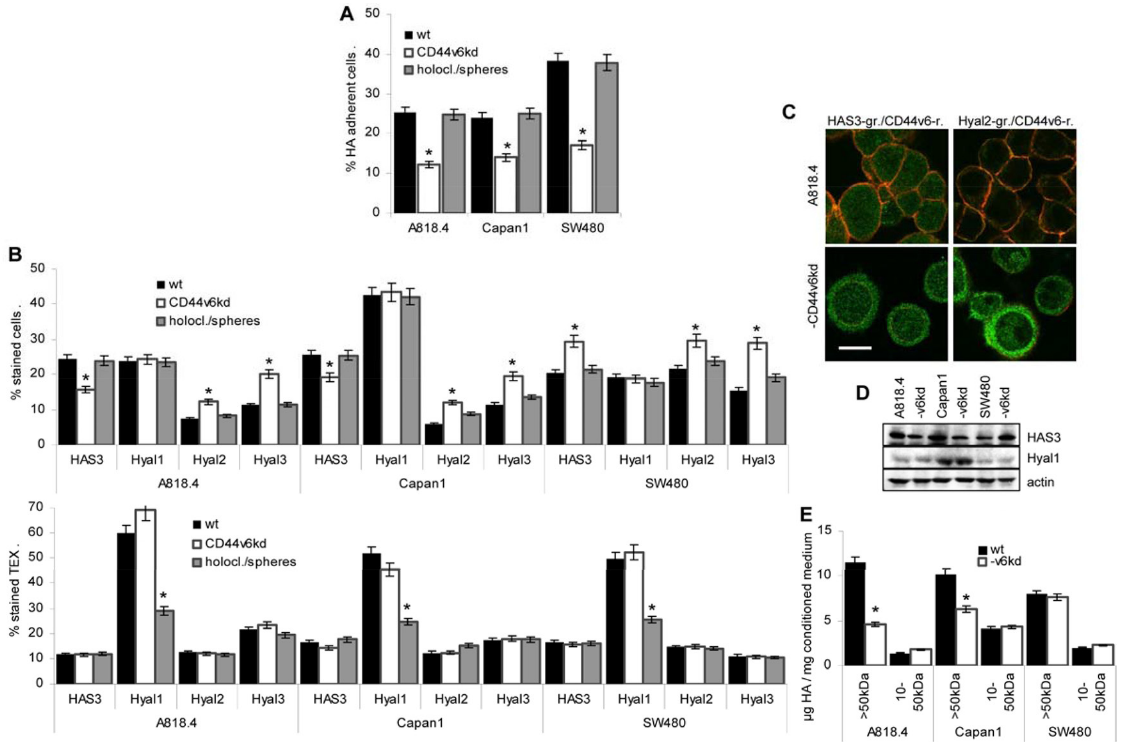

Supplementary Figure S3: CD44v6 and HA adhesion. A. Adhesion of A818.4, Capan1, SW480 wt, CD44v6^{kd} and holoclone/sphere cells to HA-coated plates. **B.** Flow-cytometry of HAS3, Hyal1, Hyal2 and Hyal3 expression in wt, CD44v6^{kd} and sphere/holoclone cells and TEX. **C.** Confocal microscopy of CD44v6 and HAS3 and Hyal2 staining in A818.4 wt and -CD44v6^{kd} cells. Overlays of CD44v6 (red) and HAS3 / Hyal2 (green) staining are shown (scale bar: 10µm). **D.** WB of HAS3 and Hyal1 in wt and CD44v6^{kd} lysates. **E.** HA in culture supernatant determined by ELISA after size fractionation by filtration. (A, B, E) Mean±SD of triplicates; significant differences between wt, CD44v6^{kd}, sphere/holoclone cells, TEX and culture supernatant: *. HA binding of CD44v6^{kd} cells is significantly reduced. Reduced binding might be linked to upregulated Hyal2 and Hyal3 expression, which, however was not transferred to TEX. Also, HMW HA was only decreased in A818.4- and Capan1-CD44v6^{kd} supernatant. The findings argue against CD44v6 - HA binding playing a central role in PaCa / CoCa adhesion.

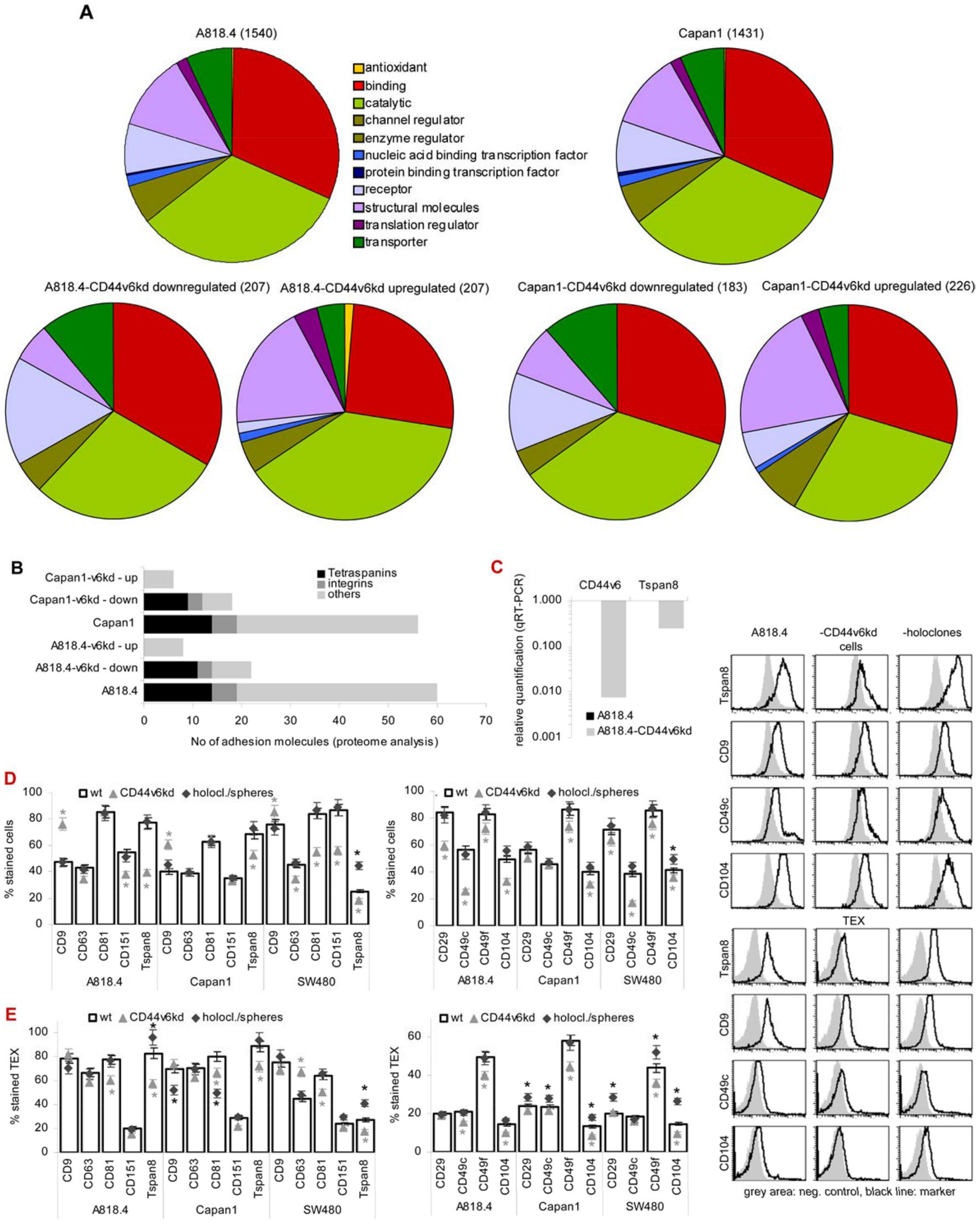

Supplementary Figure S4: Recovery of adhesion molecules in CD44v6^{kd} cells by mass-spectrometry. Lysates of A818.4 and Capan1 wt and CD44v6^{kd} cells were separated by SDS PAGE, digested and analyzed by nanoLC-ESI-MS/MS mass-spectrometry. **A.** Number of proteins grouped according to molecular functions in A818.4 and Capan1 wt cells and indication of down- or upregulated proteins in CD44v6^{kd} cells (Panther Pathway analysis). **B.** number of adhesion molecules in A818.4 and Capan1 cells and number of adhesion molecules strongly reduced or upregulated in CD44v6^{kd} lysates with emphasis on tetraspanins and integrins. **C.** Relative quantification (qRT-PCR) of CD44v6 and Tspan8 in A818.4 wt and -CD44v6^{kd} cells. **D, E.** Tetraspanin and integrin recovery in cells and TEX (flow-cytometry); mean values of triplicates±SD and representative examples are shown; significant differences between wt cells / TEX and CD44v6^{kd} and holoclone/sphere cells and TEX; *. There is a striking overpresentation of tetraspanin and integrin downregulation in CD44v6^{kd} cells and TEX, where distinct to other tetraspanins, CD9 is upregulated in CD44v6^{kd} cells. As explored for Tspan8, regulation of tetraspanin expression by CD44v6 may proceed at the transcriptional level.

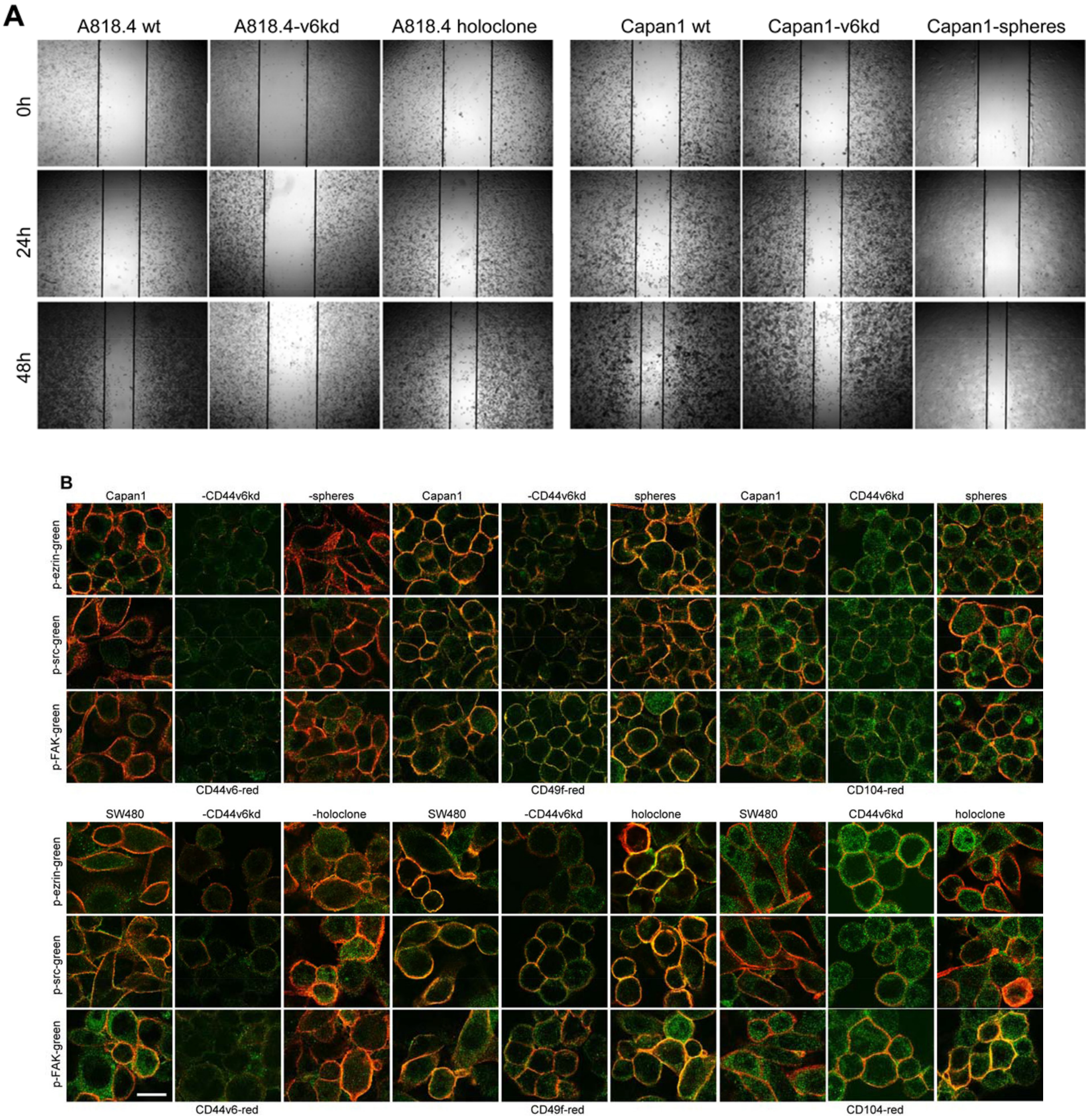

Supplementary Figure S5: Migration of wt, CD44v6^{kd} and sphere/holoclone cells. A. Representative wound healing examples of A818.4 and Capan1 wt, CD44v6^{kd}, sphere/holoclone cells. **B.** Confocal microscopy of Capan1 and SW480 wt, CD44v6^{kd}, sphere/holoclone cells stained with anti-CD44v6, -CD49f and -CD104 (red) and counterstained with anti-p-ezrin, -p-src and -p-FAK (green); overlays of green and red staining are shown (scale bar: 100µm).

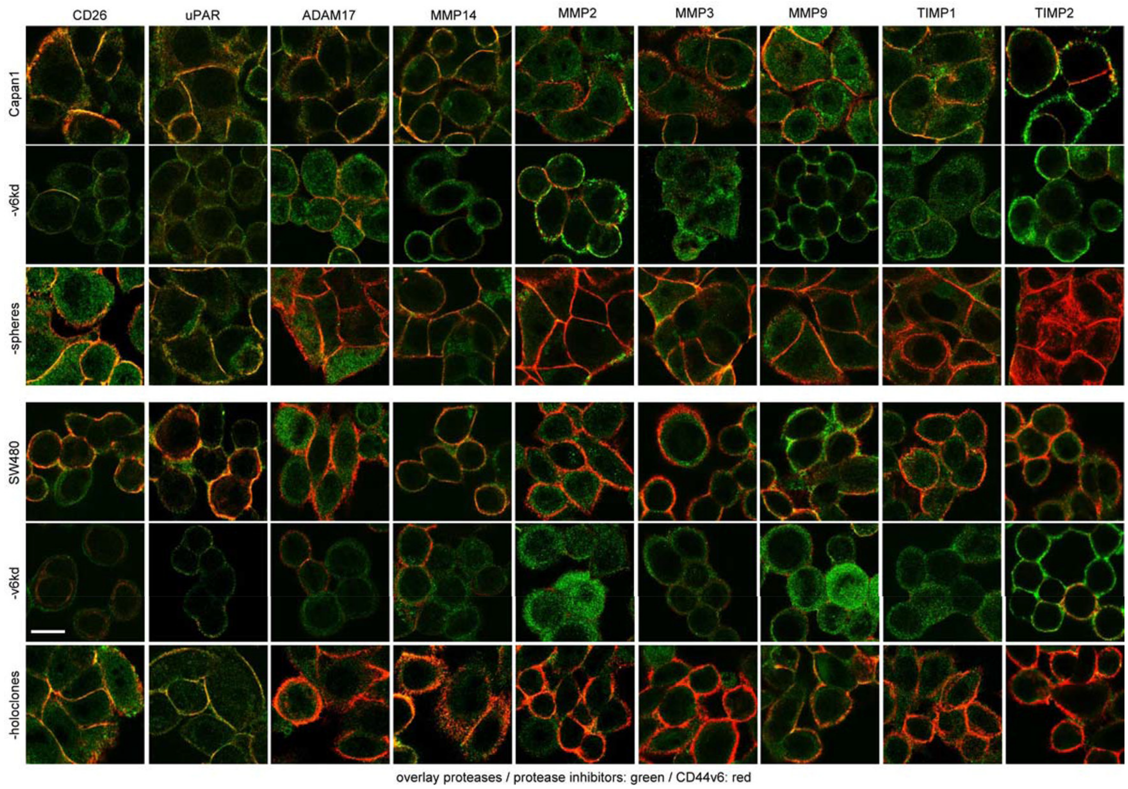

Supplementary Figure S6: Colocalization of CD44v6 and proteases: Capan1 and SW480 wt, -CD44v6^{kd} and holoclone/sphere cells were stained with anti-proteases (green) and anti-CD44v6 (red). Digital overlays are presented (scale bar: 10µm). Distinct colocalization with uPAR, ADAM17, MMP14 and MMP9 was maintained and colocalization with CD26 was increased in spheres/holoclones.

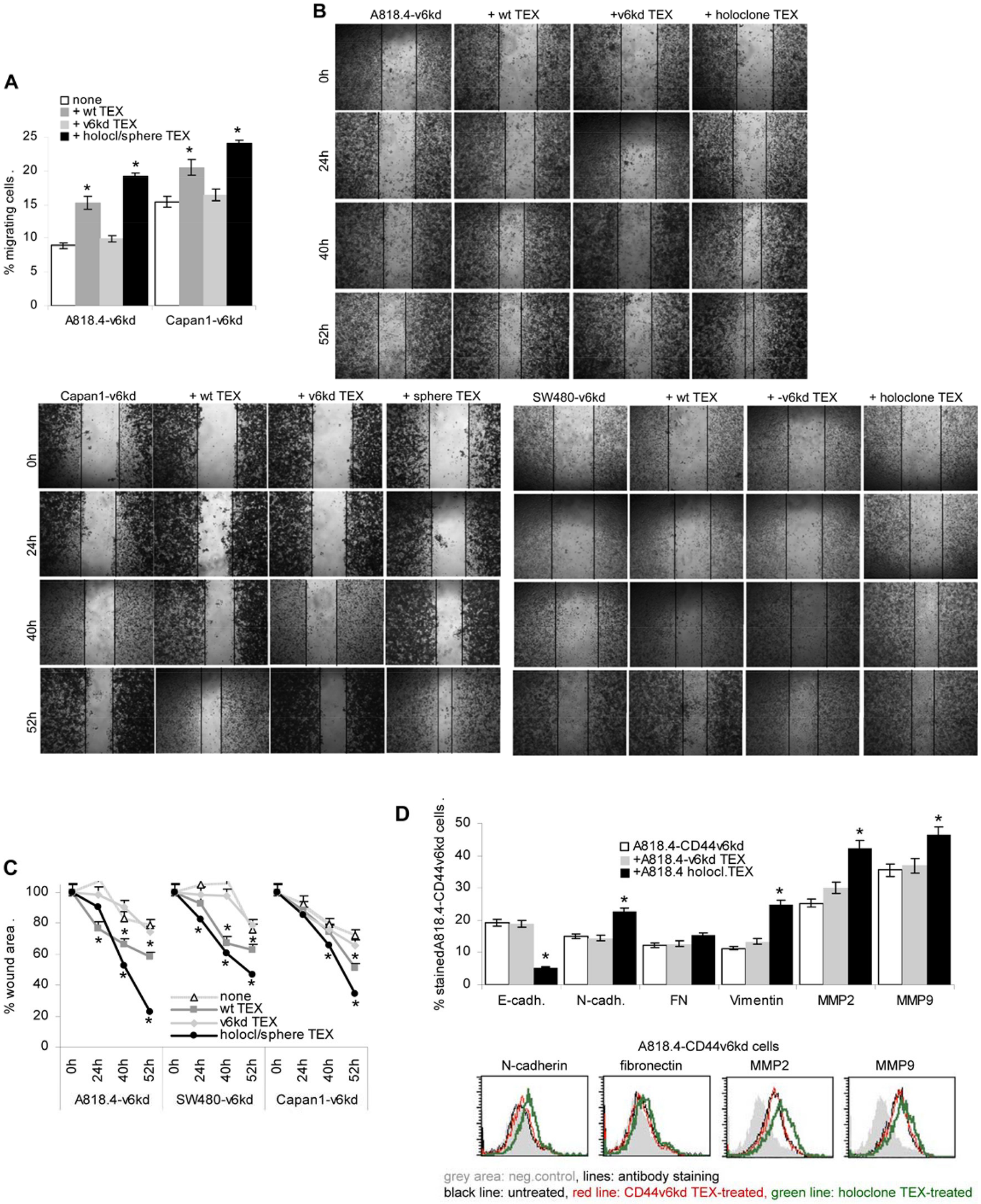

Supplementary Figure S7: TEX uptake promotes tumor cell migration. A. A818.4- and Capan1-CD44v6^{kd} cells were seeded in the upper part of a Boyden chamber, the lower part contained no TEX or TEX as indicated. Transwell migration was determined as in Figure 4A. **B, C.** Subconfluent monolayers of A818.4-, Capan1- and SW480-CD44v6^{kd} cells were scratched with a pipette tip. After wounding, medium with 10% FCS, wt, CD44v6^{kd} or sphere/holoclone TEX (20µg/ml) was added. Wound healing was recorded for 52h. (B) Representative example for A818.4-, Capan1- and SW480-CD44v6^{kd} cells and (C) mean values (triplicates)±SD of the three lines are shown. **D.** Flow cytometry analysis of EMT marker and protease expression in A818.4-CD44v6^{kd} cells cultured in the presence of absence of holoclone TEX. Mean±SD values of 3 assays are shown. (A, C, D) Significant differences to medium without TEX: *. Holoclone/sphere TEX facilitate migration of poorly migrating CD44v6^{kd} cells, which is accompanied by upregulation of several EMT markers and proteases.

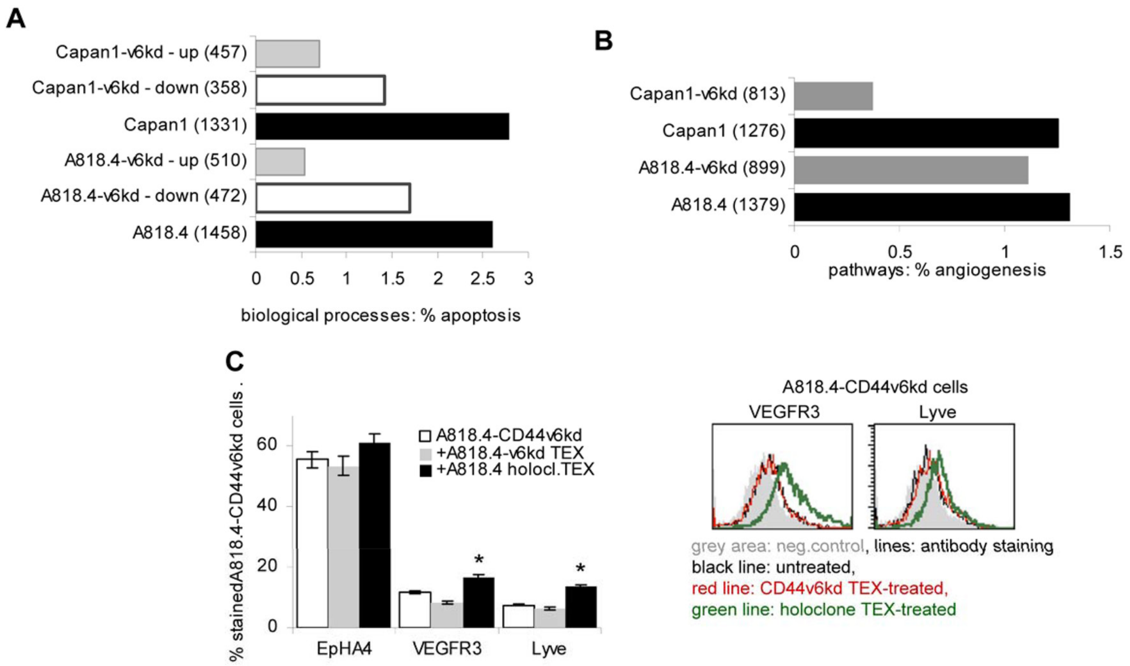

Supplementary Figure S8: Proteome analysis of CD44v6-dependent changes molecules engaged in apoptosis and angiogenesis. A. The percent of apoptosis-related molecules in A818.4, Capan1 and -CD44v6^{kd} cells (proteome analysis) and **B.** the percent of angiogenesis-related molecules in A818.4, Capan1 and -CD44v6^{kd} cells (proteome analysis) are shown. **C.** Flow-cytometry of EphA4, VEGFR3 and Lyve expression in A818.4-CD44v6^{kd} cells after 48h coculture with TEX. The mean±SD of 3 assays and representative examples are shown. Significant differences by coculture with holoclone TEX: *. There is evidence for subtle differences between wt and CD44v6^{kd} cells being "corrected" by holoclone TEX.

Supplementary Table S1: Proteome analysis of wt and CD44v6kd pancreatic cancer cell lines

Supplementary Table S1A: Proteins downregulated in CD44v6^{kd} cells

Supplementary Table S1B: Proteins upregulated in CD44v6^{kd} cells

Supplementary Table S1C: Proteins opposingly regulated in CD44v6^{kd} cells

See Supplementary File 1

Supplementary Table S2: Primers

CD44v6^{kd} primers
CD44v6 fw: ACCGGTGTAGTACAACCTGCGGAAGAACGAATTCTTCCGCAGGTTGTACTAC
CD44v6 rev: GAATTCGTAGTACAACCTGCGGAAGAATTCGTTCTTCCGCAGGTTGTACTAC

qRT-PCR primers
Tspan8 fw: gcttgcttctgatcctgctc
Tspan8 rev: attgaccaaaccgcagcatt

CD44v6 fw: gtttctagaatgagtcgaagaaggtgtggg
CD44v6 rev: gttggatccttacaccccaatcttcatgtc

GAPDH fw: aggtcggagtcaacggattt
GAPDH rev: atctcgctcctggaagatgg

Supplementary Table S3: Antibodies and Reagents

See Supplementary File 1
