# Supplementary material for: CD44v6-competent tumor exosomes promote motility, invasion and cancer-initiating cell marker expression in pancreatic and colorectal cancer cells
Source: Oncotarget. 2016 Jul 13;7(34):55409–36. doi: 10.18632/oncotarget.10580 (PMC5342426; doi:10.18632/oncotarget.10580)
Supplement: Supplementary file 2 [file oncotarget-07-55409-s002.doc]

Supplementary Table 1

**Proteome analysis of wt and CD44v6kd pancreatic cancer cell lines**

**Suppl. Table 1A. Proteins downregulated in CD44v6^kd^ cells**

| **name** | **protein hits** | | | | **full name** |
| --- | --- | --- | --- | --- | --- |
|  | **A818.4** | **A818.4-v6kd** | **Capan1** | **Capan1-v6kd** |  |
| ABCC3 | **10** | **0** | **12** | **0** | Canalicular multispecific organic anion transporter 2 |
| ABI1 | 9 | 5 | **13** | **4** | Abl interactor 1 |
| ADAM10 | **436** | **113** | **369** | **157** | ADAM metallopeptidase domain 10 |
| ANXA1 | **43** | **4** | 21 | 14 | Annexin A1 |
| ANXA5 | **127** | **53** | 97 | 55 | Annexin A5 |
| AP2A1 | **17** | **0** | **12** | **0** | AP-2 complex subunit alpha-1 |
| AP2A2 | **18** | **0** | **14** | **0** | AP-2 complex subunit alpha-2 |
| AP2B1 | **33** | **0** | **25** | **3** | AP-2 complex subunit beta |
| AP2S1 | **12** | **6** | 10 | 6 | AP-2 complex subunit sigma |
| AQP5 | **12** | **0** | 4 | 0 | Aquaporin-5 |
| AREG | **22** | **0** | **10** | **5** | Amphiregulin |
| ARF6 | **33** | **16** | **37** | **16** | ADP-ribosylation factor 6 |
| ARRDC1 | **174** | **83** | 156 | 97 | Arrestin domain-containing protein 1 |
| ATP1B1 | **60** | **24** | **53** | **26** | Sodium/potassium-transporting ATPase subunit beta-1 |
| ATP2B1 | **36** | **0** | **24** | **0** | Plasma membrane calcium-transporting ATPase 1 |
| ATP2B4 | **31** | **9** | **22** | **10** | Plasma membrane calcium-transporting ATPase 4 |
| BROX | **56** | **18** | **53** | **11** | BRO1 domain-containing protein BROX |
| CA9 | **18** | **0** | **15** | **2** | Carbonic anhydrase 9 |
| CAPN5 | **29** | **0** | **26** | **5** | Calpain-5 |
| CAPN7 | **32** | **0** | **15** | **4** | Calpain-7 |
| CASK | **15** | **0** | **16** | **0** | Peripheral plasma membrane protein CASK |
| CCRL2 | **11** | **1** | 6 | 1 | C-C chemokine receptor-like 2 |
| CD146 | 72 | 37 | **95** | **11** | Cell surface glycoprotein MUC18 |
| CD151 | **44** | **18** | 38 | 24 | CD151 antigen |
| CD276 | **37** | **4** | **28** | **3** | CD276 antigen |
| CD2AP | **16** | **0** | 2 | 0 | CD2-associated protein |
| CD44 | **187** | **18** | 186 | 79 | CD44 antigen |
| CD59 | 67 | 41 | **59** | **26** | CD59 glycoprotein |
| CD81 | **328** | **119** | **280** | **118** | CD81 |
| CD82 | **144** | **63** | 132 | 82 | CD82 antigen |
| CD97 | **24** | **5** | **15** | **4** | CD97 antigen |
| CDK1 | 13 | 8 | **15** | **6** | Cyclin-dependent kinase 1 |
| CHIMP2A | **82** | **36** | **80** | **18** | Charged multivesicular body protein 2a |
| CHMP1B | **17** | **6** | **19** | **3** | Charged multivesicular body protein 1b |
| CHMP2B | 13 | 9 | **12** | **0** | Charged multivesicular body protein 2b |
| CHMP3 | **12** | **5** | **12** | **2** | Charged multivesicular body protein 3 |
| CHMP4B | 11 | 7 | **10** | **2** | Charged multivesicular body protein 4b |
| CHMP5 | **28** | **8** | **27** | **6** | Charged multivesicular body protein 5 |
| CIB1 | **28** | **14** | **27** | **12** | Calcium and integrin-binding protein 1 |
| CKB | **19** | **0** | **28** | **2** | Creatine kinase B-type |
| CLDN7 | **112** | **52** | **91** | **38** | Claudin-7 |
| CLDN2 | **46** | **7** | **42** | **10** | Claudin-2 |
| CLDND1 | **16** | **6** | **12** | **6** | Claudin domain-containing protein 1 |
| CNP | **12** | **0** | **10** | **2** | 2',3'-cyclic-nucleotide 3'-phosphodiesterase |
| CPNE2 | **11** | **0** | **10** | **0** | Copine-2 |
| CPNE8 | **26** | **0** | **17** | **0** | Copine-8 |
| CSPG4 | **71** | **0** | **135** | **6** | Chondroitin sulfate proteoglycan 4 |
| SLC44A2 | **106** | **17** | **111** | **39** | Choline transporter-like protein 2 |
| CXCR4 | **43** | **0** | **38** | **1** | C-X-C chemokine receptor type 4 |
| CYFIP1 | **31** | **1** | **27** | **2** | Cytoplasmic FMR1-interacting protein 1 |
| CYSTM1 | **20** | **4** | **23** | **11** | Cysteine-rich and transmembrane domain-containing protein 1 |
| DIP2A | **32** | **0** | **20** | **0** | Disco-interacting protein 2 homolog A |
| DIP2B | **316** | **89** | **278** | **87** | Disco-interacting protein 2 homolog B |
| DIP2C | **31** | **0** | **19** | **0** | Disco-interacting protein 2 homolog C |
| DNAJA1 | 46 | 28 | **40** | **15** | DnaJ homolog subfamily A member 1 |
| DNASE1L1 | **21** | **5** | **26** | **4** | Deoxyribonuclease-1-like 1 |

Suppl. Table 1A cont.

| **name** | **protein hits** | | | | **full name** |
| --- | --- | --- | --- | --- | --- |
|  | **A818.4** | **A818.4-v6kd** | **Capan1** | **Capan1-v6kd** |  |
| DPEP1 | **23** | **0** | **20** | **4** | Dipeptidase 1 |
| DPP4 | **178** | **16** | 161 | 121 | Dipeptidyl peptidase 4=CD26 |
| EFNB1 | **112** | **13** | **106** | **34** | Ephrin-B1 |
| EHD2 | **10** | **0** | 8 | 0 | EH domain-containing protein 2 |
| EPHA2 | **84** | **16** | 56 | 36 | Ephrin type-A receptor 2 |
| EPHB2 | **60** | **0** | **46** | **18** | Ephrin type-B receptor 2 |
| EPHB3 | **201** | **9** | **130** | **19** | Ephrin type-B receptor 3 |
| EPHB4 | **154** | **0** | **122** | **0** | Ephrin type-B receptor 4 |
| EVA1B | **15** | **2** | **12** | **0** | Protein eva-1 homolog B |
| F5 | **28** | **6** | 21 | 12 | Coagulation factor V |
| FAM49B | **19** | **2** | **13** | **3** | Protein FAM49B |
| FARP1 | **26** | **0** | **19** | **0** | FERM, RhoGEF and pleckstrin domain-containing protein 1 |
| FMNL2 | **35** | **4** | **20** | **3** | Formin-like protein 2 |
| FTH1 | **13** | **0** | **24** | **0** | Ferritin heavy chain |
| FYN | **67** | **6** | **53** | **10** | Tyrosine-protein kinase Fyn |
| GCA | **11** | **0** | 9 | 5 | Grancalcin |
| GGT1 | **31** | **1** | **25** | **10** | Gamma-glutamyltranspeptidase 1 |
| GNA13 | **24** | **0** | 20 | 13 | Guanine nucleotide-binding protein subunit alpha-13 |
| GNAI1 | **118** | **37** | **129** | **24** | Guanine nucleotide-binding protein G(i) subunit alpha-1 |
| GNAI2 | 143 | 78 | **135** | **33** | Guanine nucleotide-binding protein G(i) subunit alpha-2 |
| GNAI3 | **112** | **40** | **109** | **26** | Guanine nucleotide-binding protein G(k) subunit alpha |
| GNB1 | 57 | 37 | **56** | **24** | Guanine nucleotide-binding protein G(I)/G(S)/G(T) subunit beta-1 |
| GNB2 | **63** | **31** | **57** | **26** | Guanine nucleotide binding proteinbeta polypeptide 2 |
| GRB2 | **25** | **9** | 21 | 12 | Growth factor receptor-bound protein 2 |
| GRPC5C | **25** | **1** | 12 | 7 | G-protein coupled receptor family C group 5 member C |
| GSLG1 | 8 | 0 | **12** | **5** | Golgi apparatus protein 1 |
| GSTP1 | **46** | **14** | **53** | **18** | Glutathione S-transferase P |
| H2AFY | **10** | **0** | **19** | **9** | Core histone macro-H2A.1 |
| HS3ST1 | **18** | **0** | **26** | **10** | Heparan sulfate glucosamine 3-O-sulfotransferase 1 |
| IGF2R | **53** | **7** | **20** | **9** | Cation-independent mannose-6-phosphate receptor |
| IGSF3 | **41** | **0** | 7 | 3 | Immunoglobulin superfamily member 3 |
| IGSF8 | **175** | **86** | **180** | **81** | Immunoglobulin superfamily member 8 |
| IST1 | **154** | **75** | **148** | **46** | increased sodium tolerance 1 homolog |
| ITGA1 | **49** | **21** | **51** | **12** | Integrin alpha-1 |
| ITGA2 | **105** | **36** | **126** | **61** | Integrin alpha-2 |
| ITGA3 | **89** | **33** | 80 | 59 | Integrin alpha-3 |
| ITGAV | 189 | 100 | **209** | **80** | Integrin alpha-V |
| ITGB4 | **297** | **118** | **214** | **100** | Integrin beta-4 |
| ITGB5 | **75** | **36** | **82** | **15** | Integrin beta-5 |
| ITGB6 | 5 | 0 | **12** | **0** | Integrin beta-6 |
| ITM2C | **18** | **3** | **15** | **4** | Integral membrane protein 2C |
| JAM1 | 53 | 35 | **44** | **21** | Junctional adhesion molecule A |
| LAMTOR2 | **19** | **7** | **13** | **6** | Ragulator complex protein LAMTOR2 |
| LGALS4 | **40** | **0** | **57** | **4** | Galectin-4 |
| LRRC57 | 13 | 9 | **14** | **7** | Leucine-rich repeat-containing protein 57 |
| LSR | **102** | **29** | 92 | 52 | Lipolysis-stimulated lipoprotein receptor |
| LYN | **34** | **0** | **22** | **10** | Tyrosine-protein kinase Lyn |
| LYSC | **10** | **2** | 10 | 6 | Lysozyme C |
| MAP2K4 | **71** | **0** | **52** | **0** | Mitogen-activated protein kinase 4 |
| MET | **12** | **0** | **10** | **0** | Hepatocyte growth factor receptor |
| MFGE8 | **210** | **45** | 229 | 134 | Lactadherin |
| MINK1 | **116** | **18** | **87** | **18** | Misshapen-like kinase 1 |
| MITD1 | **95** | **21** | **98** | **12** | MIT domain-containing protein 1 |
| MMP15 | **11** | **0** | **9** | **1** | Matrix metalloproteinase-15 |
| MSN | **192** | **101** | **231** | **90** | Moesin |
| MVB12A | **71** | **0** | **58** | **14** | Multivesicular body subunit 12A |
| MVB12B | **50** | **8** | **25** | **4** | Multivesicular body subunit 12B |
| MYO1C | **21** | **7** | 15 | 8 | Unconventional myosin-Ic |

Suppl. Table 1A cont.

| **name** | **protein hits** | | | | **full name** |
| --- | --- | --- | --- | --- | --- |
|  | **A818.4** | **A818.4-v6kd** | **Capan1** | **Capan1-v6kd** |  |
| MYO1D | **36** | **6** | **39** | **6** | Unconventional myosin-Id |
| NCKAP1 | **42** | **5** | **41** | **2** | Nck-associated protein 1 |
| NEDD4L | **19** | **4** | **17** | **4** | E3 ubiquitin-protein ligase NEDD4-like |
| NIBL1 | 17 | 9 | **13** | **6** | Niban-like protein 1 |
| NPTN | **15** | **2** | **13** | **3** | Neuroplastin |
| NT5E | **61** | **4** | **43** | **20** | 5'-nucleotidase |
| NTSR1 | **27** | **0** | **10** | **0** | Neurotensin receptor type 1 |
| PACSIN3 | **12** | **1** | **10** | **0** | Protein kinase C and casein kinase substrate in neurons protein 3 |
| PDCD6 | **107** | **54** | **109** | **49** | Programmed cell death protein 6 |
| PDCD6IP | **472** | **224** | 448 | 266 | Programmed cell death 6-interacting protein |
| PEBP1 | 15 | 9 | **16** | **7** | Phosphatidylethanolamine-binding protein 1 |
| PKP3 | **18** | **0** | **4** | **0** | Plakophilin-3 |
| PLEKHB2 | **26** | **9** | **24** | **6** | Pleckstrin homology domain-containing family B member 2 |
| PLPP2 | **10** | **0** | **9** | **3** | Lipid phosphate phosphohydrolase 2 |
| PLSCR3 | **72** | **11** | **69** | **14** | Phospholipid scramblase 3 |
| PLXNA1 | **12** | **3** | **5** | **2** | Plexin-A1 |
| PLXNA2 | **20** | **3** | **14** | **0** | Plexin-A2 |
| PLXNB2 | **52** | **24** | **50** | **8** | Plexin-B2 |
| PROCR | **12** | **0** | **10** | **5** | Protein C receptor |
| PROM1 | **242** | **11** | **195** | **36** | Prominin-1 |
| PROM2 | **80** | **0** | **89** | **11** | Prominin-2 |
| PTK7 | **39** | **3** | **15** | **0** | Inactive tyrosine-protein kinase 7 |
| PTP4A1 | **19** | **0** | **21** | **2** | Protein tyrosine phosphatase type IVA 1 |
| PTP4A2 | **16** | **7** | **14** | **0** | Protein tyrosine phosphatase type IVA 2 |
| PTPRF | **65** | **0** | **67** | **14** | Receptor-type tyrosine-protein phosphatase F |
| PVR | **13** | **0** | **6** | **0** | Poliovirus receptor |
| PVRL2 | **27** | **0** | **15** | **0** | Nectin-2 |
| RAB21 | 20 | 13 | **18** | **6** | Ras-related protein Rab-21 |
| RAB22A | **29** | **15** | **22** | **11** | Ras-related protein Rab-22A |
| RAB3D | **28** | **0** | **28** | **0** | Ras-related protein Rab-3D |
| RAB5C | **67** | **28** | **54** | **27** | Ras-related protein Rab-5C |
| RAC1 | **98** | **48** | **76** | **36** | Ras-related C3 botulinum toxin substrate 1 |
| RAC2 | **38** | **0** | **26** | **0** | Ras-related C3 botulinum toxin substrate 2 |
| RAP1A | 76 | 43 | **74** | **36** | Ras-related protein Rap-1A |
| RAP2A | **37** | **15** | **30** | **14** | Ras-related protein Rap-2a |
| RAP2C | **67** | **30** | **53** | **23** | Ras-related protein Rap-2c |
| ROR1 | **131** | **0** | **46** | **2** | Tyrosine-protein kinase transmembrane receptor ROR1 |
| RP2 | **20** | **11** | **22** | **8** | Protein XRP2 |
| RPS5 | **12** | **5** | **13** | **6** | 40S ribosomal protein S5 |
| RRAS | **35** | **8** | **22** | **11** | Ras-related protein R-Ras |
| RRAS2 | **44** | **9** | **29** | **12** | Ras-related protein R-Ras2 |
| S100-A16 | **23** | **5** | 20 | 15 | Protein S100-A16 |
| SCAMP3 | **86** | **31** | **72** | **30** | Secretory carrier-associated membrane protein 3 |
| SCARB1 | **13** | **3** | 15 | 9 | Scavenger receptor class B member 1 |
| SDCB1 | **384** | **116** | **381** | **130** | Syntenin-1 |
| SERC5 | **65** | **20** | **72** | **28** | Serine incorporator 5 |
| SERINC1 | **19** | **4** | 11 | 7 | Serine incorporator 1 |
| SHH | **26** | **2** | **34** | **4** | Sonic hedgehog protein |
| SLC12A2 | **242** | **76** | **214** | **99** | Solute carrier family 12 member 2 |
| SLC12A7 | **12** | **6** | **11** | **2** | Solute carrier family 12 member 7 |
| SLC29A1 | **36** | **4** | **24** | **7** | Equilibrative nucleoside transporter 1 |
| SLC29A2 | **13** | **3** | **4** | **2** | Equilibrative nucleoside transporter 2 |
| SLC39A10 | **41** | **0** | **35** | **0** | Zinc transporter ZIP10 |
| SLC43A1 | **29** | **0** | **17** | **0** | Large neutral amino acids transporter small subunit 3 |
| SLC44A1 | 111 | 78 | **112** | **44** | Choline transporter-like protein 1 |
| SLC44A4 | **20** | **0** | **14** | **6** | Choline transporter-like protein 4 |
| SLC6A6 | **25** | **6** | **17** | **7** | Sodium- and chloride-dependent taurine transporter |
| SLC6A8 | **29** | **1** | **12** | **0** | Sodium- and chloride-dependent creatine transporter 1 |

Suppl. Table 1A cont.

| **name** | **protein hits** | | | | **full name** |
| --- | --- | --- | --- | --- | --- |
|  | **A818.4** | **A818.4-v6kd** | **Capan1** | **Capan1-v6kd** |  |
| SLC7A1 | **35** | **13** | **10** | **0** | High affinity cationic amino acid transporter 1 |
| SLC7A11 | **15** | **0** | 4 | 0 | Cystine/glutamate transporter |
| SLITRK6 | **10** | **0** | **12** | **0** | SLIT and NTRK-like protein 6 |
| SMPDL3B | **26** | **0** | **17** | **8** | Acid sphingomyelinase-like phosphodiesterase 3b |
| SNAP23 | 13 | 9 | **14** | **3** | Synaptosomal-associated protein 23 |
| SNF8 | **34** | **8** | **36** | **4** | Vacuolar-sorting protein SNF8 |
| SNX18 | **12** | **0** | **13** | **0** | Sorting nexin-18 |
| SNX33 | **11** | **0** | **10** | **0** | Sorting nexin-33 |
| SORI | 42 | 30 | **34** | **15** | Sorcin |
| SRC | **46** | **0** | **37** | **8** | Proto-oncogene tyrosine-protein kinase Src |
| TMBIM1 | **18** | **1** | **16** | **4** | Transmembrane BAX inhibitor motif containing 1 |
| TMEM2 | **139** | **64** | **113** | **32** | Transmembrane protein 2 |
| TNIK | **91** | **27** | **64** | **0** | TRAF2 and NCK-interacting protein kinase |
| TOLIP | **49** | **4** | **38** | **7** | Toll-interacting protein |
| TOM1L1 | **57** | **12** | **58** | **14** | TOM1-like protein 1 |
| TS101 | **263** | **75** | **214** | **74** | Tumor susceptibility gene 101 protein |
| TSPAN1 | **221** | **55** | **169** | **84** | Tetraspanin-1 |
| TSPAN6 | **92** | **3** | **98** | **18** | Tetraspanin-6 |
| TSPAN8 | **73** | **8** | **84** | **22** | Tetraspanin-8 |
| TSPAN9 | **45** | **0** | **30** | **3** | Tetraspanin-9 |
| TSPAN14 | **123** | **28** | **100** | **42** | Tetraspanin-14 |
| TSPAN15 | **89** | **10** | **74** | **33** | Tetraspanin-151 |
| TSPAN33 | **15** | **0** | **12** | **3** | Tetraspanin-33 |
| TTYH3 | 51 | 36 | **54** | **19** | Protein tweety homolog 3 |
| UBB | **629** | **0** | **511** | **0** | Polyubiquitin-B |
| UBE2D3 | **73** | **10** | **59** | **5** | Ubiquitin-conjugating enzyme E2 variant 3 |
| UBE2V1 | **15** | **7** | **10** | **5** | Ubiquitin-conjugating enzyme E2 variant 1 |
| UBE2V2 | **16** | **7** | **12** | **0** | Ubiquitin-conjugating enzyme E2 variant 2 |
| UBL3 | **10** | **5** | **12** | **4** | Ubiquitin-like protein 3 |
| VP37B | **104** | **29** | **108** | **41** | Vacuolar protein sorting-associated protein 37B |
| VPS25 | **31** | **14** | **23** | **11** | Vacuolar protein-sorting-associated protein 25 |
| VPS28 | **136** | **35** | **110** | **33** | Vacuolar protein sorting-associated protein 28 homolog |
| VPS36 | **34** | **3** | **28** | **6** | Vacuolar protein-sorting-associated protein 36 |
| VPS4A | **38** | **10** | **32** | **0** | Vacuolar protein sorting-associated protein 4A |
| VPS4B | **57** | **12** | **48** | **6** | Vacuolar protein sorting-associated protein 4B |
| VSIG2 | **11** | **0** | **19** | **0** | V-set and immunoglobulin domain-containing protein 2 |
| VTA1 | **34** | **7** | **24** | **0** | Vacuolar protein sorting-associated protein VTA1 homolog |
| YES | **57** | **0** | **45** | **0** | Tyrosine-protein kinase Yes |
| ZDHHC5 | **15** | **6** | **10** | **4** | Palmitoyltransferase ZDHHC5 |
| A2M | **13** | **0** | 11 | 18 | Alpha-2-macroglobulin |
| ACP2 | **15** | **7** | 9 | 9 | Lysosomal acid phosphatase |
| ADAM9 | **11** | **2** | 2 | 4 | Disintegrin and metalloproteinase domain-containing protein 9 |
| API5 | **14** | **0** | nd | nd | Apoptosis inhibitor 5 |
| CD46 | **17** | **0** | 18 | 21 | Complement membrane cofactor protein |
| CD55 | **22** | **8** | 15 | 16 | Complement decay-accelerating factor |
| CEACAM5 | **14** | **0** | 10 | 14 | Carcinoembryonic antigen-related cell adhesion molecule 5 |
| EFNB2 | **50** | **0** | 26 | 27 | Ephrin-B2 |
| EPS8 | **10** | **0** | 3 | 8 | Epidermal growth factor receptor kinase substrate 8 |
| HBE1 | **21** | **0** | 30 | 30 | Hemoglobin subunit epsilon |
| HIST2H2AB | **11** | **0** | nd | nd | Histone H2A type 2-B |
| IFITM1 | **11** | **0** | nd | nd | Interferon-induced transmembrane protein 1 |
| P2RX4 | **12** | **0** | 6 | 9 | P2X purinoceptor 4 |
| PLSCR1 | **53** | **15** | 42 | 72 | Phospholipid scramblase 1 |
| PTPRA | **10** | **0** | 6 | 7 | Receptor-type tyrosine-protein phosphatase alpha |
| RAB11A | **54** | **0** | 55 | 58 | Ras-related protein Rab-11A |
| RAB25 | **17** | **0** | 16 | 17 | Ras-related protein Rab-25 |
| RPL10A | **36** | **13** | 39 | 28 | 60S ribosomal protein L10a |
| RPS16 | **41** | **17** | 42 | 46 | 40S ribosomal protein S16 |

Suppl. Table 1A cont.

| **name** | **protein hits** | | | | **full name** |
| --- | --- | --- | --- | --- | --- |
|  | **A818.4** | **A818.4-v6kd** | **Capan1** | **Capan1-v6kd** |  |
| S100A14 | **16** | **1** | 11 | 16 | Protein S100-A14 |
| SDCBP2 | **48** | **11** | 43 | 54 | Syntenin-2 |
| TM9SF3 | **15** | **2** | nd | nd | Transmembrane 9 superfamily member 3 |
| TSPAN3 | **29** | **6** | 25 | 38 | Tetraspanin-3 |
| ADGRG1 | 18 | 16 | **21** | **7** | G-protein coupled receptor 56 |
| AKR1B10 | nd | nd | **12** | **0** | Aldo-keto reductase family 1 member B10 |
| ANO6 | 10 | 12 | **12** | **0** | Anoctamin-6 |
| ATP1A2 | 106 | 100 | **109** | **0** | Sodium/potassium-transporting ATPase subunit alpha-2 |
| ATP2B3 | nd | nd | **20** | **0** | Plasma membrane calcium-transporting ATPase 3 |
| ATP6V0A1 | nd | nd | **10** | **0** | V-type proton ATPase 116 kDa subunit a isoform 1 |
| ATP6V0C | nd | nd | **17** | **3** | V-type proton ATPase 16 kDa proteolipid subunit |
| ATP6V0D1 | 8 | 8 | **15** | **3** | V-type proton ATPase subunit d 1 |
| CHIMP1A | 9 | 8 | **12** | **2** | Charged multivesicular body protein 1a |
| CLDN9 | nd | nd | **29** | **0** | Claudin-9 |
| CPNE3 | 10 | 8 | **12** | **0** | Copine-3 |
| EHD1 | 73 | 113 | **100** | **28** | EH domain-containing protein 1 |
| EPHA4 | nd | nd | **17** | **0** | Ephrin type-A receptor 4 |
| GDI2 | 61 | 74 | **58** | **22** | Rab GDP dissociation inhibitor beta |
| GOLGA7 | 13 | 12 | **13** | **6** | Golgin subfamily A member 7 |
| H6PD | 15 | 17 | **30** | **11** | Glucose-6-phosphate 1-dehydrogenase |
| HIST1H2BD | 57 | 53 | **87** | **41** | Histone H2B type 1-B |
| HIST1H2BE | 58 | 54 | **87** | **41** | Histone H2B type 1-C/E/F/G/I |
| HPRT | nd | nd | **11** | **4** | Hypoxanthine-guanine phosphoribosyltransferase |
| IHH | nd | nd | **11** | **0** | Indian hedgehog protein |
| LIN7C | 17 | 16 | **14** | **7** | Protein lin-7 homolog C |
| LMNA | 19 | 27 | **12** | **3** | Prelamin-A/C |
| MFI2 | 24 | 33 | **13** | **4** | Melanotransferrin |
| NDRG1 | nd | nd | **11** | **3** | N-myc downstream regulated 1 |
| PARVA | nd | nd | **12** | **0** | Alpha-parvin1 |
| PGAM1 | 11 | 17 | **18** | **7** | Phosphoglycerate mutase 1 |
| RAB12 | nd | nd | **19** | **0** | Ras-related protein Rab-12 |
| REG4 | nd | nd | **12** | **0** | Regenerating islet-derived protein 4 |
| RSU1 | 9 | 7 | **10** | **5** | Ras suppressor protein 1 |
| SLC1A4 | 38 | 39 | **26** | **11** | Neutral amino acid transporter A |
| TAGLN2 | 31 | 29 | **30** | **13** | Transgelin-2 |
| TSPAN5 | 17 | 14 | **15** | **1** | Tetraspanin-5 |
| VAMP2 | 17 | 13 | **10** | **0** | Vesicle-associated membrane protein 2 |
| YKT6 | 23 | 21 | **22** | **11** | Synaptobrevin homolog YKT6 |

Suppl. Table 1 continued

**Table 1B. Proteins upregulated in CD44v6^kd^ cells**

| **name** | **protein hits** | | | | **full name** |
| --- | --- | --- | --- | --- | --- |
|  | **A818.4** | **A818.4-v6kd** | **Capan1** | **Capan1-v6kd** |  |
| 1433S | **0** | **67** | **0** | **35** | 14-3-3 protein sigma |
| ACO1 | **0** | **16** | **0** | **3** | Cytoplasmic aconitate hydratase |
| ACTN1 | **27** | **149** | **33** | **66** | Alpha-actinin-1 |
| ACTN4 | **56** | **202** | 68 | 103 | Alpha-actinin-4 |
| ACTR1A | **5** | **15** | **6** | **14** | Alpha-centractin |
| ACTR1B | **0** | **11** | **0** | **8** | Beta-centractin |
| AHCY | **27** | **105** | **39** | **85** | Adenosylhomocysteinase |
| AHSG | **6** | **12** | 11 | 18 | Alpha-2-HS-glycoprotein |
| AIMP1 | 2 | 8 | **2** | **12** | Aminoacyl tRNA synthase complex-interacting multifunctional protein 1 |
| ANXA6 | **31** | **381** | **25** | **65** | Annexin A6 |
| APOA1 | 2 | 6 | **3** | **12** | Apolipoprotein A-I |
| APOB | **9** | **23** | **8** | **33** | Apolipoprotein B-100 |
| ARL8A | **9** | **18** | 11 | 18 | ADP-ribosylation factor-like protein 8A |
| ARL8B | 10 | 17 | **0** | **19** | ADP-ribosylation factor-like protein 8B |
| ARPC4 | **11** | **22** | **10** | **22** | Actin-related protein 2/3 complex subunit 4 |
| ASNS | **3** | **38** | **3** | **13** | Asparagine synthetase |
| ASS1 | **6** | **16** | **5** | **172** | Argininosuccinate synthase |
| AT1B3 | **10** | **44** | **9** | **20** | Sodium/potassium-transporting ATPase subunit beta-3 |
| ATPB | **16** | **52** | **17** | **70** | ATP synthase subunit beta, mitochondrial |
| C1QBP | 0 | 3 | **0** | **11** | Complement component 1 Q subcomponent-binding protein |
| CA6 | **0** | **11** | 0 | 6 | Carbonic anhydrase 6 |
| CAPNS1 | **12** | **30** | **13** | **26** | Calpain small subunit 1 |
| CATSD | **1** | **12** | 0 | 5 | Cathepsin D |
| CCT2 | **12** | **58** | **18** | **41** | T-complex protein 1 subunit beta |
| CCT3 | **8** | **40** | **11** | **38** | T-complex protein 1 subunit gamma |
| CCT4 | **8** | **54** | **6** | **35** | T-complex protein 1 subunit delta |
| CCT5 | **1** | **19** | **1** | **13** | T-complex protein 1 subunit epsilon |
| CCT6A | **10** | **46** | **14** | **31** | T-complex protein 1 subunit zeta |
| CCT7 | **7** | **53** | **6** | **29** | T-complex protein 1 subunit eta |
| CCT8 | **6** | **45** | **6** | **36** | T-complex protein 1 subunit theta |
| CEMIP | **7** | **23** | **7** | **51** | Cell migration-inducing and hyaluronan-binding protein |
| CLDN11 | **0** | **26** | 0 | 4 | Claudin-11 |
| CLIC1 | 35 | 61 | **37** | **79** | Chloride intracellular channel protein 1 |
| CLU | **8** | **102** | **19** | **46** | Clusterin |
| COPE | 10 | 18 | **13** | **30** | Coatomer subunit epsilon |
| COTL1 | **5** | **13** | **4** | **10** | Coactosin-like protein |
| CPSF7 | **2** | **10** | 3 | 7 | Cleavage and polyadenylation specificity factor subunit 7 |
| DARS | **1** | **35** | **6** | **55** | Aspartate--tRNA ligase, cytoplasmic |
| DERA | **3** | **11** | **4** | **16** | Deoxyribose-phosphate aldolase |
| DHX9 | **1** | **15** | **4** | **11** | ATP-dependent RNA helicase A |
| DPP1 | 0 | 4 | **0** | **16** | Dipeptidyl peptidase 1 |
| DPYSL2 | **0** | **97** | 0 | 7 | Dihydropyrimidinase-related protein 2 |
| DYNC1H1 | **0** | **10** | 0 | 4 | Cytoplasmic dynein 1 heavy chain 1 |
| EEF1E1 | **6** | **19** | **8** | **14** | Eukaryotic translation elongation factor 1 epsilon-1 |
| EEF1G | **35** | **106** | **40** | **85** | Elongation factor 1-gamma |
| EEF2 | **57** | **125** | 61 | 111 | Elongation factor 2 |
| EIF3A | **8** | **24** | **8** | **24** | Eukaryotic translation initiation factor 3 subunit A |
| EIF3B | **8** | **58** | **15** | **35** | Eukaryotic translation initiation factor 3 subunit B |
| EIF3E | **2** | **31** | **3** | **29** | Eukaryotic translation initiation factor 3 subunit E |
| EIF3F | **7** | **27** | **11** | **23** | Eukaryotic translation initiation factor 3 subunit F |
| EIF3L | **20** | **66** | **24** | **72** | Eukaryotic translation initiation factor 3 subunit L |
| EIF3M | **0** | **20** | **2** | **16** | Eukaryotic translation initiation factor 3 subunit M |
| EIF4A1 | **3** | **26** | **7** | **26** | Eukaryotic initiation factor 4A-I |
| ENO3 | **12** | **41** | **0** | **10** | Beta-enolase |
| EPHX1 | **3** | **14** | 4 | 8 | Epoxide hydrolase 1 |
| FAM3C | **2** | **23** | **6** | **21** | Protein FAM3C |
| FARSA | **6** | **27** | **11** | **30** | Phenylalanine--tRNA ligase alpha subunit |
| FIBCD1 | 0 | 3 | **0** | **23** | Fibrinogen C domain-containing protein 1 |

Suppl. Table 1B continued

| **name** | **protein hits** | | | | **full name** |
| --- | --- | --- | --- | --- | --- |
|  | **A818.4** | **A818.4-v6kd** | **Capan1** | **Capan1-v6kd** |  |
| FTH1 | **0** | **24** | **0** | **28** | Ferritin heavy chain |
| FTL | **0** | **17** | **0** | **7** | Ferritin light chain |
| GARS | **1** | **35** | **0** | **25** | Glycine--tRNA ligase |
| GPC4 | 1 | 4 | **1** | **28** | Glypican-4 |
| GSN | **7** | **67** | **16** | **33** | Gelsolin |
| HNRNPC | 6 | 11 | **9** | **24** | Heterogeneous nuclear ribonucleoproteins C1/C2 |
| HNRNPK | **4** | **29** | **6** | **18** | Heterogeneous nuclear ribonucleoprotein K |
| HS90AA1 | **122** | **268** | **139** | **296** | Heat shock protein HSP 90-alpha |
| HS90AB2P | 158 | 263 | **176** | **365** | Heat shock protein HSP 90-beta |
| HSP90B1 | **0** | **20** | **0** | **107** | Endoplasmin |
| HSPB1 | **4** | **27** | **7** | **16** | Heat shock protein beta-1 |
| HSPD1 | 1 | 4 | **3** | **25** | 60 kDa heat shock protein, mitochondrial |
| ICAM1 | **26** | **56** | **12** | **29** | Intercellular adhesion molecule 1 |
| ITGA5 | **0** | **41** | 0 | 3 | Integrin alpha-5 |
| ITM2B | 3 | 6 | **2** | **26** | Integral membrane protein 2B |
| KPNB1 | 7 | 11 | **12** | **21** | Importin subunit beta-1 |
| LDHC | **0** | **14** | **0** | **17** | L-lactate dehydrogenase C chain |
| LGALS3 | **6** | **43** | **2** | **33** | Galectin-3 |
| LGALS3BP | 373 | 482 | **391** | **789** | Galectin-3-binding protein |
| LNPEP | **6** | **38** | **5** | **10** | Leucyl-cystinyl aminopeptidase |
| MDH2 | **0** | **35** | **2** | **12** | Malate dehydrogenase, mitochondrial |
| MYH9 | 0 | 8 | **1** | **23** | Myosin-9 |
| MYL12A | 18 | 26 | 15 | 24 | Myosin regulatory light chain 12A |
| MYOF | **6** | **75** | 0 | 7 | Myoferlin |
| NANS | **2** | **13** | 4 | 9 | Sialic acid synthase |
| NONO | **0** | **13** | **0** | **11** | Non-POU domain-containing octamer-binding protein |
| NPEPPS | **6** | **27** | **4** | **11** | Puromycin-sensitive aminopeptidase |
| NQO1 | **2** | **21** | **6** | **24** | Quinone oxidoreductase |
| OR10X1 | **2** | **15** | **5** | **10** | Olfactory receptor 10X1 |
| PAI1 | **0** | **77** | **0** | **23** | Plasminogen activator inhibitor 1 |
| PCNA | **0** | **16** | **0** | **15** | Proliferating cell nuclear antigen |
| PDXK | **0** | **13** | 0 | 5 | Pyridoxal kinase |
| PGK1 | **66** | **200** | **71** | **152** | Phosphoglycerate kinase 1 |
| PKM | **142** | **296** | **174** | **351** | Pyruvate kinase PKM |
| PLEC | **0** | **10** | 0 | 4 | Plectin |
| PP1R7 | **12** | **60** | **12** | **35** | Protein phosphatase 1 regulatory subunit 7 |
| PPCS | **5** | **10** | 10 | 16 | Phosphopantothenate--cysteine ligase |
| PRMT1 | **3** | **12** | **9** | **33** | Protein arginine N-methyltransferase 1 |
| PSMA1 | 11 | 20 | 17 | 31 | Proteasome subunit alpha type-1 |
| PSMA4 | 17 | 24 | 17 | 26 | Proteasome subunit alpha type-4 |
| PSMB1 | **12** | **27** | **22** | **45** | Proteasome subunit beta type-1 |
| PSMB2 | **10** | **30** | **10** | **30** | Proteasome subunit beta type-2 |
| PSMB8 | **0** | **11** | **0** | **19** | Proteasome subunit beta type-8 |
| PSMB9 | 0 | 9 | **0** | **15** | Proteasome subunit beta type-9 |
| PSMC3 | **5** | **12** | **4** | **22** | 26S protease regulatory subunit 6A |
| PSMD1 | **8** | **19** | **12** | **34** | 26S proteasome non-ATPase regulatory subunit 1 |
| PSMD11 | **14** | **36** | **19** | **52** | 26S proteasome non-ATPase regulatory subunit 11 |
| PSMD13 | **9** | **24** | **14** | **39** | 26S proteasome non-ATPase regulatory subunit 13 |
| PSMD14 | **3** | **18** | **9** | **18** | 26S proteasome non-ATPase regulatory subunit 14 |
| PSMD2 | **41** | **111** | **52** | **104** | 26S proteasome non-ATPase regulatory subunit 2 |
| PSMD3 | 25 | 49 | **22** | **77** | 26S proteasome non-ATPase regulatory subunit 3 |
| PSMD4 | **2** | **12** | **3** | **22** | 26S protease regulatory subunit 4 |
| PSMD6 | **15** | **33** | 22 | 38 | 26S proteasome non-ATPase regulatory subunit 6 |
| PSMD7 | 0 | 4 | **0** | **10** | 26S protease regulatory subunit 7 |
| PSMD8 | 0 | 4 | **0** | **20** | 26S protease regulatory subunit 8 |
| PSME2 | **4** | **12** | 0 | 6 | Proteasome activator complex subunit 2 |
| PTTG1IP | **26** | **56** | **7** | **24** | Pituitary tumor-transforming gene 1 protein-interacting protein |
| PYGL | **28** | **182** | **35** | **93** | Glycogen phosphorylase, liver |

Suppl. Table 1B continued

| **name** | **protein hits** | | | | | | | | **full name** | |
| --- | --- | --- | --- | --- | --- | --- | --- | --- | --- | --- |
|  | **A818.4** | | **A818.4-v6kd** | | **Capan1** | | **Capan1-v6kd** | |  | |
| RBBP7 | **0** | | **12** | | 0 | | 8 | | Histone-binding protein RBBP7 | |
| RBP1 | **0** | | **23** | | 0 | | 6 | | Retinol-binding protein 1 | |
| REEP5 | 2 | | 8 | | **5** | | **13** | | Receptor expression-enhancing protein 5 | |
| RHOF | 3 | | 7 | | **2** | | **11** | | Rho-related GTP-binding protein RhoF | |
| RPL14 | 3 | | 7 | | **4** | | **14** | | 60S ribosomal protein L14 | |
| RPL26 | **0** | | **13** | | 11 | | 19 | | 60S ribosomal protein L26 | |
| RPL5 | **7** | | **16** | | **10** | | **30** | | 60S ribosomal protein L5 | |
| RPL9 | 2 | | 8 | | **5** | | **11** | | 60S ribosomal protein L9 | |
| RPLA0 | **24** | | **57** | | **26** | | **58** | | 60S acidic ribosomal protein P0 | |
| RPS27A | **0** | | **230** | | **0** | | **329** | | Ubiquitin-40S ribosomal protein S27a | |
| RPSA | 39 | | 69 | | **48** | | **99** | | 40S ribosomal protein SA | |
| RUVBL1 | 36 | | 61 | | **37** | | **78** | | RuvB-like 1 | |
| RUVBL2 | **30** | | **61** | | **40** | | **81** | | RuvB-like 2 | |
| S100A4 | **0** | | **22** | | 0 | | 3 | | Protein S100-A4 | |
| SARS | **3** | | **16** | | **9** | | **26** | | Serine--tRNA ligase, cytoplasmic | |
| SEPT6 | **3** | | **12** | | **5** | | **11** | | Septin-6 | |
| SERPINA7 | 1 | | 4 | | **1** | | **12** | | serpin peptidase inhibitor, clade A | |
| SERPINF1 | **10** | | **26** | | **23** | | **53** | | serpin peptidase inhibitor, clade F | |
| SLC16A3 | **31** | | **85** | | **13** | | **47** | | Monocarboxylate transporter 4 | |
| SLC2A3 | **29** | | **162** | | 24 | | 39 | | Solute carrier family 2, facilitated glucose transporter member 3 | |
| SLC5A3 | **0** | | **41** | | 0 | | 4 | | Sodium/myo-inositol cotransporter | |
| SMC1A | **0** | | **10** | | **0** | | **11** | | Structural maintenance of chromosomes protein 1A | |
| SPTAN1 | 2 | | 6 | | **5** | | **25** | | Spectrin alpha chain, non-erythrocytic 1 | |
| SUPT6H | 3 | | 8 | | **9** | | **22** | | Transcription elongation factor SPT6 | |
| TCP1 | **8** | | **75** | | **17** | | **50** | | T-complex protein 1 subunit alpha | |
| TGFBI | **14** | | **179** | | **9** | | **42** | | Transforming growth factor-beta-induced protein ig-h3 | |
| TINAGL1 | **8** | | **33** | | **21** | | **98** | | Tubulointerstitial nephritis antigen-like | |
| TP53RK | **0** | | **10** | | 3 | | 6 | | TP53-regulating kinase | |
| TUBA1A | **52** | | **162** | | **64** | | **162** | | Tubulin alpha-1A chain | |
| TUBA1B | **63** | | **169** | | **75** | | **167** | | Tubulin alpha-1B chain | |
| TUBA4A | **0** | | **104** | | **0** | | **99** | | Tubulin alpha-4A chain | |
| TUBB | **20** | | **97** | | **22** | | **97** | | Tubulin beta class I | |
| TUBB4B | ***20*** | | **122** | | **0** | | **111** | | Tubulin beta-4B chain | |
| UBA1 | **12** | | **50** | | **9** | | **30** | | Ubiquitin-like modifier-activating enzyme 1 | |
| UGP2 | **2** | | **12** | | 2 | | 5 | | UTP--glucose-1-phosphate uridylyltransferase | |
| WARS | **8** | | **37** | | **7** | | **61** | | Tryptophan--tRNA ligase, cytoplasmic | |
| XRCC6 | **2** | | **60** | | **11** | | **26** | | X-ray repair cross-complementing protein 6 | |
| ACTR2 | | **8** | | **22** | | 12 | | 13 | | Actin-related protein 2 |
| ACTR3 | | **4** | | **10** | | 8 | | 5 | | Actin-related protein 3 |
| AKR7A2 | | **1** | | **10** | | 2 | | 3 | | Aflatoxin B1 aldehyde reductase member 2 |
| ALDOA | | **29** | | **59** | | 28 | | 20 | | Fructose-bisphosphate aldolase A |
| ANKH | | **0** | | **10** | | nd | | nd | | Progressive ankylosis protein homolog |
| AP1S1 | | **5** | | **11** | | 10 | | 11 | | AP-1 complex subunit sigma-1A |
| ARHGDIA | | **5** | | **11** | | 5 | | 6 | | Rho GDP-dissociation inhibitor 1 |
| ARPC2 | | **9** | | **30** | | 18 | | 20 | | Actin-related protein 2/3 complex subunit 2 |
| CAND1 | | **5** | | **28** | | 1 | | 0 | | Cullin-associated NEDD8-dissociated protein 1 |
| CD109 | | **0** | | **14** | | nd | | nd | | CD109 antigen |
| CD99 | | **5** | | **13** | | 2 | | 3 | | CD99 antigen |
| CTNNA1 | | **2** | | **24** | | nd | | nd | | Catenin alpha-1 |
| CTNNB1 | | **0** | | **45** | | nd | | nd | | Catenin beta-1 |
| CXADR | | **18** | | **55** | | 18 | | 12 | | Coxsackievirus and adenovirus receptor |
| DKK4 | | **0** | | **14** | | nd | | nd | | Dickkopf-related protein 4 |
| DSP | | **0** | | **12** | | nd | | nd | | Desmoplakin |
| EEF1A2 | | **0** | | **64** | | nd | | nd | | Elongation factor 1-alpha 2 |
| ENO1 | | **65** | | **262** | | 79 | | 72 | | Alpha-enolase |
| ENO2 | | **13** | | **41** | | nd | | nd | | Gamma-enolase |
| FLNA | | **2** | | **13** | | 10 | | 6 | | Filamin-A |
| GANAB | | **3** | | **28** | | 9 | | 11 | | Neutral alpha-glucosidase AB |

Suppl. Table 1B continued

| **name** | **protein hits** | | | | **full name** |
| --- | --- | --- | --- | --- | --- |
|  | **A818.4** | **A818.4-v6kd** | **Capan1** | **Capan1-v6kd** |  |
| GGCT | **4** | **11** | 4 | 3 | Gamma-glutamylcyclotransferase |
| GGH | **0** | **24** | 0 | 1 | Gamma-glutamyl hydrolase |
| GLOD4 | **3** | **13** | 3 | 1 | Glyoxalase domain-containing protein 4 |
| GOT1 | **3** | **11** | 6 | 0 | Aspartate aminotransferase, cytoplasmic |
| GSS | **1** | **13** | 4 | 2 | Glutathione synthetase |
| GSTO1 | **6** | **17** | 11 | 7 | Glutathione S-transferase omega-1 |
| HADHA | **0** | **17** | nd | nd | Hydroxyacyl-coenzyme A dehydrogenase, mitochondrial |
| HSPA4 | **0** | **26** | nd | nd | Heat shock 70 kDa protein 4 |
| HSPH1 | **1** | **10** | nd | nd | Heat shock protein 105 kDa |
| IFITM3 | **0** | **19** | nd | nd | Interferon-induced transmembrane protein 3 |
| LDHA | **52** | **157** | 117 | 124 | L-lactate dehydrogenase A chain |
| LDHB | **41** | **126** | 106 | 92 | L-lactate dehydrogenase B chain |
| LGALS7 | **0** | **13** | nd | nd | Galectin-7 |
| LTA4H | **0** | **10** | nd | nd | Leukotriene A-4 hydrolase |
| MAN1A1 | **2** | **10** | 3 | 0 | Mannosyl-oligosaccharide 1,2-alpha-mannosidase IA |
| MCMDC2 | **4** | **49** | 4 | 2 | MCM domain-containing protein 2 |
| NAPA | **3** | **10** | 8 | 9 | Alpha-soluble NSF attachment protein |
| OLA1 | **5** | **11** | 4 | 5 | Obg-like ATPase 1 |
| PA2G4 | **0** | **12** | 0 | 1 | Proliferation-associated protein 2G4 |
| PAFAH1B3 | **5** | **10** | 7 | 6 | Platelet-activating factor acetylhydrolase IB subunit gamma |
| PAICS | **5** | **18** | 7 | 9 | Multifunctional protein ADE2 |
| PARK7 | **4** | **32** | 3 | 0 | Protein deglycase DJ-1 |
| PLP2 | **9** | **21** | 5 | 5 | Proteolipid protein 2 |
| PRDX2 | **17** | **37** | 27 | 22 | Peroxiredoxin-2 |
| PRDX4 | **0** | **13** | nd | nd | Peroxiredoxin-4 |
| PRDX6 | **13** | **34** | 20 | 25 | Peroxiredoxin-6 |
| PSME1 | **1** | **15** | 3 | 1 | Proteasome activator complex subunit 1 |
| PSME3 | **4** | **25** | 11 | 11 | Proteasome activator complex subunit 3 |
| PUR9 | **2** | **19** | 9 | 9 | Bifunctional purine biosynthesis protein PURH |
| QDPR | **4** | **21** | 7 | 5 | Dihydropteridine reductase |
| RBBP4 | **2** | **16** | 5 | 7 | Histone-binding protein RBBP4 |
| S100A13 | **2** | **11** | 6 | 7 | Protein S100-A13 |
| SERPINB3 | **0** | **16** | nd | nd | Serpin B3 |
| SERPINB4 | **0** | **12** | nd | nd | Serpin B4 |
| SHMT2 | **0** | **22** | nd | nd | Serine hydroxymethyltransferase, mitochondrial |
| SLC7A6 | **0** | **18** | nd | nd | Y+L amino acid transporter 2 |
| SNRPD3 | **4** | **11** | 7 | 5 | Small nuclear ribonucleoprotein Sm D3 |
| STRA6 | **0** | **12** | nd | nd | Stimulated by retinoic acid gene 6 protein homolog |
| SYT1 | **0** | **21** | nd | nd | Synaptotagmin-1 |
| TALDO1 | **4** | **13** | 3 | 4 | Transaldolase |
| TMEM106B | **4** | **13** | 8 | 11 | Transmembrane protein 106B |
| TUBB2A | **0** | **78** | nd | nd | Tubulin beta-2A chain |
| TUBB3 | **0** | **86** | nd | nd | Tubulin beta-3 chain |
| TXD17 | **5** | **10** | 6 | 4 | Thioredoxin domain-containing protein 171 |
| VATD | **4** | **24** | 10 | 7 | V-type proton ATPase subunit D |
| VATE1 | **3** | **10** | 2 | 3 | V-type proton ATPase subunit E 1 |
| XPO2 | **10** | **21** | 15 | 13 | Exportin-2 |
| XRCC5 | **28** | **73** | 29 | 35 | X-ray repair cross-complementing protein 5 |
| ACTBL2 | nd | nd | **0** | **90** | Beta-actin-like protein 2 |
| ANO1 | nd | nd | **0** | **12** | Anoctamin-1 |
| AP1M1 | nd | nd | **1** | **10** | AP-1 complex subunit mu-1 |
| BCAP31 | nd | nd | **2** | **12** | B-cell receptor-associated protein 31 |
| BST2 | 17 | 20 | **0** | **15** | Bone marrow stromal antigen 2 |
| CCBL1 | nd | nd | **0** | **19** | L-amino-acid oxidase |
| CEACAM6 | 4 | 3 | **3** | **22** | Carcinoembryonic antigen-related cell adhesion molecule 6 |
| COPA | nd | nd | **1** | **14** | Coatomer subunit alpha |
| DLAT | 0 | 2 | **0** | **13** | Dihydrolipoyllys.-residue acetyltransferase comp of pyruv. dehydrog. |
| GCNT3 | nd | nd | **3** | **14** | β-galactosyl-O-glycosyl-glycoprotein β-N-acetylglucosaminyltransf.3 |

Suppl. Table 1B continued

| **name** | **protein hits** | | | | **full name** |
| --- | --- | --- | --- | --- | --- |
|  | **A818.4** | **A818.4-v6kd** | **Capan1** | **Capan1-v6kd** |  |
| GLUD1 | 1 | 2 | **2** | **16** | Glutamate dehydrogenase 1, mitochondrial |
| GPC1 | nd | nd | **0** | **35** | Glypican-1 |
| GRP78 | 43 | 49 | **36** | **61** | 78 kDa glucose-regulated protein |
| HSPG2 | 5 | 2 | **6** | **58** | Basement membrane-specific heparan sulfate proteoglycan |
| IARS | nd | nd | **0** | **10** | Isoleucine--tRNA ligase, cytoplasmic |
| ITIH3 | 6 | 9 | **15** | **40** | Inter-alpha-trypsin inhibitor heavy chain H3 |
| KLK10 | nd | nd | **0** | **18** | Kallikrein-10 |
| LAMA5 | nd | nd | **0** | **35** | Laminin subunit alpha-5 |
| LAMC1 | nd | nd | **0** | **17** | Laminin subunit gamma-1 |
| LGALS9B | nd | nd | **0** | **10** | Galectin-9B |
| LOXL2 | nd | nd | **0** | **10** | Lysyl oxidase homolog 2 |
| MUC1 | nd | nd | **0** | **30** | Mucin-1 |
| MVP | 169 | 107 | **230** | **727** | Major vault protein |
| MYH14 | nd | nd | **0** | **43** | Myosin-14 |
| NCL | nd | nd | **4** | **134** | Nucleolin |
| NPM1 | nd | nd | **12** | **102** | Nucleophosmin |
| PDIA3 | 6 | 6 | **3** | **53** | Protein disulfide-isomerase A3 |
| PPIB | 10 | 8 | **8** | **23** | Peptidyl-prolyl cis-trans isomerase B |
| PRPF19 | 6 | 7 | **5** | **13** | Pre-mRNA-processing factor 19 |
| PRSS23 | 5 | 2 | **7** | **31** | Serine protease 23 |
| PSMB3 | 9 | 8 | **10** | **22** | Proteasome subunit beta type-3 |
| RAB4B | nd | nd | **0** | **23** | Ras-related protein Rab-4B |
| RARS | nd | nd | **1** | **13** | Arginine--tRNA ligase, cytoplasmic |
| RPL15 | 3 | 1 | **5** | **12** | 60S ribosomal protein L15 |
| RPL26L | 9 | 11 | **0** | **18** | 60S ribosomal protein L26-like 1 |
| RPL32 | 6 | 4 | **4** | **15** | 60S ribosomal protein L32 |
| RPL7 | 5 | 4 | **13** | **31** | 60S ribosomal protein L7 |
| RPL7A | nd | nd | **5** | **10** | 60S ribosomal protein L7a |
| RPS11 | 6 | 5 | **7** | **19** | 40S ribosomal protein S11 |
| RPS23 | 4 | 3 | **4** | **13** | 40S ribosomal protein S23 |
| RPS3 | 25 | 19 | **25** | **64** | 40S ribosomal protein S3 |
| RPS4X | 3 | 0 | **5** | **13** | 40S ribosomal protein S4, X isoform |
| RPS9 | 25 | 13 | **32** | **71** | 40S ribosomal protein S9 |
| RTN3 | nd | nd | **9** | **33** | Reticulon-3 |
| RTN4 | 6 | 2 | **11** | **34** | Reticulon-4 |
| SDC4 | 6 | 4 | **7** | **40** | Syndecan-4 |
| SERPINB5 | nd | nd | **0** | **15** | Serpin peptidase inhibitor, clade B |
| SF3B3 | 14 | 17 | **3** | **30** | Splicing factor 3B subunit 3 |
| SMARCA5 | nd | nd | **0** | **11** | matrix-associated actin-dep. regulator chromatin subfamily A5 |
| SYEP | 0 | 2 | **0** | **10** | Bifunctional glutamate/proline--tRNA ligase |
| THBS1 | nd | nd | **0** | **16** | Thrombospondin-1 |
| TIMP1 | 0 | 1 | **2** | **10** | Metalloproteinase inhibitor 1 |
| TM9SF2 | 8 | 4 | **12** | **33** | Transmembrane 9 superfamily member 2 |
| TMEM87A | 3 | 1 | **9** | **19** | Transmembrane protein 87A |

Suppl. Table 1 continued

**Table 1C. Proteins opposingly regulated in CD44v6^kd^ cells**

| **name** | **protein hits** | | | | **full name** |
| --- | --- | --- | --- | --- | --- |
|  | **A818.4** | **A818.4-v6kd** | **Capan1** | **Capan1-v6kd** |  |
| AGRN | **26** | **3** | **13** | **245** | Agrin |
| CD47 | **26** | **8** | **18** | **40** | Leukocyte surface antigen CD47 |
| COPT1 | **11** | **0** | **5** | **10** | High affinity copper uptake protein 1 |
| CRABP2 | 14 | 25 | **17** | **8** | Cellular retinoic acid-binding protein 2 |
| HPRT | **5** | **19** | **11** | **5** | Hypoxanthine-guanine phosphoribosyltransferase |
| IFM1 | **11** | **0** | **10** | **39** | Interferon-induced transmembrane protein 1 |
| ISG15 | 7 | 2 | **2** | **14** | Ubiquitin-like protein ISG15 |
| LMAN2 | 6 | 1 | **2** | **10** | Vesicular integral-membrane protein VIP36 |
| MMP7 | **10** | **0** | **18** | **58** | Matrilysin |
| MUC13 | **16** | **0** | **8** | **26** | Mucin-13 |
| RAB12 | **0** | **19** | **19** | **0** | Ras-related protein Rab-12 |
| S100A6 | **16** | **7** | 15 | 29 | Protein S100-A6 |
| SLC31A1 | **11** | **0** | **5** | **10** | High affinity copper uptake protein 1 |
| TKT | **7** | **16** | **9** | **0** | Transketolase 3 |
| VATL | **6** | **15** | **17** | **3** | V-type proton ATPase 16 kDa proteolipid subunit |
| VINC | **11** | **25** | **9** | **0** | Vinculin |

Shown are significant protein hits that are strongly reduced or increased in A818.4- and Capan1-CD44v6^kd^ (green) or in A818.4-CD44v6^kd^ (red) or in Capan1-CD44v6^kd^ (blue) cells. Opposingly regulated proteins are indicated in violet. Differences in protein hits above 10 and above 2-fold are bold. Differences in MHC molecules are not shown.

Supplementary Table 3

**Antibodies and Reagents**

**A. Antibodies**

Antibody origin supplier

Actin mouse Becton Dickinson, HD, G

ADAM10 rabbit Santa Cruz, HD, G

ADAM17 (TACE) rabbit Santa Cruz, HD, G

ADAMTS5 rabbit Santa Cruz, HD, G

CD9 mouse ImmunoTools, Friesoythe, G

CD11b mouse Becton Dickinson, HD, G

CD26 mouse ImmunoTools, Friesoythe, G

CD29 mouse Becton Dickinson, HD, G

CD31 rat Becton Dickinson, HD, G

CD44 (25-32) mouse ref [1]

CD44v6 (vFF18) mouse ref [2]

CD49c mouse Becton Dickinson, HD, G

CD49f mouse Becton Dickinson, HD, G

CD63 mouse Becton Dickinson, HD, G

CD81 mouse Becton Dickinson, HD, G

CD104 rabbit Becton Dickinson, HD, G

CD133 rabbit Becton Dickinson, HD, G

CD151 (11B1) mouse ref [3]

CD184 (CXCR4) mouse Becton Dickinson, HD, G

Cld7 guinea pig ref [4]

Coll I rabbit Rockland, Gilbertsville, PA

Coll IV rabbit Rockland, Gilbertsville, PA

E-cadherin mouse Becton Dickinson, HD, G

EpCAM (HEA125) mouse ref [5]

EphA4 rabbit Santa Cruz, HD, G

Ezrin rabbit Sigma, Munich, G

FAK rabbit Cell Signaling, Fankfurt, G

FN mouse Becton Dickinson, HD, G

Gr1 mouse Becton Dickinson, HD, G

HAS3 mouse Santa Cruz, HD, G

Hyal1 rabbit Santa Cruz, HD, G

Hyal2 rabbit Santa Cruz, HD, G

Hyal3 rabbit Santa Cruz, HD, G

LNα1 rabbit Rockland, Gilbertsville, PA

LNα3 rabbit Rockland, Gilbertsville, PA

Lyve rabbit Santa Cruz, HD, G

MDR mouse Becton Dickinson, HD, G

MET mouse Cell Signaling, Frankfurt, G

MMP2 rabbit Dianova, Hamburg, G

MMP3 rabbit Santa Cruz, HD, G

MMP7 rabbit Santa Cruz, HD, G

MMP9 rabbit Dianova, Hamburg, G

MMP13 rabbit Dianova, Hamburg, G

MMP14 rabbit Santa Cruz, HD, G

N-Cadherin mouse Becton Dickinson, HD, G

PDGFR1 mouse Becton Dickinson, HD, G

PDGFR3 mouse Becton Dickinson, HD, G

P-ezrin rabbit Santa Cruz, HD, G

P-FAK rabbit Cell Signaling, Frankfurt, G

P-src rabbit Cell Signaling, Frankfurt, G

Src rabbit Santa Cruz, HD, G

Syndecan rabbit Santa Cruz, HD, G

TIMP1 mouse Santa Cruz, HD, G

TIMP2 mouse Santa Cruz, HD, G

TNFR1 mouse Becton Dickinson, HD, G

TNFR2 rat Santa Cruz, HD, G

TRAIL rabbit Santa Cruz, HD, G

Tspan8 (CO029) mouse ref [6]

UPA mouse Calbiochem, Darmstadt, G

UPAR mouse Calbiochem, Darmstadt, G

VEGFR2 mouse Becton Dickinson, HD, G

VEGFR3 rabbit Santa Cruz, HD, G

vimentin mouse Becton Dickinson, HD, G

Suppl.Table 3 continued

dye or biotin labeled secondary antibodies /

Streptavidin Dianova, Becton Dickinson, Amersham

**B. Reagents**

AnnexinV-FITC / -APC variable Becton Dickinson, HD, G

Cisplatin 5-30µg/ml Sigma, Munich, G

Coll I 10µg/ml Sigma Munich, G

Coll IV 10µg/ml Sigma Munich, G

FN 2µg/ml Sigma Munich, G

LN111 1µg/ml Sigma Munich, G

LN332 10µg/ml 804G (ref [7])*

Matrigel invasion: 1:5 Becton Dickinson, HD, G

Phalloidin 0.5µg/ml Becton Dickinson, HD, G

PI variable Becton Dickinson, HD, G

PMA 10^-8^M Sigma Munich, G

*804G cell culture supernatant was used as source of LN332. 804G cells were cultured (48 hours) in serum-free medium. Cleared supernatants (2x10min, 500g; 1x20min, 2000g; 1x30 min, 10,000g; 1x90min, 100,000g) were centrifuged for vesicle depletion and were concentrated. These serum-free, vesicle-depleted supernatants, highly enriched for LN332, are for brevity referred to as LN332.

References

1.Zuckermann FA, Binns RM, Husmann R, Yang H, Carr MM, Kim YB, [Davis WC](http://www.ncbi.nlm.nih.gov/pubmed?term=Davis WC%5BAuthor%5D&cauthor=true&cauthor_uid=7531912), [Misfeldt M](http://www.ncbi.nlm.nih.gov/pubmed?term=Misfeldt M%5BAuthor%5D&cauthor=true&cauthor_uid=7531912), [Lunney JK](http://www.ncbi.nlm.nih.gov/pubmed?term=Lunney JK%5BAuthor%5D&cauthor=true&cauthor_uid=7531912). Analysis of monoclonal antibodies reactive with porcine CD44 and CD45. Vet Immunol Immunopathol. 1993; 43: 293-305.

2. Seiter S, Tilgen W, Herrmann K, Schadendorf D, Patzelt E, Möller P, Zöller M. Expression of CD44 splice variants in human skin and epidermal tumours. Virchows Arch. 1996; 428: 141-149.

3. Geary SM, Cambareri AC, Sincock PM, Fitter S, Ashman LK. Differential tissue expression of epitopes of the tetraspanin CD151 recognised by monoclonal antibodies. Tissue Antigens 2001; 58: 1411-53.

4. Ladwein M, Pape UF, Schmidt DS, Schnölzer M, Fiedler S, Langbein L, Franke WW, Moldenhauer G, Zöller M. [The cell-cell adhesion molecule EpCAM interacts directly with the tight junction protein claudin-7.](http://www.ncbi.nlm.nih.gov/pubmed/16054130) Exp Cell Res. 2005; 309: 345-357.

5. Momburg F, Moldenhauer G, Hämmerling GJ, Möller P. Immunohistochemical study of the expression of a Mr 34,000 human epithelium-specific surface glycoprotein in normal and malignant tissues. Cancer Res. 1987; 47: 2883-2891.

6. Sela BA, Steplewski Z, Koprowski H. Colon carcinoma-associated glycoproteins recognized by monoclonal antibodies CO-029 and GA22-2. Hybridoma. 1989; 8: 481-491.

7. Homma Y, Ozono S, Numata I, Seidenfeld J, and Oyasu R (1985). α-Difluoromethylornithine inhibits cell growth stimulated by a tumor-promoting rat urinary fraction. Carcinogenesis. 1985; 6: 159-161.
